# Development of a Novel Lead that Targets *M. tuberculosis* Polyketide Synthase 13

**DOI:** 10.1016/j.cell.2017.06.025

**Published:** 2017-07-13

**Authors:** Anup Aggarwal, Maloy K. Parai, Nishant Shetty, Deeann Wallis, Lisa Woolhiser, Courtney Hastings, Noton K. Dutta, Stacy Galaviz, Ramesh C. Dhakal, Rupesh Shrestha, Shoko Wakabayashi, Chris Walpole, David Matthews, David Floyd, Paul Scullion, Jennifer Riley, Ola Epemolu, Suzanne Norval, Thomas Snavely, Gregory T. Robertson, Eric J. Rubin, Thomas R. Ioerger, Frik A. Sirgel, Ruben van der Merwe, Paul D. van Helden, Peter Keller, Erik C. Böttger, Petros C. Karakousis, Anne J. Lenaerts, James C. Sacchettini

**Affiliations:** 1 Department of Biochemistry and Biophysics, Texas A&M University, College Station, TX, USA; 2 Mycobacteria Research Laboratories, Department of Microbiology, Immunology and Pathology, Colorado State University, Fort Collins, CO, USA; 3 Center for Tuberculosis Research, Department of Medicine, Johns Hopkins University School of Medicine, Baltimore, MD, USA; 4 Department of Immunology and Infectious Disease, Harvard T. H. Chan School of Public Health, Boston, MA, USA; 5 Structure-guided Drug Discovery Coalition, SGC Toronto, ON, Canada; 6 Drug Discovery Unit, Division of Biological Chemistry and Drug Discovery, College of Life Sciences, University of Dundee, Dundee, UK; 7 Department of Computer Science and Engineering, Texas A&M University, College Station, TX, USA; 8 NRF Centre of Excellence for Biomedical TB Research and the South African MRC Centre for Molecular and Cellular Biology, Division of Molecular Biology and Human Genetics, Stellenbosch University, Tygerberg, South Africa; 9 Institute of Medical Microbiology, National Center for Mycobacteria, University of Zurich, Zurich, Switzerland

**Keywords:** polyketide synthase, Pks13 thioesterase domain, crystal structure, benzofuran inhibitors, structure-based drug discovery, Mycobacterium tuberculosis

## Abstract

Widespread resistance to first-line TB drugs is a major problem that will likely only be resolved through the development of new drugs with novel mechanisms of action. We have used structure-guided methods to develop a lead molecule that targets the thioesterase activity of polyketide synthase Pks13, an essential enzyme that forms mycolic acids, required for the cell wall of *Mycobacterium tuberculosis*. Our lead, TAM16, is a benzofuran class inhibitor of Pks13 with highly potent in vitro bactericidal activity against drug-susceptible and drug-resistant clinical isolates of *M. tuberculosis*. In multiple mouse models of TB infection, TAM16 showed in vivo efficacy equal to the first-line TB drug isoniazid, both as a monotherapy and in combination therapy with rifampicin. TAM16 has excellent pharmacological and safety profiles, and the frequency of resistance for TAM16 is ∼100-fold lower than INH, suggesting that it can be developed as a new antitubercular aimed at the acute infection.

**PaperClip:**

## Highlights

• Structure-guided medicinal chemistry used to develop potent and safe lead molecule
• First lead molecule reported that targets a polyketide synthase
• TAM16 is active against clinical drug-resistant *M. tuberculosis*
• Lower frequency of resistance than INH

## Graphical Abstract

**Figure:**
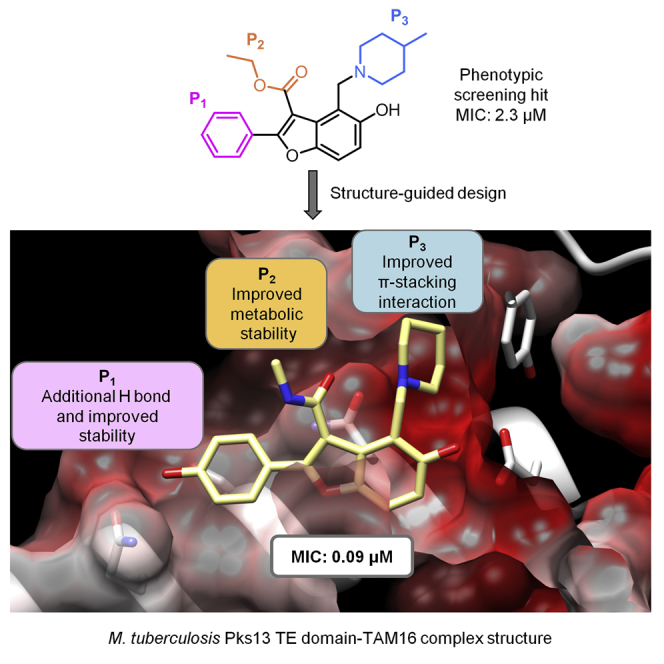

## Teaser

A small molecule inhibitor of *M. tuberculosis* polyketide synthase shows strong efficacy in murine models of infection.

## Introduction

Drug-resistance in *Mycobacterium tuberculosis* (*Mtb*) is a serious problem that threatens to worsen the global tuberculosis (TB) epidemic (World Health Organization, 2014). Although current six-month therapy for drug-susceptible TB can achieve a cure rate of >90%, the treatment of drug-resistant strains is more protracted (≥2 years) and involves the use of costly and less effective second-line drugs that have significant side effects (Zumla et al., 2013).

Isoniazid (INH) is a frontline TB drug that has been a mainstay of TB therapy since its introduction in 1952 (Bernstein et al., 1952). However, resistance occurs frequently with in vitro rates of about 1 in 10^−5^–10^−6^, which translates to high levels of clinical resistance ranging from 9.5% to 62%, based on geography and disease burden (Jenkins et al., 2011, World Health Organization, 2014). INH is a pro-drug that is activated by a catalase-peroxidase enzyme (KatG) to produce a radical that attacks nicotinamide adenine dinucleotide (NAD) to form a covalent adduct. This adduct inhibits the enoyl-ACP reductase, InhA (Rozwarski et al., 1998), an enzyme required for the synthesis of very long chain fatty acids that are used to form mycolic acids (Vilchèze et al., 2000). Because INH is activated by the non-essential KatG, resistance to INH often arises through loss-of-function mutations in the *katG* gene (Heym et al., 1995, Zhang et al., 1992). Indeed, the most common cause of INH resistance is the loss-of-function mutation KatG-S315T, which has been found in as many as 94% of INH-resistant and up to 82% of multidrug-resistant (MDR) *Mtb* clinical isolates (Torres et al., 2015). In addition, mutations in the *inhA* gene and its promoter region, i.e., the c-15t base change, further complicate the treatment of drug-resistant TB, conferring resistance not only to INH but also to the second-line TB drug ethionamide (ETH) (Banerjee et al., 1994), with reported frequencies of 35% and 55% in INH- and ETH-resistant clinical isolates, respectively (Vilcheze and Jacobs, 2014). After 65 years of use, the widespread and very high levels of INH resistance underscore the urgent clinical need for the development of alternative cell wall-active antibiotics for TB.

Mycolic acids are critical for viability and virulence of *Mtb*. Though these long-chain (C_60–90_) α-branched-β-hydroxylated fatty acids are primarily found esterified to the arabinogalactan-peptidoglycan cell wall core, they are also present as trehalose monomycolate and dimycolate esters in the cell envelope (Barry et al., 1998). In *Mtb*, mycolic acid biosynthesis occurs through the concerted action of more than 20 enzymes that are components of different multi-enzyme complexes (Takayama et al., 2005). Therefore, this pathway represents an important reservoir of novel targets for the development of new TB drugs, especially in the context of the emergence of drug resistance.

Polyketide synthases (PKS) are an important family of enzymes that have not been exploited as drug targets for any microbial pathogen. The *Mtb* H37Rv genome has about 24 PKS encoding genes (Cole et al., 1998). Genetic and biochemical studies have now linked most of the mycobacterial PKSs to participating in complex lipid biosynthestic pathways in *Mtb* (Chopra and Gokhale, 2009, Quadri, 2014). These PKS-derived lipid metabolites form essential components of the uniquely lipid-rich and complex cell wall of *Mtb*, which has been proposed as a means for it to survive under harsh conditions in host macrophages while also imparting an intrinsic resistance against many anti-microbial agents (Bhatt et al., 2007, Glickman et al., 2000).

**Figure 1:**
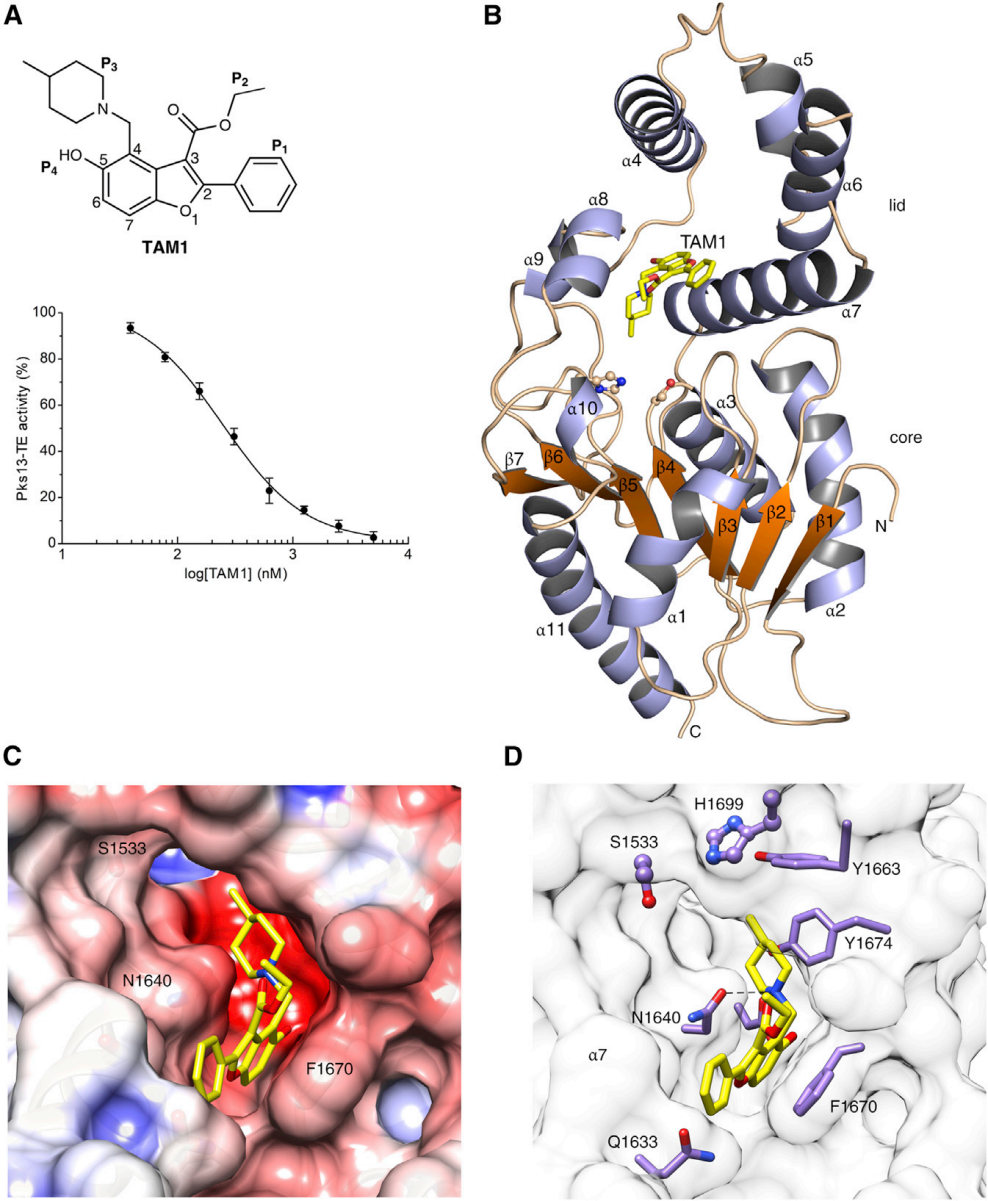
Novel Benzofurans Inhibit Pks13 Thioesetrase Domain (A) Chemical structure of TAM1 highlighting the convention used for naming the substituent groups (P_1_, P_2_, P_3_, and P_4_) and numbering of the benzofuran ring. TAM1 inhibits the esterase activity of Pks13-TE with an IC_50_ = 0.26 ± 0.03 μM. The graph depicts percent activity relative to DMSO only control (mean ± SD). (B) Overall view of the structure of the Pks13-TE-TAM1 complex showing structural features of the Pks13-TE domain. Catalytic residues His1699 and Ser1533 at the interface of the lid and core domains are shown as ball and sticks. TAM1 is shown as yellow sticks. (C and D) Close-up views of inhibitor interactions show that benzofuran core of TAM1 (yellow sticks) wedges between Phe1670 and Asn1640 with its P_3_ group oriented toward the catalytic site. Hydrogen bonds are represented by dashed lines. Surface representation in (C) is colored by electrostatic potential (contoured at ± 5 kT/e, red for negative and blue for positive). See also Figure S1 and Tables S1, S2, and S3.

In *Mtb*, Pks13 performs the final assembly step of mycolic acid synthesis, i.e., the Claisen-type condensation of a C_26_ α-alkyl branch and C_40–60_ meromycolate precursors (Portevin et al., 2004). It is comprised of five domains, including two acyl carrier protein domains, a β-ketoacyl-synthase, an acyltransferase, and a C-terminal thioesterase (TE) domain, that together contain all of the activities required for the condensation of two long-chain fatty acids. This activity has been shown to be essential both in vitro and in vivo (Portevin et al., 2004, Wilson et al., 2013). We recently discovered a small molecule that was active against *Mtb* H37Rv (TAM1; Figure 1A) and identified that Pks13 was the target through whole-genome sequencing and recombineering of the resistance mutations (Ioerger et al., 2013). In another study, a series of thiophenes were identified that kill *Mtb* by targeting the N-terminal ACP_N_ domain of Pks13. Wilson et al., 2013, propose that the compounds function by blocking the interaction of ACP_N_ with FadD32 protein, which transfers the meromycolyl chain. These results substantiate Pks13 as a druggable target for *Mtb* and highlight its potential for the development of new TB drugs that interfere with the critical pathway of mycolic acid synthesis.

In this paper, we describe the structure-based development of a highly potent and very safe lead compound, TAM16 (Table 1), which targets Pks13. It is active against MDR and extensively drug-resistant (XDR) *Mtb* clinical strains in vitro, demonstrating a lack of cross-resistance with existing TB therapeutics. By inhibiting cell wall biosynthesis, it synergizes with other TB drugs, like rifampicin (RIF), likely by augmenting their penetration into *Mtb*. Importantly, in murine TB infection models, it demonstrates efficacy equal to INH. Furthermore, unlike INH, which shows a relative high frequency of resistance, TAM16 shows 100-fold lower frequency of resistance. These properties, combined with the excellent pharmacokinetic (PK) and toxicity profiles, will likely allow us to convert this lead into a first-line drug.

**Table 1:** Preliminary SAR of TAM1 and Its Analogs

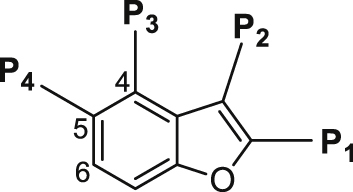						
Compound	P_1_	P_2_	P_3_	P_4_	IC_50_ (μM)∗	MIC (μM)
TAM1				OH	0.26 ± 0.03	2.3
TAM2				OH	0.12 ± 0.02	4.4
TAM3				OH	0.24 ± 0.02	4.1
TAM4				OH	0.28 ± 0.03	4.6
TAM5				OH	0.71 ± 0.05	13.3
TAM6				OH	1.57 ± 0.15	7.3
TAM7			None	OH	20 ± 1.9	20
TAM8				OH	11.9 ± 2.3	>40
TAM9				OH	0.26 ± 0.04	0.4
TAM10				OH	6.6 ± 0.7	NI
TAM11				OH	19.6 ± 1.4	5.2
TAM12				OH	0.29 ± 0.01	0.2
TAM13				OH	0.17 ± 0.02	1.2
TAM14				MeO	35.8 ± 2.2	ND
TAM15				H	2.0 ± 0.1	16
TAM16				OH	0.19 ± 0.01	0.09
TAM17				OH	0.36 ± 0.04	4
TAM18				OH	0.33 ± 0.03	0.5
TAM19				H, C6-OH	0.57 ± 0.03	1
TAM20				H, C6-OH	0.45 ± 0.04	1.1
TAM21				OH	0.42 ± 0.04	10
TAM22				OH	>10	>10
TAM23				OH	0.38 ± 0.01	2.5
TAM24				OH	2.1 ± 0.3	0.5

IC_50_ values were determined using the *Mtb* Pks13-TE domain as described in the methods section. MIC values were determined for *Mtb mc*^*2*^*7000* in liquid medium in 96-well plates. MeO, methoxy; NI, no inhibition; ND, not determined.

∗ Values are shown as mean ± SD of three independent measurements.

## Results

### TAM1 Inhibits Pks13 TE Domain Activity

Two laboratory-derived mutant strains resistant to TAM1 were found to harbor non-synonymous mutations, i.e., either D1607N or D1644G, both located in the TE domain of Pks13. To characterize the precise mechanism of action of TAM1 on the TE activity, a recombinant-expression plasmid was constructed to produce the domain for biochemical analysis. The pure recombinant protein, consisting of the TE domain of the Pks13 (Pks13-TE), was enzymatically active and produced diffraction-quality crystals complexed to TAM1.

An enzyme assay was developed for the TE activity of Pks13 using the fluorescent fatty acid ester, 4-methylumbelliferyl heptanoate (4-MUH) (Richardson and Smith, 2007). Pks13-TE was able to cleave the ester of 4-MUH, and kinetic analysis indicated a Michaelis constant (K_m_) ∼20 μM and *k*_*cat*_/*K*_*m*_ *∼* 7.2 × 10^2^ M^−1^ min^−1^ (Table S1). TAM1 inhibited the Pks13-TE activity with a half-maximal inhibitory concentration (IC_50_) of 0.26 μM (Figure 1A; Table S1).

### TAM1 Blocks the Active Site of Pks13-TE

As a first step to structure-guided medicinal chemistry on the benzofuran inhibitor, we solved the crystal structure of Pks13-TE complexed with TAM1 and refined it to high resolution (2.0 Å; Table S2). The crystals contained two monomers in the crystallographic asymmetric unit (designated A and B). The overall structure of Pks13-TE consists of a core domain and a lid domain (Figure 1B). The larger core possesses a canonical α/β-hydrolase fold comprised of a central seven-stranded β sheet (β1–β7) flanked by four α helices (α1–α3 and α11) with the N-terminal β1 strand anti-parallel to other β strands (Nardini and Dijkstra, 1999). The lid domain (residues 1575–1645) is inserted between strands β5 and β6 and consists of four α helices, α4–α7, along with two short helices, α8 and α9, (residues 1665–1675) present between the strands β6 and β7 of the core domain. Based on the analysis with VAST server (Gibrat et al., 1996), the Pks13-TE lid domain appears to be relatively unique among TE structures reported to date.

**Figure S1:**
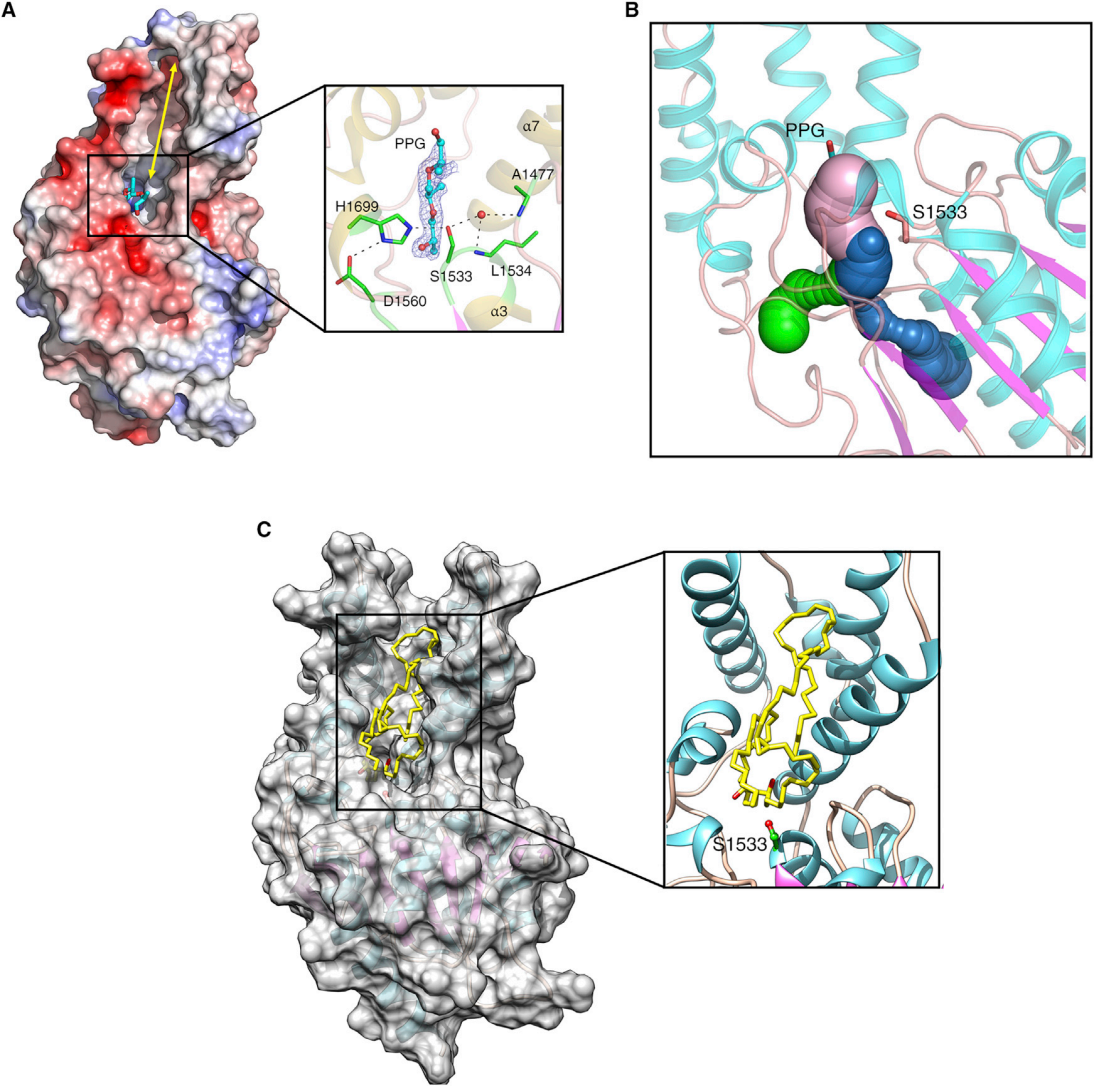
Structural Features of the Pks13-TE Crystal Structure, Related to Figure 1 (A) Surface representation of the Pks13-TE structure colored by electrostatic potential (contoured at ± 5 kT/e; red for negative and blue for positive) to illustrate the substrate binding groove (∼30 Å, double-headed yellow arrow) on the lid domain. The zoomed view shows the bound PPG fragment (cyan) in the catalytic pocked formed by residues Ser1533, His1699 and Asp1560 along with the residues of the oxyanion-hole (Leu1534 and Ala1477) rendered as sticks; catalytic water shown as red sphere. The 2Fo-Fc electron density map contoured at 1.2σ is shown for the bound PPG fragment. Hydrogen bond interactions are shown as black dashed lines. (B) Predicted tunnels in Pks13-TE structure by CAVER analysis (Chovancova et al., 2012). The three potential tunnels are shown in pink, blue and green surface rendering. The largest of the tunnels (pink) opens onto the substrate binding surface groove and contained the bound PPG fragment. (C) Docking of mycolic acid on Pks13-TE lid domain. A molecule of mycolic acid (shown as yellow sticks) was docked using Molsoft ICM-Pro software to determine a possible binding mode in the substrate binding groove. The zoomed view of the docking indicates that the surface groove can accommodate acyl chains of the mycolic acid precursor attached to the C-terminal ACP domain and position the thioester for cleavage near the catalytic Ser1533 residue.

The Pks13-TE active-site pocket is formed at the interface between the lid and core domains. The catalytic triad was identified to be Ser1533, Asp1560, and His1699, and the oxyanion hole is formed by the amide N-atoms of Leu1534 and Ala1477. Extending from the active site is a deep (∼30 Å) hydrophobic groove that spans the full length of the lid domain, with a total surface area of ∼1290 Å^2^ (Figure S1A). A similarly located surface groove (∼20 Å) in the α-helical lid domain of bovine palmitoyl-protein thioesterase 1 (PPT1, 15% identity with Pks13-TE), contained the substrate palmitic acid (Bellizzi et al., 2000). We observed unexpected electron density in this pocket of apo-Pks13-TE structure that could be built as an eight-carbon fragment of polypropylene glycol (PPG, C_8_O_5_), an additive in the crystallization buffer. The fragment is located in the fatty acyl chain-binding site based on the superimposition of Pks13-TE structure with the bovine PPT1 and human FAS TE (hFAS-TE) structures (Zhang et al., 2011). However, unlike the Pks13-TE acyl chain-binding site, the bovine PPT1 and hFAS-TE is not surface exposed. The PPG binding pocket is connected at the catalytic site to a series of tunnel-like regions that could also bind an acyl chain (Figure S1B). The more elaborate system of binding grooves and tunnels is likely related to the very long carbon chains (C80–90) that make up the mycolic acid precursor, compared to PPT1 and hFAS (Figure S1C).

**Figure S2:**
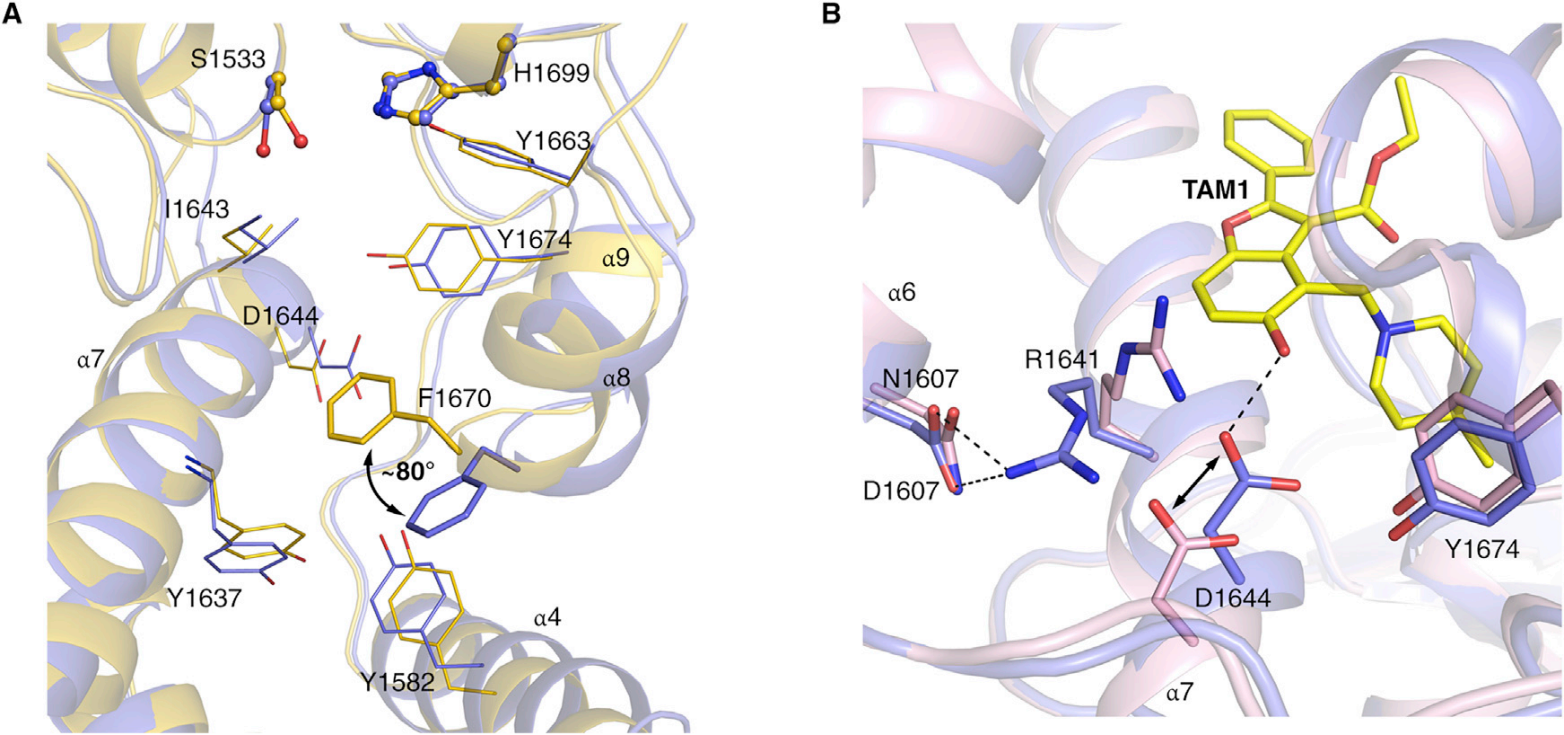
Structural Changes in TAM1-Bound and D1607N-Mutant Pks13-TE Crystal Structures, Related to Figure 1 (A) Superimposition of Pks13-TE-TAM1 complex structure (purple) with Apo-Pks13-TE structure (yellow) shows that Phe1670 side chain (shown as stick) flips by ∼80° upon TAM1 binding. TAM1 interacting residues are shown in line representation in purple color, and the corresponding residues from Apo structure are shown as lines in yellow color. Catalytic residues (Ser1533 and His1699) are shown as ball and sticks. TAM1 is omitted for clarity of presentation. (B) Superimposition of Pks13-TE-TAM1 complex structure (purple, TAM1 as yellow sticks) with the structure of D1607N mutant (pink) shows the conformational change in Arg1641 of the mutant due to disruption of ion pair interaction with Asp1607. In the wt Pks13-TE structure, Asp1607 carboxylate forms an ion pair interaction with the guanidinium of Arg1641 which helps stabilize the C-terminal end of helix α7. This allows Asp1644 to form hydrogen bond interaction (shown as dashed black line) with TAM1. The mutation D1607N breaks the ion pair interaction mediated anchoring of helix α7 that causes Asp1644 to move away by ∼3 Å (double-headed black arrow), consequently disrupting its interaction with TAM1.

TAM1 binds in the fatty acyl chain-binding groove at the entrance of the Pks13-TE active site, effectively blocking access of the substrate to the catalytic center of the enzyme (Figures 1B–1D). Several differences were seen between the apo- and TAM1-bound structures of Pks13-TE, the most significant being in the side chain of Phe1670. Phe1670 is located at the end of helix α8 directly adjacent to the acyl binding pocket. The phenyl ring of Phe1670 flips by about 80° in the TAM1 structure, compared to the apo protein, to form a slightly off-plane van der Waals stacking interaction with the furan ring (Figure S2A). The four different substituents attached to the benzofuran scaffold (P_1_, P_2_, P_3_, and P_4_; Figure 1A) were found to interact with residues that line the substrate-binding cleft. Indeed, most of the binding interactions occur between amino acids from helix α7 of the lid domain and the two supporting helices α8–α9 along with the loop that connects them to strand β6 of the core domain (Figures 1B and 1D). Overall, the structure showed that the phenyl group of TAM1 (P_1_) is solvent exposed; P_2_ ethyl ester is partially solvent exposed, while P_3_ piperidine and P_4_ OH are completely buried in Pks13-TE.

The Pks13-TE structure shows that the D1644G mutation confers resistance through the loss of a direct H bond between a carboxylate oxygen and the P_4_ OH of TAM1. The crystal structure of the D1607N mutant (Figure S2B; Table S2) explained its more subtle effect on binding, through the loss of ion pair interaction between the carboxylate of Asp1607 on helix α6 and the guanidinium of Arg1641 (3 Å) located on the parallel helix, α7. Without this interaction, α7 shifts away from the substrate-binding groove by about 3 Å, moving Asp1644 out of hydrogen-bonding distance from the P_4_ OH of TAM1.

### Structure-Based Development of TAM1 Analogs

**Figure 2:**
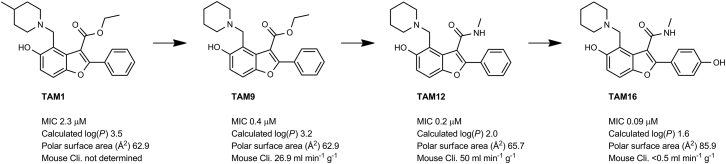
Structure-Guided Development of TAM16 Substitution of P_1_ and P_2_ groups in TAM16 with phenol and methyl amide, respectively, increased potency and metabolic stability. Calculated log(*P*), calculated log(partition coefficient); Mouse Cli., intrinsic clearance in mouse liver microsomes.

**Figure S3:**
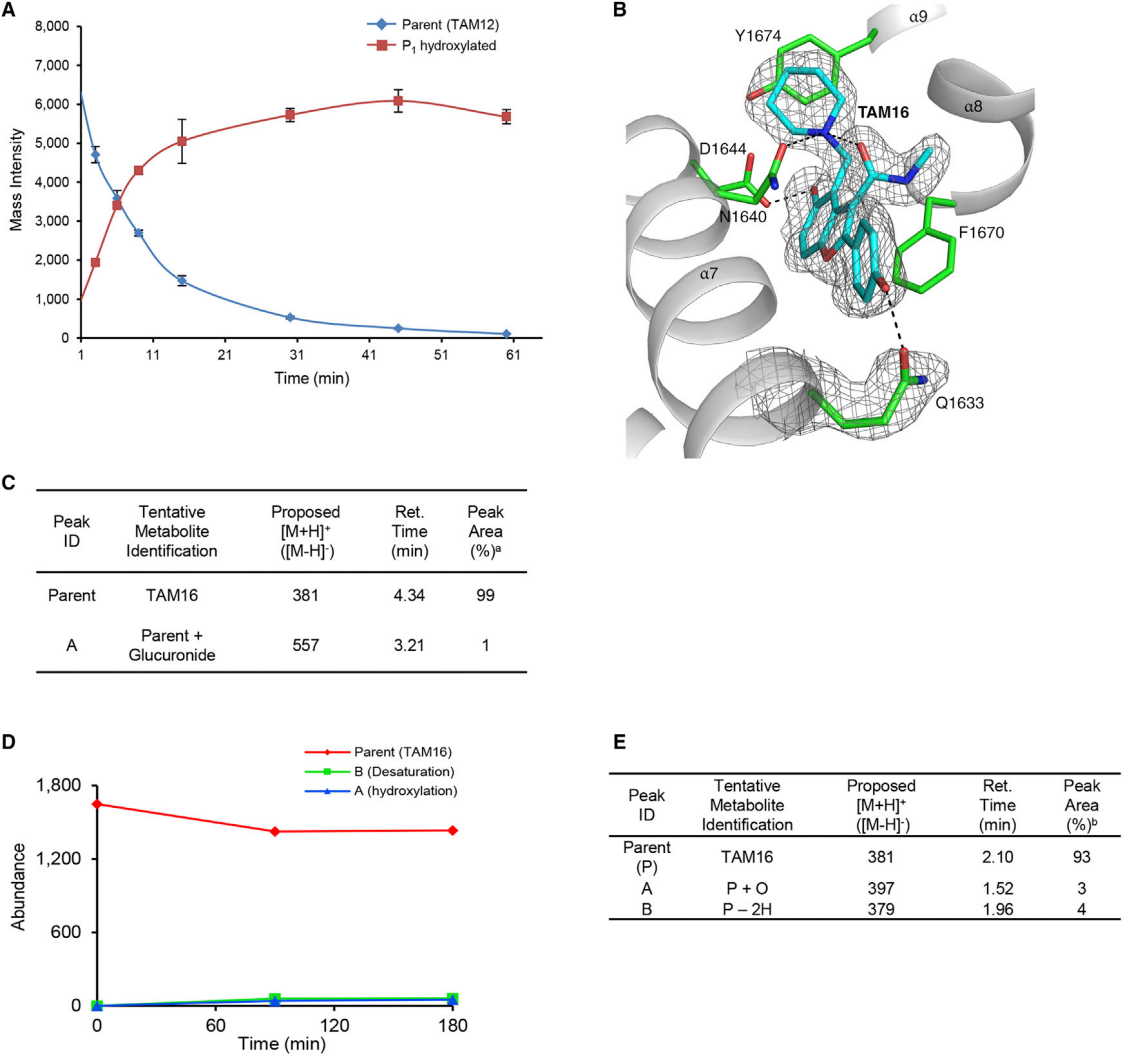
Metabolic Stability of TAM1 Analogs, Related to Table 3 (A) Metabolic stability studies of TAM12 in mouse liver microsomes showed that TAM12 was hydroxylated at P1 phenyl. Graph represents mean values ± SD of two independent assays. (B) Cartoon of the Pks13-TE-TAM16 complex structure showing hydrogen bond interaction between P_1_ 4-OH of TAM16 (cyan) and the side chain carbonyl oxygen atom of Gln1633 (green). The gray mesh represents the 2*mF*_o_ - *DF*_c_ maximum-likelihood omit map, contoured at 1.2σ. Hydrogen bonds are shown as black dashed lines. (C) Glucuronidation of TAM16 was assessed in mouse liver microsomes with UDPGA (5 μM for 60 min). At 0, 30 and 60 min, 100 μL aliquots of the reaction mixture were removed and placed in 100 μL of acetonitrile to terminate the reaction. Analysis by mass spectrometry for metabolite identification showed little metabolism of the parent compound (TAM16), the conjugate was barely detectable after 60 min incubation (peak A). (D and E) Metabolic stability analysis of TAM16 incubated with glutathione and methoxylamine (50 μM for 180 min) in the presence of mouse plasma and HLMs for possible retro-Mannich metabolites or adducts. The parent compound was seen to decrease slightly over time and two metabolites were detected. For metabolite B the main ion see was at m/z 379 but a barely detectable ion was seen at m/z 397 indicating the metabolite may be due to oxidation which dehydrates readily in the MS. No trace of the quinone-methide or potential GSH adducts was seen in any of the samples. ^a^% of total peak area for parent and metabolite peaks of the proposed [M+H]^+^ ions at 60 min time point. ^b^% of total peak area for parent and metabolite peaks of the proposed [M+H]^+^ ions at 180 min time point.

TAM1 was modified using structure-guided approaches to improve the potency and pharmacological properties (Table 1; Figure 2; STAR Methods; Table S3). Briefly, the two major pharmacological liabilities of TAM1were the phenyl at P_1_, which was hydroxylated in mouse liver microsomes (MLMs) to yield primarily the 4-OH (Figure S3A), and the P_2_ ester, which was cleaved to the inactive acid in mouse serum. The structure of the Pks13-TE-TAM1 complex indicated that the side chain amide of Gln1633 was positioned in close proximity (∼4 Å) to the *para*-position of the P_1_ phenyl ring, suggesting that 4-OH form of the inhibitor would retain enzyme inhibitory activity. Indeed, the P_1_ 4-OH-containing analog, TAM16, had greatly improved metabolic stability (Figure S3), showed significant improvement in enzyme potency, and exhibited >20-fold improvement in *Mtb* potency compared with TAM1 (Table 1). The structure also indicated that the bioisosteric replacement of the ester with a methyl amide that would be stable in serum would be tolerated in the binding pocket. Indeed, the methyl amide of TAM12 was stable and showed similar enzyme-inhibitory activity (IC_50_ 0.3 μM) as the ethyl ester analog TAM9; however, it exhibited 2-fold better whole-cell activity (Table 1). Among P_3_ groups, the piperidine was the most potent of the P_3_ substituents synthesized. Structures of P_3_ substituted analogs showed that five-, six-, and seven-membered rings at P_3_ led to variations in the van der Waals and stacking interactions with the planar side chain of Tyr1674, and these interactions were abolished in TAM6 with acyclic dimethyl amine at P_3_. Thus, among the P_3_ analogs, the piperidine group was optimally located sandwiched between Tyr1663 and Tyr1674, and its protonated N appears to be a bifurcated hydrogen donor, forming an intra-molecular hydrogen bond with the P_2_ carbonyl oxygen (2.9 Å) and another with the side chain oxygen of Asn1640 (2.9 Å). We also explored alterations to the P_4_ OH group. All P_4_ analogs at C-5 showed a dramatic loss in enzyme potency presumably due to loss of hydrogen bond with the carboxylate of Asp1644. The resulting molecule, TAM16, was the most potent and stable inhibitor (IC_50_ 0.19 μM, minimum inhibitory concentration [MIC] 0.09 μM) (Figures 2 and S3B).

### Low Frequency of Resistance, No Cross-Resistance, and Combination Potential of TAM16

Resistant mutants emerged at frequencies of 7 × 10^−7^ and ∼8 × 10^−8^ at 10× and 20× MIC, which were ∼100-fold lower than the frequency observed for INH (1 × 10^−6^). Sequencing of two resistant mutants revealed point mutations in *pks13* that resulted in a single non-synonymous amino acid substitution in Pks13 (D1644G or D1644Y), and they retained sensitivity to INH and RIF (MICs 0.2 and 0.04 μM, respectively).

We determined MIC values of TAM16 against 38 clinical *Mtb* strains representing a wide range of mutations covering all known molecular mechanisms involved in imparting resistance against first- and second-line TB drugs that form the core component of TB treatment regimen (Tables S4 and S5). TAM16 was highly active against pan-susceptible clinical isolates of *Mtb* (MICs 0.06–0.250 μM). Most importantly, TAM16 was potent against all MDR and XDR clinical isolates of *Mtb* evaluated (MIC range of 0.05–0.42 μM; Table 2). The lack of cross-resistance of TAM16 with current TB drugs shows the importance of developing new drugs with distinct modes of action.

**Table 2:** TAM16 MIC Values for *Mtb* Strains with Different Drug-Susceptibility Profiles, Related to Tables S4 and S5

Strain and resistance status^a^	Number of strains	MIC_90_^b^ range for multiple strains (μM)	Median MIC_90_ (μM)
H37Rv, fully susceptible lab strain	1	-	0.125-0.25
H37RvMa, fully susceptible lab strain	1	-	0.125
*Mtb*, fully susceptible clinical isolates	12	0.060-0.250	0.100
*Mtb*, poly-resistant clinical isolate	1	-	0.420
*Mtb*, MDR clinical isolates	7	0.060-0.210	0.210
*Mtb*, pre-XDR clinical isolates	5	0.125-0.420	0.420
*Mtb*, XDR clinical isolates	5	0.125-0.250	0.125
*Mtb*, INH mono-resistant, clinical	6	0.050-0.125	0.100
*Mtb*, RIF mono-resistant, clinical	1	-	0.125
*Mtb*, SM mono-resistant, clinical	1	-	0.420

Poly-resistant, *Mtb*-strain resistant to isoniazid (INH), ethionamide, and streptomycin (SM); MDR, resistant to both INH and rifampicin (RIF), with or without resistance to other anti-TB drugs; pre-XDR, MDR strains with additional resistance to either a fluoroquinolone or an injectable but not both; XDR, MDR strains that are also resistant to any fluoroquinolone and to any of the three second-line injectables (amikacin, capreomycin, and kanamycin).

a Detailed description of the strains with their drug-resistance phenotypes is given in Tables S4 and S5.

b The lowest concentration of drug that inhibited growth of more than 90% of the bacterial population was considered to be the MIC_90_. The MIC_90_ values were determined using MGIT 960 system.

One of the advantages of drugs that target cell wall biosynthesis, like INH, is their ability to combine with other antibiotics to increase efficacy. We tested TAM16 in combination with INH, RIF, and EMB in vitro in two-drug combination studies by the combination index (CI) method (Chou, 2006) (wherein CI < 1, CI = 1, and CI > 1 indicate synergistic, additive, and antagonistic interactions, respectively). While TAM16 showed no synergistic activity with INH and EMB, the two other drugs that target cell wall, the combination of TAM16 with RIF, which targets the RNA polymerase, showed highly synergistic activity (CI = 0.55) against *Mtb mc*^*2*^*7000*. Notably, at the combination ratio of 1:0.5, the MIC of RIF was improved ∼3-fold to 0.011 μM compared to RIF alone (MIC 0.35 μM), while the MIC of TAM16 improved by 5-fold (0.022 μM) compared to TAM16 alone (0.10 μM). This synergy is likely based on increased permeability of the bacterial cell wall leading to enhanced RIF accumulation. Indeed, two-drug combinations of TAM16 with antitubercular drugs TMC207 (a diarylquinoline compound that inhibits ATP synthesis) and streptomycin (SM, an aminoglycoside antibiotic that inhibits protein synthesis) also showed synergistic activity (CI ∼0.54 and 0.81, respectively). The combination of TAM16 and TMC207 (at a combination ratio of 1:0.5) improved the MIC of TAM16 in the presence of TMC207 by ∼6.5-fold to 0.016 μM, and the MIC of TMC207 in the presence of TAM16 was improved 2.6-fold to 0.005 μM compared to the MIC of TMC207 alone (MIC 0.013 μM). Similarly, the combination of TAM16 and SM (at a combination ratio of 1:2) was synergistic: it improved TAM16 MIC by ∼4-fold in the presence of SM, and the addition of TAM16 improved the MIC of SM ∼2-fold to 0.037 μM compared to the SM MIC when it was used alone (0.07 μM). Overall, these results are consistent with previously observed effects of cell wall inhibitors on improving the penetration of anti-*Mtb* drugs (Bhusal et al., 2005, Bosne-David et al., 2000, Lechartier et al., 2012).

### Toxicology and Pharmacokinetic Properties of TAM16

TAM16 has excellent physicochemical, toxicological, and pharmacological properties (Table 3). It showed low plasma protein binding in both mouse and human plasma and exhibited very low clearance in MLM and HLMs (human liver microsomes) (CL_int_ < 0.5 mL/min/g liver; Table 3), and the two phenolic OH groups were not significantly glucuronidated in the microsomal incubation assays (Figure S3C).

**Table 3:** Physicochemical and Pharmacokinetic Properties of TAM16

MW (g/mol)		380.4
cLogP		1.6
logD		1.7
Lipophilic ligand efficiency		5.1
H-bond donors		3
H-bond acceptors		4
TPSA (Å^2^)		86
pKa		9.95
Kinetic solubility (μM)		74
(phosphate buffer, pH 7.4)		
Plasma protein binding (%):	Mouse	73
	Human	72
Intrinsic clearance in liver microsomes		
(CL_int_) (mL/min/g liver):	Mouse	<0.5
	Human	<0.5
CYP inhibition		No significant inhibition
PK parameters:		
*C*_max_ (ng/mL) in plasma		444
*T*_*max*_ (hr)		0.5
*t*_1/2_ (hr) in plasma		1.0
AUC_0-24_ (ng.min/mL) (po)		74,940
AUC_0-24_ (ng.min/mL) (iv)		79,369
Clearance (mL/min per kg)		37
*V*_ss_ (L/kg)		4.2
Oral bioavailability (*F*) (%)		28

Pharmacokinetic parameters were determined after administration of single oral (po) and intravenous (iv) doses of TAM16 at 10 mg/kg and 3 mg/kg, respectively, in female BALB/c mouse. *C*_*max*_, maximum concentration; *T*_*max*_, time to reach *C*_*max*_; t_1/2_, half-life; AUC, area under the concentration curve; *V*_*ss*_, volume of distribution at steady state; cLogP, calculated log(partition coefficient); Lipophilic ligand efficiency = pIC_50_ − cLogP; TPSA, total polar surface area.

**Figure S4:**
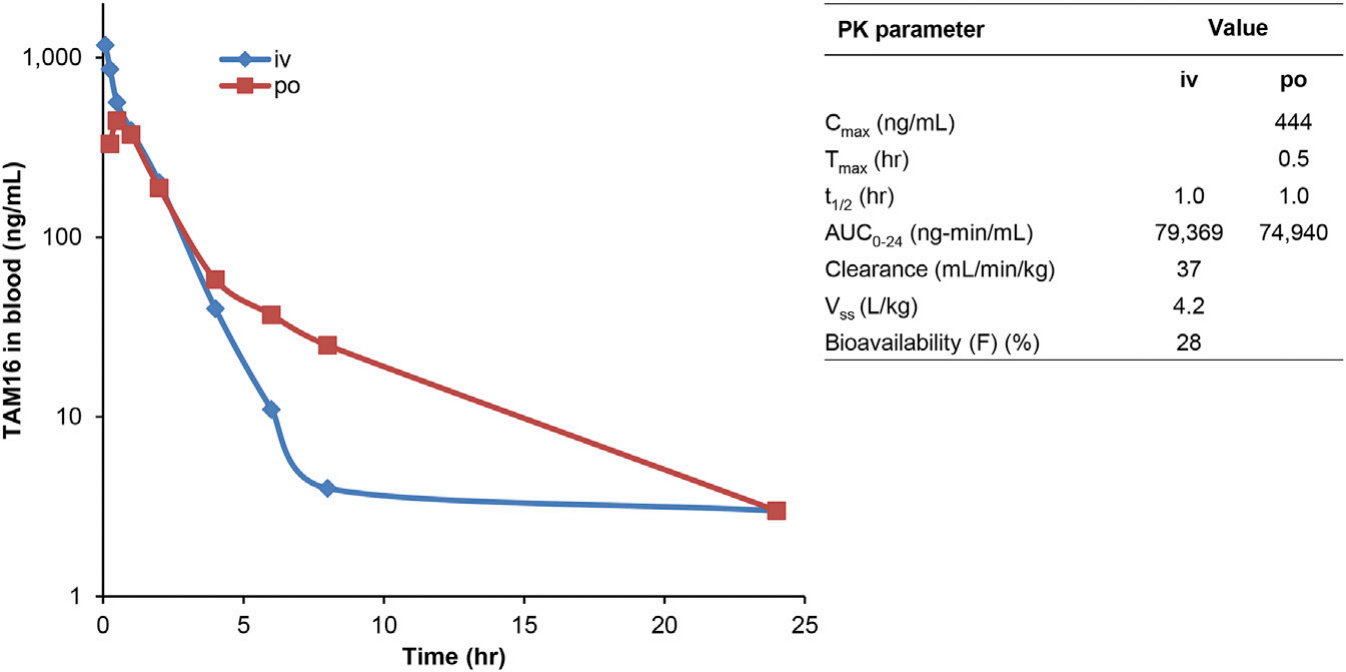
In Vivo Pharmacokinetics of TAM16, Related to Table 3 Mean blood concentration profiles of TAM16 following oral (p.o) and iv dose of 10 mg/kg and 3 mg/kg, respectively, in female BALB/c mouse. PK parameters (inset) were determined after the administration of a single dose (both p.o and iv) to mice. *C*_*max*_, maximum concentration; *T*_*max*_, time to reach *C*_*max*_, ${\text{t}}_{\left(1/2\right)}$, half-life; AUC, area under the concentration curve; *V*_*ss*_, volume of distribution at steady state.

See also Figures S3 and S4 and Table S6.

Pharmacokinetic studies in Swiss Webster mice were performed with TAM16 dosed by oral administration at 100 mg/kg twice daily. At this dose, maximum plasma concentration (C_*max*_) of TAM16 reached 3.6 μg/ml at 5 hr, and the AUC was 18.3 μg.hr/ml. At a single dose of TAM16, mouse exposure was higher than the MIC (34.3 ng/ml) for ∼12 hr. Pharmacokinetic parameters determined after a single oral and intravenous dose of 10 mg/kg and 3 mg/kg, respectively, in BALB/c mice showed that TAM16 had moderate total plasma clearance (37 mL/min per kg), a large volume of distribution (4.2 l/kg), good exposure, and oral bioavailability (28%) (Table 3; Figure S4).

TAM16 was not cytotoxic to mammalian cells at concentrations up to 100 μM, and it was well tolerated in BALB/c mice at up to 300 mg/kg administered orally once daily for 3 days. Furthermore, TAM16 had no observable inhibitory activity against most of the major CYP isoforms (Table S6). hERG inhibition activity of TAM16 was evaluated by thallium (Tl^+^) flux assay (Schmalhofer et al., 2010) displaying an IC_50_ of 21 μM. Additional off-target activity screening against a broad panel of therapeutically relevant enzymes and receptors did not reveal any significant issues related to human safety. Moreover, in a two-strain Ames fluctuation assay for genotoxicity, the compound did not exhibit mutagenic potential in the two strains with or without S9.

For an antitubercular compound to be effective, it is essential that it achieves high exposure in the infected tissue consisting of macrophages and other immune cells in the human host. Cellular uptake studies using the cell line THP-1 as an in vitro macrophage model (Stokes and Doxsee, 1999) showed that, on average, cells maintained high (>70× MIC) concentrations of TAM16 (≥6.5 μM when treated with 10 μM compound) for up to 24 hr, indicating that the compound is freely permeable into the macrophage cytosol and can achieve high exposure. Indeed, TAM16 showed good activity in *Mtb* infected THP-1 cells.

Although TAM16 contains a Mannich substructure (C5OH C4 benzylamine scaffold), no adducts of TAM16 were formed in mouse plasma and HLMs upon incubation with glutathione and methoxylamine (Johansson et al., 2009) (Figures S3D and S3E). Moreover, TAM16 was stable after 4 weeks of incubation in vitro in low-pH buffered solutions (pH 3–5) at room temperature. Thus, taken together, the stability data suggests that the Mannich substructure in TAM16 does not represent a significant liability, consistent with previously reported stability of the piperidine adducts of hydroxyindoles (Monti and Castillo, 1970).

### TAM16 Efficacy in Murine TB Infection Models

**Figure 3:**
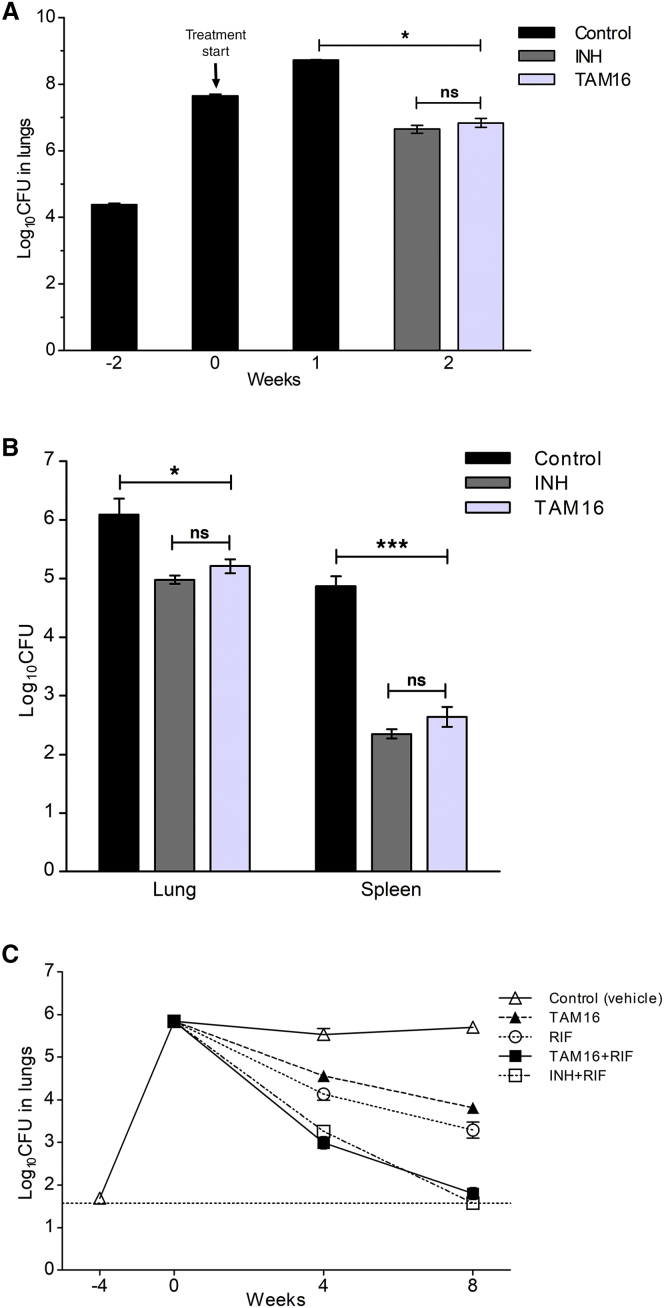
Efficacy of TAM16 in Mouse Models of TB (A) In vivo activity of TAM16 against acute TB infection in immunocompetent BALB/c mice. Data represent mean *M. tuberculosis* burden (log_10_ CFU) in the lungs of mice (n = 5 per time point) expressed as mean ± SD. Week 0 indicates CFU counts in the lungs at treatment initiation (2 weeks after infection). Drugs were administered via oral gavage 5 days/week for 2 weeks. The mice in the untreated group were moribund after 3 weeks of infection and were euthanized in accordance with institutional animal care regulations. ^∗^p < 0.05 by Dunnett’s multiple comparison tests, as compared to the untreated (vehicle-only) control group; ns, no statistical significance. (B) Efficacy of TAM16 in reducing *M. tuberculosis* burden in chronically infected immunocompetent mice after 4 weeks of treatment. Treatment was initiated 27 days after infection, and drugs were administered once daily via oral gavage for 5 days/week for 4 weeks. Data show the bacterial loads (mean log_10_ CFU ± SD) in the lungs and spleen of the infected mice (n = 5 per group). ^∗^p < 0.05 and ^∗∗∗^p < 0.001 by pairwise multiple comparison procedures (Tukey test); ns, no statistical significance. (C) Efficacy of TAM16 administered in combination with anti-TB drug rifampicin (RIF) in chronically infected BALB/c mice. Treatment was initiated 28 days after infection, and drugs were administered once daily via oral gavage (5 d/wk) for 4 and 8 weeks. Data represent mean *M. tuberculosis* burden (log_10_ CFU) in the lungs of the infected mice (n = 6 for vehicle-only control group and n = 7 for each treatment group) after 4 and 8 weeks of therapy (mean ± SEM). In combination studies, TAM16 and isoniazid (INH) were administered 1 hr following prior administration of RIF. Dotted horizontal line indicates the limit of detection. See also Figure S5 and Table S7.

**Figure S5:**
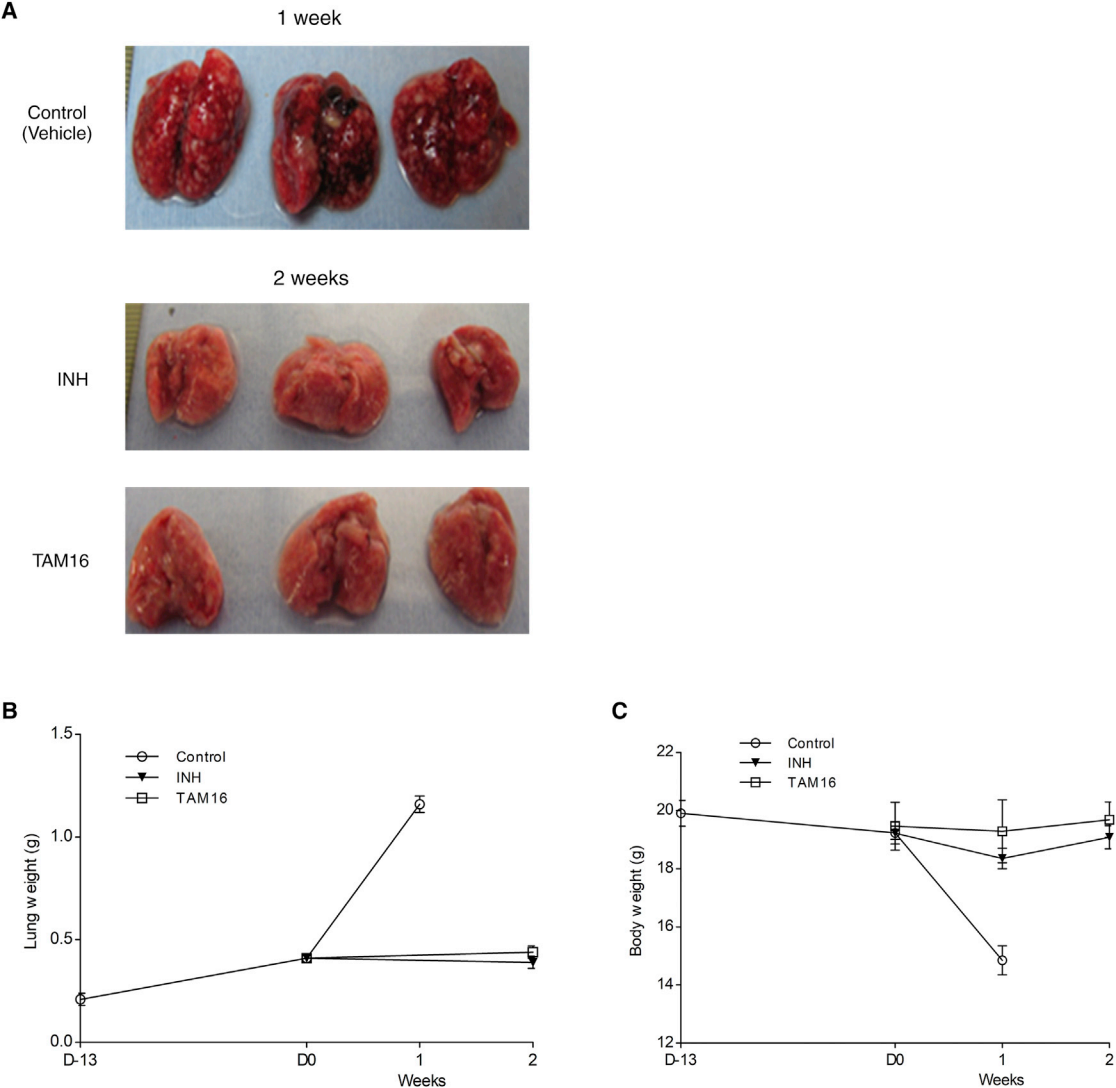
Effect of TAM16 Treatment on Mice in Acute BALB/c Model, Related to Figure 3A (A) Lung gross pathology images from untreated control (vehicle only) and treated mice. Untreated mice were moribund 1 week after treatment initiation (3 weeks post-infection) and were euthanized in accordance with institutional animal care regulations. INH, isoniazid. (B and C) Effect of drug treatment on mean lung weights and, (C) mean body weights in *M. tuberculosis* infected mice (n = 5 per time point per group) after 2 weeks of treatment. Mice were infected on Day −13 and treatment was initiated on D0 (2 weeks after infection). Graphs represent mean values ± SD.

We evaluated TAM16 for in vivo efficacy in mouse models of acute and chronic TB infection. The TAM16 dosing regimens used in these animal studies were based on the maximum tolerated dose and pharmacokinetic studies. We first tested the in vivo activity of TAM16 using a mouse model of acute TB (Dutta et al., 2014), representing a population of actively multiplying *Mtb* in the host. BALB/c mice lungs were implanted with a very high inoculum of *Mtb* (∼4.4 log_10_ bacilli), which multiplied to a peak lung burden of ∼7.7 log_10_ CFU (colony-forming unit) 14 days after infection. Treatment of the mice was initiated 2 weeks post-infection with once-daily oral dosing of TAM16 (200 mg/kg) or INH (10 mg/kg). After 2 weeks of treatment, the TAM16 treated group showed a significant reduction in the lung CFU to ∼6.8 log_10_, which was indistinguishable from INH treatment, which reduced the lung CFU counts to ∼6.7 log_10_ (p > 0.05). Contrarily, all of the untreated control mice were moribund 1 week after treatment initiation due to uncontrolled bacillary growth in the lungs and were euthanized in accordance with institutional animal care regulations (Figure 3A). Histopathology of the mice post-treatment showed that TAM16 prevented the development of characteristic lung lesions (Figure S5).

We next tested the in vivo activity of TAM16 in a chronic TB infection model in which lungs of BALB/c mice were aerosol-infected with *Mtb* using low-dose inocula, and treatment was initiated 4 weeks later when a steady-state infection was established at ∼7 log_10_ CFU per lung. After 4 weeks of treatment, mice treated with a once-daily oral dose of TAM16 (300 mg/kg) for 5 days per week showed a significant reduction in lung CFU counts by ∼0.9 log_10_ compared with the untreated control mice (p = 0.01) (Figure 3B). TAM16 treatment also significantly reduced the spleen bacterial burdens by ∼2.2 log_10_ CFU relative to that of the untreated control group (p < 0.001). Mice receiving INH (25 mg/kg) for 4 weeks showed a decrease in CFU counts by ∼1.1 log_10_ and ∼2.5 log_10_ in lungs and spleen, respectively, which again was statistically indistinguishable from the activity observed with TAM16 (p > 0.05) (Figure 3B).

Because of the synergy that was observed between TAM16 and RIF in the in vitro assays, we evaluated the drug combination of TAM16 and RIF in a mouse model representing a chronic TB infection. BALB/c mice were infected with a low-dose aerosol, and treatment was initiated 4 weeks post-infection with the combination of TAM16 (200 mg/kg) and RIF (10 mg/kg) in an 8-week study, where the combination was dosed orally once daily for 5 days per week. After 4 weeks of treatment, the combination of TAM16 and RIF resulted in a dramatic 2.54 log_10_ CFU reduction in the bacterial load in the mice lungs when compared to that of the vehicle-only control (p < 0.001) (Figure 3C). We saw a further reduction in bacterial load when the combination was continued for an additional 4 weeks with an impressive overall reduction of ∼3.9 log_10_ CFU in lungs over 8 weeks treatment when compared to the untreated control (p < 0.001). This was similar to the gold standard combination of INH and RIF that showed an efficacy of ∼4.1 log_10_ CFU reduction (p > 0.05) (Figure 3C; Table S7). Moreover, the combination of TAM16 and RIF was significantly more active than RIF alone, with an additional decrease of ∼1.5 log_10_ CFU counts after 8 weeks of treatment (p < 0.001). TAM16 as a single agent showed efficacy similar to RIF alone (CFU reduction of ∼1.9 log_10_ and ∼2.4 log_10_ in mice lungs, respectively) after 8 weeks of treatment (p > 0.05). In this study, no overt toxicity was observed in treated mice. Companion PK studies revealed a mean maximal drug concentration in plasma (C_*max*_ ± SD) of 6.21 ± 1.5 μg/mL and 6.30 ± 2.3 μg/mL with trough concentrations of 1.91 ± 1.1 μg/mL and 1.58 ± 0.8 μg/mL for TAM16 30 min and 24 hr following oral administration alone, or 1 hr following prior RIF administration when sampled at steady state (week 8 of dosing), respectively. Thus, taken together, the efficacy data indicates that TAM16 has potent in vivo activity equivalent to INH, which is the most bactericidal first-line TB drug in current use, and highlights its potential for further pre-clinical development.

## Discussion

INH, an inhibitor of the mycolic acid synthesis, has been a mainstay of TB therapy for decades. Although, in general, INH is well tolerated, it has several limitations. Because it is a pro-drug whose activation requires an *Mtb* enzyme that is not essential, resistance arises frequently through loss-of-function mutations in the activator KatG. Thus, a compound that targets mycolic acid synthesis and is not a pro-drug, such as the Pks13-targeting TAM16 developed in this study, should prove to be a better alternative to INH. Indeed, the frequency of resistance is about 100-fold lower for TAM16 as compared to INH. Though several other such compounds, such as InhA inhibitors, that do not require activation have been described (Guardia et al., 2016, Manjunatha et al., 2015, Pan and Tonge, 2012), none have advanced to the point where they can easily be compared with INH in animal studies, unlike TAM16.

TAM16 is a novel benzofuran class lead molecule with excellent drug-like properties and favorable pharmacokinetic and safety profiles. It exhibits potent in vivo efficacy in both acute and chronic mouse TB models when administered orally as a single dose, which is a highly desirable feature for a new TB therapeutic as it simplifies dosing regimen, encouraging patient compliance. However, the primary advantages of TAM16 are the novel target and the fact that it is not a pro-drug. This unique mode of action means that there will be no pre-existing resistance in clinical strains. Our studies verify that TAM16 is potent against multidrug-resistant and extensively drug-resistant *Mtb* strains.

One of the advantages of drugs that target mycobacterial cell wall biosynthesis is their potential to combine with other antibiotics to increase efficacy by improving their penetration. This makes compounds such as TAM16, which probably selectively kills the most rapidly growing bacteria in the population, producing rather rapid clearance initially with a more modest effect later in infection, very useful parts of a combination drug regimen. TAM16 combines well with RIF, a drug that kills different subpopulations of bacteria, suggesting that it is also likely to synergize with other drugs that have different modes of action. Indeed, TAM16 also showed synergistic activity in combination with other TB drugs like ATP synthesis inhibitor TMC207 and protein synthesis inhibitor streptomycin. Thus, TAM16 has suitable attributes for inclusion into either the current or any newly developed combination therapy for TB treatment. Overall, the results presented here demonstrate that TAM16 represents a promising candidate as a replacement of INH and validate Pks13 as a drug target in *Mtb*.

Structure-guided drug development has proven to be a powerful approach to producing new agents but has had limited application in the process of developing new antimycobacterial compounds (Lechartier et al., 2014, Zuniga et al., 2015). These findings re-emphasize the utility of structure-guided approaches for antimycobacterials when starting with compounds that have good whole cell and low toxicity. Whole-genome sequencing of resistant mutants for target identification can quickly provide a detailed understanding of the mechanism of action of new compounds with antibiotic activity as a path to turning these promising molecules into drugs.

## STAR★Methods

### Key Resources Table

**Table:** 

REAGENT or RESOURCE	SOURCE	IDENTIFIER
**Bacterial and Virus Strains**		

M. tuberculosis	Johns Hopkins University	H37Rv
BL21(DE3)pLysS	Novagen	Cat# 70236-3
M. tuberculosis H37Rv	ATCC	ATCC 27294
Clinical M. tuberculosis isolates, see Table S5	Stellenbosch University: in-house culture bank	N/A
Clinical M. tuberculosis isolates, see Table S5	Strain Collection at Mycobacteriology Laboratory of the Institute of Medical Microbiology, University of Zurich	N/A
M. tuberculosis mc2-7000	Texas A&M University: in-house frozen stock (Sambandamurthy et al., 2006)	N/A
Mycobacterium tuberculosis Erdman	Trudeau Mycobacterial Culture Collection	TMC#107

**Chemicals, Peptides, and Recombinant Proteins**		

Phorbol 12-myristate 13-acetate	Sigma-Aldrich	Cat# P8139
D-Pantothenic acid	Sigma-Aldrich	Cat# P5155
Resazurin	Sigma-Aldrich	Cat# R7017
4-Methylumbelliferyl heptanoate (4-MUH)	Sigma-Aldrich	Cat# M2514
Ammonium sulfate	Sigma-Aldrich	Cat# A4418
Polypropylene glycol P-400	Sigma-Aldrich	Cat# 81350
Fomblin	Sigma-Aldrich	Cat# 317950
Polyethylene glycol 400 (PEG 400)	Hampton Research	Cat# HR2-603
BTC-AM dye	ThermoFisher Scientific	Cat# B6791
Uridine di-phosphoglucuronic acid (UDPGA)	Sigma-Aldrich	Cat# U6751
β-Nicotinamide adenine dinucleotide 2′-phosphate reduced (NADPH)	Sigma-Aldrich	Cat# N1630
β-Nicotinamide adenine dinucleotide (NADP)	Sigma-Aldrich	Cat# N3886
Propranolol	Sigma-Aldrich	Cat# PO884
Glucose-6-phosphate Dehydrogenase (from Baker’s yeast S. cerevisiae)	Sigma-Aldrich	Cat# G7877
Ethoxyresorufin (ER)	Sigma-Aldrich	Cat# E3763
3-Cyano-7-Ethoxycoumarin (CEC)	Sigma-Aldrich	Cat# UC455
Miconazole nitrate	Sigma-Aldrich	Cat# M3512
D-Glucose-6-phosphate sodium salt	Sigma-Aldrich	Cat# G7879
7-methoxy-4-(trifluoromethyl)-coumarin (MFC)	Cypex	Cat# CYP517
7-methoxy-4-(aminomethyl)-coumarin (MAMC)	Cypex	Cat# CYP515
Diethoxyfluorescein (DEF)	Cypex	Cat# CYP531
7-Benzyloxyquinoline (7BQ)	Cypex	Cat# CYP512
Benzoquinone	TCI America	Cat# B0089
ethyl 3-oxo-3-phenylpropanoate	Combi-Blocks	Cat# QA-0159
ethyl 3-(4-methoxyphenyl)-3-oxopropanoate	TCI America	Cat# M1380
Piperidine	Sigma-Aldrich	Cat# 411027
Methylamine solution	Sigma-Aldrich	Cat# 395056
Oxalyl chloride	Sigma-Aldrich	Cat# 221015
Copper(II) trifluoromethanesulfonate	Sigma-Aldrich	Cat# 283673
2,5-Dihydroxybenzaldehyde	Combi-Blocks	Cat# OR-1145
3-methoxy-5-methylphenol	TCI America	Cat# M0895
Palladium(II) acetate	Sigma-Aldrich	Cat# 205869
Boron tribromide	Sigma-Aldrich	Cat# 230367
Trifluoromethanesulfonic anhydride	Sigma-Aldrich	Cat# 176176
Diethyl azodicarboxylate solution	Sigma-Aldrich	Cat# 563110
Phenylmagnesium bromide	Sigma-Aldrich	Cat# 331376
ethyl 5-hydroxy-4-[(4-methyl-1-piperidinyl)methyl]-2-phenyl-1-benzofuran-3-carboxylate (TAM1)	ChemBridge Corporation	ID# 6238794
ethyl 5-hydroxy-4-[(3-methyl-1-piperidinyl)methyl]-2-phenyl-1-benzofuran-3-carboxylate (TAM2)	ChemBridge Corporation	ID# 6241075
ethyl 5-hydroxy-2-phenyl-4-(1-pyrrolidinylmethyl)-1-benzofuran-3-carboxylate hydrochloride (TAM3)	ChemBridge Corporation	ID# 6238866
ethyl 4-(1-azepanylmethyl)-5-hydroxy-2-phenyl-1-benzofuran-3-carboxylate hydrochloride (TAM4)	ChemBridge Corporation	ID# 6240697
ethyl 5-hydroxy-4-(4-morpholinylmethyl)-2-phenyl-1-benzofuran-3-carboxylate (TAM5)	ChemBridge Corporation	ID# 6240924
ethyl 4-[(dimethylamino)methyl]-5-hydroxy-2-phenyl-1-benzofuran-3-carboxylate (TAM6)	ChemBridge Corporation	ID# 5169001
Ethyl 5-hydroxy-2-phenylbenzofuran-3-carboxylate (TAM7)	This paper	N/A
Ethyl 4-benzyl-5-hydroxy-2-phenylbenzofuran-3-carboxylate (TAM8)	This paper	N/A
Ethyl 5-hydroxy-2-phenyl-4-(piperidin-1-ylmethyl)benzofuran-3-carboxylate (TAM9)	This paper	N/A
5-hydroxy-2-phenyl-4-(piperidin-1-ylmethyl)benzofuran-3-carboxylic acid (TAM10)	This paper	N/A
Ethyl 4-(cyclohexylmethyl)-5-hydroxy-2-phenylbenzofuran-3-carboxylate (TAM11)	This paper	N/A
5-hydroxy-N-methyl-2-phenyl-4-(piperidin-1-ylmethyl)benzofuran-3-carboxamide (TAM12)	This paper	N/A
Ethyl 5-hydroxy-2-(4-hydroxyphenyl)-4-(piperidin-1-ylmethyl)benzofuran-3-carboxylate (TAM13)	This paper	N/A
Ethyl 5-methoxy-2-phenyl-4-(piperidin-1-ylmethyl)benzofuran-3-carboxylate (TAM14)	This paper	N/A
Ethyl 2-phenyl-4-(piperidin-1-ylmethyl)benzofuran-3-carboxylate (TAM15)	This paper	N/A
5-hydroxy-2-(4-hydroxyphenyl)-N-methyl-4-(piperidin-1-ylmethyl)benzofuran-3-carboxamide (TAM16)	This paper	N/A
Ethyl 5-hydroxy-2-(6-hydroxypyridin-3-yl)-4-(piperidin-1-ylmethyl)benzofuran-3-carboxylate (TAM17)	This paper	N/A
2-(3-fluoro-4-hydroxyphenyl)-5-hydroxy-N-methyl-4-(piperidin-1-ylmethyl)benzofuran-3-carboxamide (TAM18)	This paper	N/A
6-hydroxy-2-(4-hydroxyphenyl)-N-methyl-4-(piperidin-1-ylmethyl)benzofuran-3-carboxamide (TAM19)	This paper	N/A
Ethyl 6-hydroxy-2-phenyl-4-(piperidin-1-ylmethyl)benzofuran-3-carboxylate (TAM20)	This paper	N/A
Ethyl 5-hydroxy-4-((3-(hydroxymethyl)piperidin-1-yl)methyl)-2-phenylbenzofuran-3-carboxylate (TAM21)	This paper	N/A
Ethyl 5-hydroxy-4-((2-(hydroxymethyl)piperidin-1-yl)methyl)-2-phenylbenzofuran-3-carboxylate (TAM22)	This paper	N/A
Ethyl 5-hydroxy-4-((3-(hydroxymethyl)piperidin-1-yl)methyl)-2-(4-hydroxyphenyl)benzofuran-3-carboxylate (TAM23)	This paper	N/A
5-Hydroxy-4-((3-(hydroxymethyl)piperidin-1-yl)methyl)-N-methyl-2-phenylbenzofuran-3-carboxamide (TAM24)	This paper	N/A

**Critical Commercial Assays**		

PCR Mycoplasma Test kit	Life Technologies	Cat# 409010
QuikChange II Site-Directed Mutagenesis Kit	Agilent Technologies	Cat# 200523
Innovative Grade US Origin Mouse Plasma CD-1	Innovative Research	Cat# IGMS-N
Mouse Microsomes (CD-1)	Life Technologies	Cat# MSMC-PL
Human liver microsomes (HLM) - Female CD1	Xenotech	Lot# 910255
Human CYP1A2 + human CYP reductase	Cypex	Cat# CYP/EZ001
Human CYP2C9 + human CYP reductase + purified human cytochrome b5	Cypex	Cat# CYP/EZ037
Human CYP2C19 + human CYP reductase	Cypex	Cat# CYP/EZ008
Human CYP2D6 + human CYP reductase	Cypex	Cat# CYP/EZ007
Human CYP3A4 + human CYP reductase + purified human cytochrome b5	Cypex	Cat# CYP/EZ005
Membrane protein (control)	Cypex	Cat# CYP/EZ003
Safety Profiling	AbbVie, IL, USA	N/A

**Deposited Data**		

Pks13-TE domain (apo) structure	This paper	PDB: 5V3W
Pks13-TE:TAM1 complex structure	This paper	PDB: 5V3X
Pks13-TE:TAM16 complex structure	This paper	PDB: 5V3Y
Pks13-TE:D1607N mutant structure	This paper	PDB: 5V3Z
Pks13-TE:TAM6 complex structure	This paper	PDB: 5V40
Pks13-TE:TAM5 complex structure	This paper	PDB: 5V41
Pks13-TE:TAM3 complex structure	This paper	PDB: 5V42

**Experimental Models: Cell Lines**		

Human Dermal Fibroblast	ATCC	ATCC PCS-201-010
THP-1 cells	ATCC	ATCC TIB-202
hERG T-REx-CHO Cell line	ThermoFisher Scientific	Cat# K1237

**Experimental Models: Organisms/Strains**		

Mouse: ND4 Swiss Webster outbred	Envigo, Indianapolis, IN	Strain code: 032
Mouse: BALB/c AnNCrl	Charles River laboratories, Wilmington, MA	Strain code: 028

**Oligonucleotides**		

LIC Primer: wt Pks13-TE Forward: TACTTCCAATCCAATGCCCAGATCGATGGGTTCGTCCGCAC	This paper	N/A
LIC Primer: wt Pks13-TE Reverse: TTATCCACTTCCAATGTTATCACTGCTTGCCTACCTCACTTGTTCG	This paper	N/A
Primer: Site directed mutagenesis Pks13-TE:D1607N Forward: GAGGAGCTCGACAACGAGGGCCAGG	This paper	N/A
Primer: Site directed mutagenesis Pks13-TE:D1607N Reverse: CCTGGCCCTCGTTGTCGAGCTCCTC	This paper	N/A
Primer: Site directed mutagenesis Pks13-TE:D1644G Forward: CCGGGCGATCGGCACCGCCCAGA	This paper	N/A
Primer: Site directed mutagenesis Pks13-TE:D1644G Reverse: TCTGGGCGGTGCCGATCGCCCGG	This paper	N/A

**Recombinant DNA**		

Plasmid: pMCSG-19b	Midwest Center for Structural Genomics (http://bioinformatics.anl.gov/mcsg/technologies/vectors.html)	N/A
Pks13-TE-pMCSG-19b	This paper	N/A
Pks13-TE:D1607N_pMCSG-19b	This paper	N/A
Pks13-TE:D1644G_pMCSG-19b	This paper	N/A

**Software and Algorithms**		

Masslynx 4.1	Waters	N/A
MetaboLynx	Waters	N/A
HKL2000	Otwinowski and Minor, 1997	N/A
CCP4 suite	http://www.ccp4.ac.uk/	N/A
Phenix Suite	Adams et al., 2010	N/A
UCSF Chimera	Pettersen et al., 2004	N/A
Coot	Emsley and Cowtan, 2004	N/A
PyMOL 1.4	Schrodinger, LLC	N/A
Prism 4.0, 5.0	GraphPad Software	N/A
IN Cell Investigator image analysis software for IN CELL ANALYZER 2000	GE Life Sciences	N/A
CompuSyn software	http://www.combosyn.com/	N/A
SigmaPlot 11.0	Jandel Corporation	N/A
CAVER 3.0 PyMOL plugin	http://www.caver.cz	N/A
Molsoft ICM-Pro 3.8	http://www.molsoft.com	N/A

**Other**		

Rapid Equilibrium Dialysis (RED) Device Single Use Plate with Inserts, 8K MWCO	ThermoFisher Scientific	Cat# 90006
HiLoad 16/600 Superdex 200 pg Column	GE Life Sciences	Cat# 28989335
Rifampin	Sigma-Aldrich	Cat# R3501-5G
Rifampicin	Sigma-Aldrich	Cat# R3502
Ethambutol	Sigma-Aldrich	Cat# E4630
Streptomycin	Sigma-Aldrich	Cat# S6501
Isoniazid	Sigma-Aldrich	Cat# I3377
Verapamil	Sigma-Aldrich	Cat# V4629
Penicillin-Streptomycin (10,000 U/mL	Life technologies	Cat# 15140122
DMEM, high glucose, GlutaMAX Supplement	Life technologies	Cat# 10566-016
Fetal Bovine Serum	Corning	Cat# 35-015-CV
Gentamycin	GIBCO	Cat# 15710064
Carbenicillin	Caisson Labs	Cat# C033-100GM
Chloramphenicol	GoldBio	Cat# C-105-100
Warfarin PESTANAL, analytical standard	Sigma-Aldrich	Cat# 45706
Tyloxapol	Sigma-Aldrich	Cat# T8671
BBL Middlebrook 7H11 Selective Agar	Becton Dickinson	Cat# 8801671
Difco Middlebrook 7H10 agar	Becton Dickinson	Cat# 262710
Difco Middlebrook 7H9 broth	Becton Dickinson	Cat# 271310
Middlebrook OADC enrichment	Becton Dickinson	Cat# 212351/212240
BACTEC MGIT 960 tubes (7 ml)	Becton Dickinson	Cat# 245122
Bedaquiline (TMC207)	Advanced ChemBlocks Inc	Cat# 10288, Lot# 10355

### Contact for Reagent and Resource Sharing

Further information and requests for resources and reagents should be directed to and will be fulfilled by the Lead Contact, James C. Sacchettini (sacchett@tamu.edu). Texas A&M University requires that a material transfer agreement (MTA) be signed for transfer of materials. Small amounts of compounds synthesized for this study will be made available as reference standards when a sufficient supply is available.

### Experimental Model and Subject Details

#### Animal studies

All animal experiments described in this manuscript followed protocols approved by the respective Institutional Animal Care and Use Committee’s at Colorado State University, Johns Hopkins University and Texas A&M University.

#### Pharmacokinetic studies in mice

Swiss-Webster female mice (∼20 g each) (Envigo, Indianapolis, IN) were used in the PK study. The formulation of TAM16 was prepared in 90% canola oil and 10% DMSO. Mice were dosed at 100 mg/kg by oral gavage at 0 hr and 4 hr time points in 0.1 mL aliquots to yield a final dose per day of 200 mg/kg. Three mice were used per time point. The mice were anesthetized with ketamine-xyalzine and ∼0.1 mL of blood was drawn for survival bleeds at 0.5, 1.5, 5 hr, time points, and ∼0.3 mL of blood was drawn for terminal bleeds at 4, 8 and 12 hr time points. Blood was collected from the brachial region. The blood samples were centrifuged (5,000 x *g*, 15 min) for plasma separation. 500 μL methanol containing 0.1% formic acid was then added to a 50 μL serum aliquot to precipitate the protein. The methanol precipitation step was performed twice to ensure maximum extraction of TAM16 from the serum. After removing the precipitate by centrifugation, the clear supernatant was evaporated to dryness. The dry samples were then reconstituted with 50 μL methanol and subjected to LC-MS analysis on a micrOTOF-Q II mass spectrometer (Bruker Daltonics Inc.) hyphenated with an Agilent 1200 Infinity series HPLC with temperature controlled autosampler and photodiode array detector. 2.1 X 100 mm Atlantis T3, 5 μm C18 HPLC column (Waters) was used in the analysis at a flow rate of 0.5 ml.min^-1^. The mobile phase consisted of water with 0.1% formic acid as Solvent line A and acetonitrile with 0.1% formic acid as solvent line B. The gradient conditions were maintained as follows: 90% A, 10% B to 100% B in 8 min; 100% B held for 4 min; 100% B back to 90% A, 10% B in 2 min; 90% A, 10% B held for 3 min. The injection volume of the analyte was 10 μL and MS was operated in the positive mode with electrospray ionization at source. Mass spectra were monitored in the range of (m/z) 50 to (m/z) 1000.

#### Efficacy studies in mice

##### BALB/c acute TB infection mouse model

For this study, mice (Charles River Labs, Wilmington, MA) were maintained under specific pathogen-free conditions and fed water and chow ad libitum. For drug preparation, isoniazid (INH, Sigma) was dissolved in sterile distilled water. A dosing solution of 1 mg/ml was prepared weekly and kept at 4°C. TAM16 was suspended in vehicle of canola oil and 10% DMSO and stored at 4°C for up to 1 week. A total of 70 female BALB/c mice (7-8 weeks-old) were aerosol-infected with *M. tuberculosis* H37Rv using the Inhalation Exposure System (Glas-Col Inc., Terre Haute, IN) calibrated to deliver ∼10^4^ (high-inoculum) colony-forming units (CFU) per mouse lung. After aerosol infection, mice were blindly randomized into treatment groups: no drug (negative control) and drug treated (positive control), and were treated daily via oral gavage administered in 0.2 mL 5 days per week for 2 weeks at 200 mg/kg once daily. Five mice from each group were sacrificed on the day after infection, on the day of treatment initiation (Day 0), and on day 14 after treatment to determine the numbers of CFU implanted in the lungs, pretreatment baseline CFU counts and the CFU counts after 14 days of treatment, respectively. Animal body weights were recorded at the time of sacrifice. The lungs of sacrificed mice were homogenized in 2.5 mL PBS. Lung homogenates were plated on selective 7H11 plates (containing cycloheximide (50 μg/ml), carbenicillin (100 μg/ml), polymyxin B (200 U/ml), and trimethoprim (20 μg/ml)) for CFU enumeration. Statistical analysis was done on CFU data derived from 3 to 5 mice per group. Log-transformed CFU were used to calculate means and standard deviations. Comparisons of data among experimental groups were performed by Student’s t test. Group means were compared by one-way analysis of variance (ANOVA) with Dunnett’s post-test (Day 0 or untreated controls versus treatment groups) or Bonferroni comparison (all treatment groups), using GraphPad Prism version 4 (GraphPad, San Diego, CA). Values of p < 0.05 were considered to be statistically significant.

##### BALB/c chronic TB infection mouse model

In this study, 6- to 8-week-old female specific-pathogen-free immunocompetent BALB/c mice (Charles River, Wilmington, MA) were infected via a low-dose aerosol exposure to *M. tuberculosis* Erdman in a Middlebrook aerosol generation device (Glas-Col Inc., Terre Haute, IN). One day post aerosol infection, three mice were sacrificed to verify the uptake of an average of about 100 CFU of bacteria per mouse. Following infection, the mice were randomly divided into treatment groups. Negative control mice remained untreated. Positive control mice received INH (at 25 mg/kg of body weight). Each group consisted of five mice at each time point. Treatment was started day 27 post aerosol and continued for 4 weeks. Five infected mice were killed at the start of treatment as pretreatment controls. Drugs were administered in canola oil, 5 days per week by oral gavage. To determine drug efficacies, mice from each treatment group were sacrificed after 4 weeks of treatment. The mice were humanely euthanized by CO_2_ inhalation. The spleens and left lung lobes were aseptically removed and disrupted in a tissue homogenizer. The number of viable organisms was determined by serial dilution of the homogenates on nutrient Middlebrook 7H11 agar plates (GIBCO BRL, Gaithersburg, MD). The plates were incubated at 37°C in ambient air for 4 weeks prior to determine the total number of culturable mycobacteria per organ. For statistical analysis, the number of culturable mycobacteria were converted to logarithms, which were then evaluated by a one-way analysis of variance, followed by a multiple-comparison analysis of variance by a one-way Tukey test (SigmaPlot software program). Differences were considered significant at the 95% level of confidence.

##### Combination studies in mouse model of chronic TB

Six- to 8-week-old female specific-pathogen-free immunocompetent BALB/c (Charles River, Wilmington, MA) were infected via a low-dose aerosol exposure to *M. tuberculosis* Erdman (∼50-100 bacilli/mouse) using the Glas-Col Inhalation Exposure System. One day post aerosol infection, five mice were sacrificed to determine bacterial uptake. Following infection, the mice were randomly divided into treatment groups of six mice each. Negative control mice remained untreated. At Day 28 post-infection 6 mice were sacrificed to determine bacterial load in the lungs at the start of therapy. Therapy administered via oral gavage, was started day 28 post-aerosol infection and continued for 4 and 8 weeks. Drugs were administered by gavage daily, for 5 days a week (Mon-Fri), in a volume of 200 μl/animal/drug. For animals receiving two drugs, RIF was dosed first and then at least 1 hr later, the second drug was administered. To determine drug efficacies, six mice from each group (untreated, vehicle, TAM16, RIF, INH and combination treatment mice) were sacrificed after 4 and 8 weeks of treatment following a three day drug washout period after the last day of dosing. For the companion plasma PK studies (plasma collected at both C_max_/C_min_), test bleeds were performed via the submandibular route using a GoldenRod lancet during last week of treatment (week 8) from n = 3 mice from TAM16 only and TAM16+RIF treatment groups. For statistical analysis, the viable CFU counts were converted to logarithms, which were then evaluated by a one-way ANOVA followed by a pairwise multiple comparison using the Dunnett’s test (SigmaPlot software program). Differences were considered significant at the 95% level of confidence.

#### Cell lines and bacterial strains

Human Dermal Fibroblast (HDF) cells were purchased from ATCC (catalog number PCS-201-010) and were cultured in DMEM (Lonza) media supplemented with 10% fetal bovine serum (Lonza) and penicillin/streptomycin (Lonza). THP-1 cells were purchased from ATCC (catalog number TIB-202) and were differentiated with PMA (100 nM) for 3 days prior to infection studies. For assaying hERG activity, an inducible hERG T-REx™-CHO Cell line was purchased by AbbVie from ThermoFisher (catalog number K1237). Cell lines obtained from ATCC were tested for mycoplasma contamination by PCR Mycoplasma test kit (MD Bioproducts). Purchased cell lines were not further authenticated.

BL21(DE3)pLysS competent *E. coli* cells were from Novagen (catalog number 70236-3). *M. tuberculosis mc*^*2*^*7000* (Sambandamurthy et al., 2006) was obtained from in-house frozen stock (−80°C) at Texas A&M University. To obtain starter culture, a 1 mL aliquot of frozen cells was thawed and cultured in 7H9 media supplemented with OADC (Middlebrook), 0.05% Tyloxapol (Sigma), and 25 μg/ml pantothenate for 6-7 days to an OD_600_ of 1–1.5. Clinical *M. tuberculosis* isolates were selected from the culture collections at the Department of Molecular Biology and Human Genetics, Stellenbosch University, South Africa, and at the Mycobacteriology Laboratory of the Institute of Medical Microbiology, University of Zurich, Switzerland (for a list of strains and mutations see Table S5). The identity of the isolates was determined by IS*6110* restriction fragment length polymorphism (van Embden et al., 1993) and their phylogenetic lineages were assigned by spoligotyping (Kamerbeek et al., 1997, Streicher et al., 2007). Whole genome sequencing showed that all of the drug-susceptible isolates lacked high confidence resistant markers which suggests susceptibility against the conventional first- and second- line drugs.

### Method Details

#### Cloning and overexpression of *Mtb* Pks13 TE domain constructs

The TE domain construct corresponding to the predicted TE domain in *Mtb pks13* gene (Rv3800c) was made by PCR from the *Mtb* H37Rv genomic DNA as the template (forward primer: 5′ – TAC TTC CAA TCC AAT GCC CAG ATC GAT GGG TTC GTC CGC AC – 3′, reverse primer: 5′ – TTA TCC ACT TCC AAT GTT ATC ACT GCT TGC CTA CCT CAC TTG TTC G – 3′). The amplified DNA fragments were incorporated into the pMCSG-19b vector by ligation independent cloning (LIC) to yield TEV protease cleavable N-terminal His_6_-tagged TE domain construct (Donnelly et al., 2006). The Pks13-TE-pMCSG-19b vector was transformed into *E. coli* BL21(DE3)pLysS cells (Novagen) and the transformed cells were grown at 37°C in LB media containing carbenicillin (100 μg/ml) and chloramphenicol (34 μg/ml) to an OD_600_ of 0.6. Expression of TE construct was induced with 0.5 mM IPTG, and cells were harvested after 16 hr of growth at 20°C. The D1607N and D1644G mutants of Pks13 TE domain were constructed using the QuikChange II site-directed mutagenesis kit (Agilent Technologies). The mutations were confirmed by DNA sequencing. Mutant plasmids were transformed into *E. coli* BL21(DE3)pLysS cells, and mutant proteins were expressed by induction with 0.5 mM IPTG at 20°C for 18 hr.

#### Purification of Pks13 TE domain

The harvested cells were resuspended in the lysis buffer (50 mM Tris-HCl pH 8.0, 0.5 M NaCl, 10% (v/v) glycerol, 1 mM β-mercaptoethanol (BME) and DNase) and lysed by French press. The resulting cell extract was clarified by centrifugation (15,000 x *g*) for 1 hr at 4°C. The cleared supernatant was loaded onto a Ni-affinity column and the His-tagged TE domain constructs were eluted with a linear gradient of 10-250 mM imidazole in 20 mM Tris-HCl, pH 8.0 and 0.5 M NaCl. The peak fractions were pooled and the His-tag was cleaved by overnight incubation with TEV protease in dialysis buffer (20 mM Tris-HCl pH 8.0, 10% (v/v) glycerol and 1 mM DTT). The TEV cleaved protein was passed through Ni-column to remove any uncleaved His-tagged protein using 20 mM Tris-HCl (pH 8.0) with 100 mM NaCl and 1 mM BME. His-tag cleaved protein eluted in the flow-through and was concentrated for loading onto a Superdex-200 gel filtration column (GE Healthcare). The 283 residue long TE domain starting from residue 1451 in full length Pks13 (referred to as Pks13-TE in this paper) eluted under a single peak as a monomer (∼32 kDa) from the gel filtration column and was > 95% pure as observed by SDS-PAGE. The purified protein was concentrated to 20-25 mg/ml, flash-frozen and stored at −80°C. The TE domain mutants were purified using the same protocol as for the wild-type Pks13-TE domain constructs. Both the mutants and the wt Pks13-TE domain protein constructs have the amino acids SNA from the TEV cleavage site appended to the N terminus.

#### Crystallization and soaking with ligands

Initial screening for crystallization conditions for the Pks13-TE domain was done using sitting drop method using 1 μL of purified protein (15-20 mg/ml) and 1 μL of crystallization buffer from the well solution. The Pks13-TE crystals were obtained in crystallization buffer containing 0.1 M Tris-HCl, pH 8.5 and 2.0-1.8 M ammonium sulfate as precipitant. Crystals were further optimized by using polypropylene glycol P-400 as an additive at 2%–5% (v/v) in the original condition. To obtain Pks13-TE-inhibitor complex crystals, soaking of the inhibitors was done by transferring apo-Pks13-TE crystals into a drop consisting of 0.1 M Tris-HCl, pH 8.5 and 2-2.2 M ammonium sulfate with 1-2.5 mM inhibitor added from a DMSO stock keeping the final DMSO concentration at < 5%, and incubated at 18°C for 4-48 hr. Crystals of the Pks13-TE:D1607N mutant were obtained by sitting drop method at 18°C. The crystallization drops contained an equal volume of the protein solution (15-20 mg/ml) and mother liquor (0.1 M HEPES, pH 7.5, 2%–4% (v/v) PEG 400, and 1.8-2 M ammonium sulfate), and the diffraction quality crystals were obtained within 2 weeks.

#### Data collection and processing

For diffraction data collection the crystals were cryo-protected using Fomblin (Sigma) and flash frozen in liquid nitrogen. High resolution data was collected at wavelengths of 0.98 – 1.03 Å on the beamlines 19-ID and 23-ID at the Advanced Photon Source (APS) of the Argonne National Laboratory, Chicago, IL, USA. All the datasets were processed and scaled with *HKL2000* (Otwinowski and Minor, 1997). Analysis of the integrated and scaled data by Xprep (Sheldrick, 2008) indicated that Pks13-TE crystallized in P2_1_2_1_2 space group. Solvent content analysis in CCP4 suite indicated the presence of two molecules (V_M_ 2.16, V_S_ 43.2%) in the asymmetric unit (Matthews, 1968).

#### Determination of Pks13-TE structures and model refinement

The structure of the Pks13-TE domain was solved by molecular replacement method (MR) using *E. coli* EntF (PDB: 3tej) structure, as search model. A single MR solution was obtained using Phenix AutoMR (Adams et al., 2010) which was input into the AutoBuild wizard to generate the initial model for apo-Pks13-TE. The initial model was improved by further manual rebuilding in COOT (Emsley and Cowtan, 2004). The final model was obtained after iterative cycles of model building and Phenix refinement with simulated annealing yielding a 1.72 Å resolution apo-Pks13-TE model with R_cryst_ of 16.9% and an R_free_ of 20.1% with good stereochemistry (Table S2). The final refined apo-model has two chains, designated A and B, a fragment of additive PPG P400 and 471 water molecules in the asymmetric unit. The crystal structures Pks13-TE-inhibitor complex structures, as well as the D1607N mutant structures were refined with simulated annealing (start temperature 5000 K, Phenix). Inspection of electron density maps showed clear |F_o_-F_c_| positive difference density for the ligands which were fit into the density using Ligandfit routine in Phenix (Terwilliger et al., 2006). The ligand model and geometry restraint files were created in ELBOW BUILDER of the Phenix suite (Moriarty et al., 2009). Iterative cycles of model building and NCS-restrained maximum likelihood refinement with simulated annealing yielded high quality models for Pks13-TE-inhibitor complexes (Tables S2 and S3). In all of the structures > 98% of residues are placed in the favored region of the Ramachandran plot (MolProbity, Chen et al., 2010). Figures of the structures were made with UCSF Chimera package (Pettersen et al., 2004) and PyMOL Molecular Graphics System version 1.4.1 (Schrodinger, LLC). Structural analysis of Apo Pks13-TE for the identification of tunnels and channels was done using CAVER 3.0 PyMol plugin (Chovancova et al., 2012). Electrostatic surface potentials were calculated using APBS (Baker et al., 2001) and displayed using the APBS plugin for PyMOL. Atomic coordinates and structure factors for the reported crystal structures (Tables S2 and S3) have been deposited with the Protein Data Bank under accession codes: PDB: 5V3W (Apo Pks13-TE), PDB: 5V3X (Pks13-TE:TAM1), PDB: 5V3Y (Pks13-TE:TAM16), PDB: 5V3Z (Pks13-TE(D1607N)), PDB: 5V40 (Pks13-TE:TAM6), PDB: 5V41 (Pks13-TE:TAM5), and PDB: 5V42 (Pks13-TE:TAM3).

#### Enzyme assay

Activity of Pks13-TE was assessed using 4-methylumbelliferyl heptanoate (4-MUH, Sigma) as a fluorogenic substrate in a 96-well plate format (Richardson and Smith, 2007). To make initial velocity measurements, Pks13-TE (1 μM) in 0.1 M Tris-HCl, pH 7 buffer was incubated with different concentrations of 4-MUH (2-150 μM in DMSO) in a 100 μL reaction volume, and the fluorescence of the hydrolyzed product 4-methylumbelliferone was read (excitation at 355 nm and emission at 460 nm) (PolarStar Omega plate reader BMG Labtech) at 5-10 min intervals over 80-120 min. The reaction rate was observed to be linear in the measured range. 4-MUH in buffer alone was included as a control to quantify its background hydrolysis. Data points were plotted as an average of triplicates and each experiment was repeated twice independently. The initial velocity data was curve fit to Michaelis-Menten equation by nonlinear regression using Prism software (GraphPad) to determine the kinetic parameters *K*_*m*_ and *V*_*max*_. The assay and data analysis for Pks13-TE mutants was done the same way as that for the wild-type protein with the 4-MUH concentration varying from 2 to 300 μM.

#### IC_50_ determination

To determine the potency of TAM1 and its analogs against wt Pks13-TE, the compounds were tested at concentrations ranging from 0.012 to 20 μM in a 96-well plate format. The reaction mix contained 0.1 μM Pks13-TE in 0.1 M Tris-HCl, pH 7 buffer with 1 μL of each dilution of the compound or DMSO in a total volume of 99 μL. The reaction was initiated by addition of 1 μL of 2 mM 4-MUH in DMSO (20 μM final concentration) to the reaction mix. Initial velocity data was obtained by monitoring increase in the fluorescence due to hydrolysis of the substrate using PolarStar Omega plate reader at 10 min intervals over 110 min. The data points were collected in triplicate and the averaged value was used to generate concentration-response plots for TAM1 and its analogs. The IC_50_ value for each compound was obtained by nonlinear regression curve fitting of a four-parameter variable slope equation to the dose-response data using Prism software. The IC_50_ values of TAM1 for Pks13-TE mutants were determined in the same way as that for wt Pks13-TE, using inhibitor concentration range of 0.04 to 40 μM.

#### Whole cell activity and cytotoxicity testing

Whole cell testing for determining MIC was done using Alamar blue assay in 96-well plates (Franzblau et al., 1998). *Mtb mc*^2^-7000 cells were grown to an OD_600_ of 1–1.5. The cells were then diluted into testing media (7H9 media with 0.2% dextrose, 0.085% NaCl, 0.05% Tyloxapol, and 25 μg/ml pantothenate) to an OD_600_ of 0.01 and dispensed into testing plates at 196 μL per well. Then the compounds were added (4 μL) as a 2-fold serial dilution in DMSO (2% DMSO final in each well). The test plates also had a DMSO only control and a Rifampicin control. The plates were incubated with shaking at 37°C for 6 days and then stained with resazurin (Sigma) for an additional 2 days at 37°C. After staining the fluorescence of reduced resazurin was read (λ_*Ex*_ = 544 nm, λ_*Em*_ = 590 nm) using PolarStar Omega plate reader. The fluorescence data were plotted as percent growth inhibition against the compound concentration and curve fitting was done by nonlinear regression using Prism software. Minimum inhibitory concentration (MIC) values, defined as the concentration giving 50% growth inhibition, were determined from the fitted curves.

Compounds were tested for toxicity by the Human Dermal Fibroblast (HDF) cytotoxicity assay. The cells were cultured in DMEM (Lonza) media supplemented with 10% fetal bovine serum (Lonza) and penicillin/streptomycin (Lonza). For setting the cytotoxicity assay, compound stocks were serially diluted starting from the highest concentration of 100 μL in phosphate buffered saline (PBS) plus 10% DMSO. On the day of assay, HDF cells were trypsinized, counted and resuspended at a concentration of 64,000 cells/ml in the media. Cells were plated, overlaid with the compound serial dilutions and incubated at 37°C. After 48 hr, resazurin dye was added and the assay plates were cultured for another 24 hr. The next day the absorbance of the resazurin was measured on a microplate reader (BMG Labtech) to assess cell death.

#### THP-1 Drug Efficacy Assay

THP-1 monocytes were differentiated with PMA (100nM) for 3 days prior to infection and seeded at 40k cells/well in a 96-well dish coated with 0.1% gelatin. *Mtb mc*^*2*^*7000* cells constitutively expressing fluorescent reporter mCherry were incubated with the THP-1 for 2 hr, washed gently, and incubated with gentamycin (10 μg/mL) for 2 hr to kill extracellular *Mtb mc*^*2*^*7000*. Cells were then washed and incubated with drug (1% DMSO final). Fluorescence was measured on days 0 and 5 post infection using a GE IN Cell Analyzer 2000 at 20x magnification and analyzed using the complementary GE software suite. Fold change in fluorescence was normalized to rifabutin (10 μM) as the positive control. All experiments were performed with technical triplicates and were repeated six times independently.

#### Isolation of resistant mutants and whole-genome sequencing

For the isolation of TAM16 resistant mutants, ∼10^9^ *Mtb mc*^*2*^*7000* cells were plated onto replicate 7H10 plates containing either 0.9 μM (10x MIC) or 1.8 μM (20x MIC) of TAM16. In addition, serial dilutions of *Mtb mc*^*2*^*7000* culture were plated out on 7H10 plates containing no TAM16. Plates were screened for the appearance of resistant mutants after 3-4 weeks of incubation at 37°C, and the frequencies of resistance were determined by dividing the CFUs on TAM16 containing plates by the CFUs on TAM16-free plates. Genomic DNA from the mutants was extracted by CTAB-lysozyme method as described previously (Larsen et al., 2007) and subjected to whole-genome sequencing for identification of single nucleotide polymorphisms (Ioerger et al., 2013).

#### In vitro activity of compounds against clinical *M. tuberculosis* isolates

The Mycobacterial Growth Indicator Tube drug susceptibility testing system (MGIT 960 DST) was used to determine the MICs of TAM16 against drug-susceptible and drug-resistant clinical *M. tuberculosis* isolates with diverse genetic background.

##### MGIT DST at University of Zurich

Drug susceptibility was tested using the proportion method and MGIT equipped with EpiCenter TB eXiST as described previously (Springer et al., 2009). In brief, bacterial suspensions were prepared from MGIT subcultures. TAM16 was dissolved in DMSO and two-fold dilution series in DMSO were prepared. 0.08 mL of each drug dilution was inoculated into the MGIT vial (final DMSO concentration 1%) together with 0.5 mL of the bacterial suspension. For preparation of the drug-free growth control tube, the bacterial suspension was diluted 1:100 with sterile saline solution, and then 0.5 mL was inoculated into the MGIT vial together with 0.08 mL DMSO. Results were interpreted as follows: at the time the growth unit (GU) value for the drug-free control was > 400, the strain was categorized as susceptible when the GU of the drug-containing tube was < 100 and as resistant, if the GU was ≥ 100. The MIC of each strain was defined at the lowest drug concentration that was categorized as sensitive per the definition above. The epidemiological cut-off (ECOFF) value is the MIC value identifying the upper limit for the wild-type type-population.

##### MGIT DST at Stellenbosch University

Bacterial suspensions were prepared from frozen stock (−80°C) in MGIT 960 medium. These cultures were then sub-cultured and grown at 37°C for 2 additional days after the MGIT tube became positive before they were used as inocula (∼1 × 10^5^ CFU/ml). The compounds were dissolved in 100% DMSO to obtain stock solutions of 1680 mM. Stock solutions were further diluted (1:20) to a concentration of 84 mM in 50% DMSO followed by serial 2-fold dilutions ranging from 84 mM to 1.3 mM. From each dilution, 0.1 mL was transferred into MGIT tubes containing 7.0 mL modified Middlebrook 7H9 broth base supplemented with 0.8 mL OADC. The tubes were then inoculated with 0.5 mL of the test organisms for final 2-fold drug concentrations ranging from 1.0 to 0.015 mM. A drug-free 1:100 diluted inoculum was included per drug set as control to indicate 1% growth according to the proportion method. Results were based on a threshold growth unit (GU) reading of 400 by the drug-free control at 37°C in MGIT 960. Drug-containing tubes with GU values of ≥ 100 at the time when the drug-free control reach a value of 400 were considered resistant and those with values < 100 were recorded as susceptible. The MIC was defined as the lowest drug concentration that inhibits growth of more than 99% of the bacterial inoculum.

#### In vitro synergy evaluation

TAM16 was evaluated in two-drug combination studies for interactions with INH, RIF and EMB in vitro against *Mtb mc*^*2*^*7000* by combination index (CI) method (Chou, 2006). Briefly, TAM16 was combined at a constant ratio of 1:3; 1:0.5 and 1:30 with INH, RIF and EMB, respectively, based on the ratio of the MICs of the individual drugs to TAM16 to obtain equipotency combination ratios, and a 2-fold dilution series from the mixtures was prepared in DMSO. The dilution series of each drug combination as well as the serial dilutions of individual drugs in the combination were then tested in the same 96-well assay plate to obtain dose response data. Fractional inhibition was calculated by dividing the fluorescence data from treatments by the fluorescence from the DMSO only control wells. The CI and dose-reduction index (DRI) were calculated from the dose response data using CompuSyn software (http://www.combosyn.com) for determining the mode of interaction with CI < 1, CI = 1 and CI > 1 indicating synergistic, additive, and antagonistic interactions, respectively.

#### Kinetic solubility determination

The procedure for determining kinetic solubility of Pks13-TE inhibitors was derived and modified from methods described previously (Alelyunas et al., 2009). A 10 mM stock solution of TAM16 was prepared in 100% DMSO. 5 μL of the stock solution was added to 495 μL of sodium phosphate buffer (pH 7.4) taken in the wells of a 2 mL volume deep 96-well polypropylene plate (USA Scientific) to get a final concentration of TAM16 of 100 μM and containing 1% final DMSO concentration. A small stir bar was placed in the well and the plate was left on a stir plate with constant stirring for 24 hr at 25°C. At the end of the 24 hr the plate was centrifuged at 1,000 x *g* to precipitate any un-dissolved material. The supernatant solution was diluted 10-fold in sodium phosphate buffer (pH 7.4) and an aliquot was analyzed on the LC-MS as described under PK studies. TAM16 dissolved in methanol at 10 μM concentration was injected into the LC-MS and used as a single point calibration to estimate the concentration of TAM16 in the aqueous solution.

#### Plasma protein binding

Protein binding of TAM16 in mice plasma was determined using a Rapid Equilibrium Dialysis (RED) kit (ThermoFisher Scientific) with LC-MS analysis. TAM16 (10 μg/mL) and mice plasma with 1% DMSO were added to one side of the single-use RED plate dialysis chamber having an 8 kD MW cutoff membrane. PBS was added to the other side of the membrane. The plate was sealed and left on a shaker at 250 rpm in a 37°C incubator. After 5 hr of dialysis a 50 μL aliquot was collected from both sides of the membrane. 50 μL PBS was added to the plasma sample and 50 μL of plasma was added to the PBS sample in order to minimize matrix effect. Then 200 μL acetonitrile containing 1 μg/ml warfarin (internal standard) was added to precipitate the protein. The sample was mixed well and centrifuged. The clear supernatant was separated from the precipitate and 200 μL water containing 0.1% formic acid was added to the supernatant. 10 μL of the sample was used for LC-MS analysis as described above. The experiment was performed in triplicate.

##### Calculation of plasma protein binding

$$\text{\%}\;bound=\left[\frac{Pl-Bu}{Pl}\right]\times 100$$

%unbound=100−%bound

Where,

*Pl* = Ratio of mass intensities of TAM16 and Warfarin (Internal Standard) determined on the plasma side of the membrane.

*Bu* = Ratio of mass intensities of TAM16 and Warfarin (Internal Standard) determined on the buffer (PBS) side of the membrane.

#### Microsomal stability assay

20 mg/mL stock solution of mouse microsome S9 fractions (Life Technologies) were diluted to a 0.55 mg/mL working stock in 50 mM potassium phosphate buffer (pH 7.4). 0.5 μM final concentration of the compound was used. Verapamil was used as a control drug molecule to validate the assay. The reaction was started by adding 1 mM (final concentration) of freshly prepared NADPH solution. Two negative controls were used; microsomes inactivated by heating at 55°C for 10 min in a water bath, and phosphate buffer in the place of NADPH. Aliquots were removed from this reaction mix at time intervals of 0, 3, 6, 9, 15, 30, 45 and 60 min. The aliquots were added to vials containing 37% acetonitrile, water and an internal standard (0.1 μg/mL warfarin). The mixture was centrifuged to remove precipitated protein and the clear supernatant solution was evaporated to dryness. The dry sample vials were reconstituted with 50 μL methanol and used for LC-MS analysis as described in PK studies section.

##### Calculation of Intrinsic Clearance

Ratio of the mass intensities of TAM16 and the internal standard (Warfarin) were then plotted to fit an exponential decay curve and the rate constant (k) was derived from the curve. Intrinsic clearance was then calculated using the following equation:

$$CLi\left(ml.min^{-1}.g^{-1}\;liver\right)=k\times V\times Microsomal\;protein\;yield$$

Where,

*CLi* = Intrinsic Clearance.

*k* = rate constant.

*V* = incubation volume.mg^-1^ protein added = 1 × 0.5^−1^ = 2 ml.mg^-1^ protein.

Microsomal protein yield is a standard used for all *species* = 52.5 mg protein.g^-1^ liver.

#### Physicochemical properties calculations

Physicochemical properties (clogP, logD, TPSA and pKa) were calculated using ChemAxon chemistry engine as implemented on the Collaborative Drug Discovery web portal (www.collaborativedrug.com) and are summarized in Table 3.

#### Glutathione (GSH) and methoxylamine (MA) trapping

Metabolic stability of TAM16 was assessed following incubations with human liver microsomes (HLMs) (20 mg/mL, supplied by Xenotech as a pooled batch of 50 donors). The incubations were performed in a medium containing 0.05 M phosphate buffer (pH 7.4) with TAM16 at a final concentration of 10 μM in the presence of 0.5 mg/mL microsomal protein, 5 mM GSH/MA and a NADPH regenerating system (including 2.3 mM Glucose-6-Phosphate, 1.8 mM NADP, and 0.5 U/mL glucose 6-phosphate dehydrogenase in 2% (w/v) sodium bicarbonate). Positive controls included Clozapine and 17α-ethynylestradiol (EE) for GSH trapping and Amodiaquine for MA trapping at a final concentration of 5 μM. A ‘no compound control’ replacing TAM16/positive controls with DMSO was also included. Samples prepared in 96-plates were incubated on a shaking water bath/incubator at 37°C for 60-90 min and after incubation, reactions were stopped by adding acetonitrile (analytical grade). The incubation mixtures were then centrifuged at 2800 rpm for 10 min at 4°C. A 150 μL aliquot of supernatant was added to 150 μL water before analysis by UPLC-MS/MS (Waters Acquity UPLC with DAD and Waters Xevo Q-TOF). Chromatography was performed on a Waters BEH C18 (50 × 2.1mm 1.7μm) column at a flow of 0.5 mL/min with an injection volume of 5 μL. Detection of metabolites was performed by analysis with Metabolynx XS software (Waters) and by manual data searching. Analysis of positive controls was used to ensure that the assay was functional. Metabolic stability of TAM16 in mouse plasma was assessed in the same manner as that described for HLMs.

#### Assessment of glucuronide metabolites

Incubations were performed with addition of 5 μL TAM16 or positive control propranolol (from a stock solution of 0.5 M) to 450 μL of HLMs (1.11 mg/mL) in 0.05 M phosphate buffer (pH 7.4) containing 45 μL of cofactor uridine di-phosphoglucuronic acid (UDPGA) or UDPGA/NADPH added to wells of a 96 deep-well (2 mL) incubation plate. The samples were incubated at 37°C and 50 μL aliquots from incubations were removed at 0, 60 and 120 min after addition of the test compounds for analysis by UPLC-MS/MS. Chromatography was performed on a Waters BEH C18 (50 × 2.1mm 1.7μm) column heated at 40°C with a flow of 0.5 mL/min and an injection volume of 2 μL. Processing of samples was performed either by MetaboLynx software (Waters), setting each compound at t = 0 min to Control, or by manual processing of the data. Initially, the positive control (propranolol) was analyzed to ensure that glucuronide conjugates were formed.

#### Safety profiling

##### Cytochromes P450 (CYP) inhibition assays

CYP inhibition potential of TAM16 toward the major human isoforms CYP1A2, CYP2C9, CYP2C19, CYP2D6 and CYP3A4 was determined using individually expressed CYP enzymes and known fluorescent probe substrates. Human EasyCYP bactosomes (1 nmol/mL, 10 mg/mL) derived from *E.coli* were supplied by Cypex. Incubation mix (220 μL) containing expressed CYP enzyme and probe substrate in 0.05M potassium phosphate buffer (pH 7.4) was added to each well of 96-well plate and place onto a thermomixer kept at 37°C. To this incubation plate, 5 μL of the serial dilution of the compounds was added, mixed well and the plate was incubated for at least 5 min at 37°C. Miconazole was included as the positive control. After incubation, 25 μL of NADPH regenerating system solution was added to each well of the incubation plate and measurement of fluorescent product was started immediately using the BMG Pherastar spectrophotometer with appropriate parameters (Table S6). Each well was measured for production of fluorescent metabolite every min for 10 min. The data analysis was done using Xcel Fit to calculate the rate of fluorescence units per min as a % of the average rate of the solvent control wells. The % of solvent control values were then plotted against the concentration range and IC_50_ value were determined by nonlinear regression fitting of the following 4 parameter logistic equation,

$$y=Range/\left[1+{\left(x/IC_{50}\right)}^{s}\right]+Background$$

##### hERG activity assessment

The potential of TAM16 to inhibit the human cardiac hERG/IKr potassium channel functional activity was measured in an inducible hERG T-RExTM-CHO Cell line (ThermoFisher) using thallium influx as a surrogate indicator of potassium ion channel activity (Schmalhofer et al., 2010) at AbbVie, USA. Thallium enhances the fluorescent signal of BTC-AM dye (ThermoFisher). Cells were loaded with the dye for 90 min in a low potassium buffer, dye removed and compound added to the cells in a high potassium buffer in a 6 pt. dose response. After 30 min incubation with compound, channel activity was recorded upon addition of thallium buffer using a Tetra plate reader. The slope of the kinetic read was used to calculate channel activity.

##### Broad panel activity screening

Safety profiling of TAM16 for assessment of off-target interactions was performed at AbbVie, USA. The compound was evaluated across a panel of 21 liability targets (39 functional assays) which included cell based GPCRs and ion channels in both agonist and antagonist readout, and biochemical functional assays for nuclear hormone receptors and phosphodiesterases. The IC_50_ results were greater than 10 μM for all assays.

##### Ames mutagenicity assay

The two-strain Ames assay was a six-well modification of the standard Ames Petri plate incorporation test, reducing the volumes of reagents for the smaller size well. Salmonella strains TA98 and TA100 were exposed up to 1000 μg/well under conditions with and without Aroclor 1254-induced rat liver S9.

#### Chemical synthesis

##### Materials and general methods

TAM1 and its structural analogs TAM2-6 were purchased from a commercial vendor (ChemBridge Corporation, San Diego, CA). For the synthesis of analogs TAM7-24, all reagents and solvents used were reagent, analytical or HPLC grade. Anhydrous acetonitrile, diethyl ether, dichloromethane, benzene, toluene and *N*,*N*-dimethylformamide (DMF), dimethyl sulfoxide (DMSO), tetrahydrofuran (THF) and all other solvents and reagents were purchased from commercial suppliers (Sigma-Aldrich, EMD,AK Scientific, Alfa Aesar, Acros Organics, Combiblocks, Oakwoods, etc.). Thin-layer chromatography (TLC) was performed on E. Merck silica gel 60-F-254 plates and spots were visualized with UV light. Flash column chromatography was performed using 230–400 mesh silica gels (VWR). ^1^H NMR) and ^13^C NMR spectra were recorded in DMSO-*d*_*6*_, CD_3_OD and CDCl_3_ solutions on a Bruker Avance spectrometer, operating at 400 MHz for ^1^H NMR and 100 MHz for ^13^C NMR. In every case trimethylsilane (TMS) was used as an internal standard. Chemical shifts are reported in parts per million (ppm, δ units) relative to TMS solvent signal, and coupling constant (*J*) values are reported in Hertz (Hz). Liquid chromatograph mass spectra (LCMS) were obtained using SHIMADZU2010 EV using methanol solvent. Analytical reversed-phase high performance liquid chromatography (HPLC) was performed on a Waters Separation Module 2695 system equipped with an auto sampler and a Waters 996 photodiode array detector. Purity of the final compounds was determined using chromatographic systems; column, Princeton SPHER- 100, C18 (particle size = 5 μm, pore size = 10 nm, dimensions = 50 mm x 4.6 mm); mobile phase A, water; mobile phase B, acetonitrile with 0.1% formic acid and 0.1% ammonium formate. Using a flow rate of 1.0 mL/min, gradient elution was performed from 20% B to 80% B over 10 min. In every case, 10 *μ*L of a 1 mM solution was injected.

##### Synthesis of TAM8 and TAM11

**Figure:**
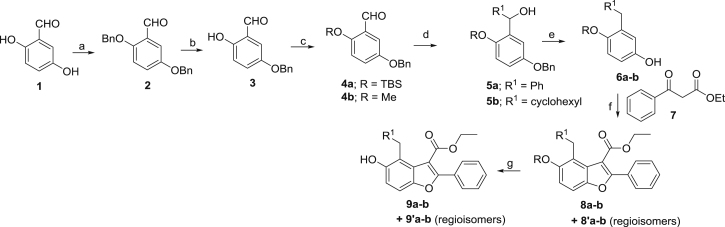

**Scheme 1**: *Reagents and conditions:* (a) BnBr, K_2_CO_3_, CH_3_CN, reflux, 8 h; (b) MgBr_2_, benzene/ether, reflux, 6 h; (c) TBSCl, imidazole, DMF, 0°C-rt, 2 hr (for 3a) and MeI, K_2_CO_3_, reflux, 8 hr (for 3b); (d) phenylmagnesium bromide or cyclohexylmagnesium bromide, THF, 2 h; (e) H_2_/Pd-C, rt, 10 h; (f) FeCl_3_, (*t*-BuO)_2_, DCE, 100°C, 2 h; (g) TBAF, THF, 0°C, 1 hr (for 9a) and BBr_3_, CH_2_Cl_2_, - 78°C-rt, 3 hr (for 9b).

Following the literature procedures (Barrero et al., 2006, Hayashi et al., 2008, Panda et al., 2007), the requisite phenyl and cyclohexyl substituted methanols **5a–b** were synthesized by the reaction between corresponding Grignard reagents and carbaldehydes **4a–b**, which in turn were easily generated from the phenol derivative 5-(benzyloxy)-2-hydroxybenzaldehyde **3**. Intermediate **3** was readily synthesized from commercially available **1** in two steps. Removal of the hydroxyl group and debenzylation of **5a–b** were accomplished with hydrogen gas under Pd-catalysis to afford the desired intermediates **6a-b** (Scheme 1).

2,5-bis(benzyloxy)benzaldehyde **(2):** To a solution of 2,5-dihydroxybenzaldehyde **1** (2.00 g, 14.48 mmol) in acetonitrile (40 mL), benzyl bromide (4.30 mL, 36.20 mmol) and K_2_CO_3_ (5.00 g, 36.20 mmol) were added. The mixture was stirred at reflux for 8 hr. After completion of the reaction, mixture was cooled to room temperature, water was added (50 mL), and extracted with EtOAc. The extract was washed with saturated solution of NH_4_Cl and brine, then dried over Na_2_SO_4_ and concentrated in vacuo. The crude residue was purified by a silica gel column chromatography with hexane and EtOAc (9:1) to give **2** (3.90 g, yield 85%) as a pale yellow solid. ^1^H NMR (400 MHz, CDCl_3_) δ 10.55 (s, 1H), 7.49 - 7.36 (m, 11 H), 7.23 - 7.20 (m, 1 H), 7.03 (d, *J* = 9.08 Hz, 1 H), 5.17 (s, 2 H), 5.08 (s, 2 H); ^13^C NMR (100 MHz, CDCl_3_) δ 189.37, 155.99, 153.09, 136.68, 136.30, 128.73 (2C), 128.61 (2C), 128.27, 128.08, 127.57 (2C), 127.37 (2C), 125.66, 124.14, 115.15, 111.85, 71.32, 70.67; MS (LCMS): *m/z* 317.30 (M-H)^+^.

5-(benzyloxy)-2-hydroxybenzaldehyde **(3):** To a suspension of 2,5-*bis*(benzyloxy)benzaldehyde **2** (3.50 g, 10.99 mmol) in a mixture of benzene and diethyl ether (7:1, 50 mL) was added magnesium bromide (2.43 g, 13.19 mmol) and stirred under refluxed for 6 hr. After cooling to room temperature, 1 N HCl was added to the resulting mixture, and extracted with EtOAc. The organic layer was washed with solution of saturated Na_2_CO_3_ and brine, and dried (Na_2_SO_4_). The solvent was evaporated in vacuo and the residue was purified by a silica gel column chromatography with hexane/EtOAc (9:1) to give the desired product **3** (2.06 g, yield 82%) as a yellow powder. ^1^H NMR (400 MHz, CDCl_3_) δ 10.69 (s, 1H), 10.69 (s, 1 H), 7.47 - 7.34 (m, 5 H), 7.26 - 7.22 (m, 1 H), 7.10 (d, *J* = 3.08 Hz, 1 H), 6.96 (d, *J* = 9.04 Hz, 1 H), 5.08 (s, 2 H), 5.08 (s, 2 H); ^13^C NMR (100 MHz, CDCl_3_) δ 196.06, 156.28, 151.84, 136.59, 128.68 (2C), 128.18, 127.47 (2C), 126.09, 120.13, 118.75, 116.96, 71.03; MS (LCMS): *m/z* 227.30 (M-H)^+^.

5-(benzyloxy)-2-((tert-butyldimethylsilyl)oxy)benzaldehyde **(4a):** To a solution of **3** (2.0 g, 8.76 mmol) in 20 mL of dichloromethane under nitrogen was added imidazole (1.0 g, 14.69 mmol) and TBSCl (1.98 g, 13.14 mmol). The resulting mixture was stirred at room temperature for 2 hr, and then extracted with EtOAc and washed with water, 1 N HCl, saturated NaHCO_3_ and brine. The crude product was purified by silica gel chromatography (hexane/EtOAc, 5:1) to afford 2.34 g (78% yield) of **4a** as a yellow oil. ^1^H NMR (400 MHz, CDCl_3_) δ 10.45 (s, 1 H), 7.52 - 7.32 (m, 6 H), 7.16 (dd, *J* = 8.90, 3.30 Hz, 1 H), 6.86 (d, *J* = 8.80 Hz, 1 H), 5.07 (s, 2 H), 1.05 (s, 9 H), 0.28 (s, 6 H); ^13^C NMR (100 MHz, CDCl_3_) δ 189.78, 153.47, 153.24, 136.69, 128.59 (2C), 128.05, 127.57 (2C), 127.20, 124.54, 121.60, 110.99, 70.61, 25.70 (3C), 18.34, 4.34; MS (LCMS): *m/z* 342.87 (M)^+^.

5-(benzyloxy)-2-methoxybenzaldehyde **(4b):** To a solution of **3** (1.0 g, 4.38 mmol) in 12 mL of acetone was added MeI (0.33 mL, 5.26 mmol) and K_2_CO_3_ (0.91 g, 6.57 mmol). Then the mixture was stirred under reflux for 8 hr. After cooling to room temperature, water was added and extracted with EtOAc, and washed with 1 N HCl, brine, dried (Na_2_SO_4_) and concentrated. The crude product was purified by silica gel chromatography (hexane/EtOAc, 6:1) to afford 0.86 g (81% yield) of **4a** as a yellow oil. ^1^H NMR (400 MHz, CDCl_3_) δ 10.47 (s, 1 H), 7.46 - 7.34 (m, 6 H), 7.24 - 7.21 (m, 1 H), 6.96 (d, *J* = 9.08 Hz, 1 H), 5.08 (s, 2 H), 3.91 (s, 3 H); ^13^C NMR (100 MHz, CDCl_3_) δ 189.42, 156.85, 152.78, 136.70, 128.58 (2C), 128.05, 127.54 (2C), 125.06, 124.18, 113.37, 112.00, 70.51, 56.15; MS (LCMS): *m/z* 242.88 (M)^+^.

(5-(benzyloxy)-2-((*tert*-butyldimethylsilyl)oxy)phenyl)(phenyl)methanol **(5a):** To a solution of 5-(benzyloxy)-2-((tert-butyldimethylsilyl)oxy)benzaldehyde **4a** (2.0 g, 5.84 mmol) in dry THF (30 mL) under nitrogen was added phenyl magnesium bromide 7.00 mL (1 M) at room temperature and stirred for 2 hr. The reaction mixture was quenched by addition of saturated NH_4_Cl (2 mL) and THF was removed in vacuo. The residue was extracted thrice with ethyl acetate, washed with brine and dried over Na_2_SO_4_. Organic extract was concentrated and purified by silica gel column chromatography with 10% ethyl acetate in hexane to furnish carbinol product **5a** (1.76 g, 72%) as a colorless viscous liquid. ^1^H NMR (400 MHz, CDCl_3_) δ 7.45 −7.29 (m, 10 H), 6.89 (d, *J* = 2.50 Hz, 1 H), 6.83 - 6.75 (m, 2 H), 6.08 (s, 1 H), 4.98 (s, 2 H), 0.96 (s, 9 H), 0.22 (d, *J* = 2.90 Hz, 6 H); ^13^C NMR (100 MHz, CDCl_3_) δ 153.09, 147.07, 142.89, 137.17, 134.66, 128.50 (2C), 128.23 (2C), 127.85, 127.55, 127.31, 126.30, 118.93, 115.01, 114.53, 71.74, 70.59, 25.77 (3C), 18.19, 1.01, −4.13; MS (LCMS): *m/z* 319.50 (M-H)^+^.

(5-(benzyloxy)-2-methoxyphenyl)(cyclohexyl)methanol **(5b):** The title compound was obtained from **4b** and cyclohexylmagnesium bromide following the procedure for compound **5a** in 66% yield after flash-chromatography (1:10 EtOAc/hexane) as a colorless viscous liquid. ^1^H NMR (400 MHz, CDCl_3_) δ 7.52 - 7.30 (m, 5 H), 6.95 (d, *J* = 2.79 Hz, 1 H), 6.90 - 6.77 (m, 2 H), 5.06 (s, 2 H), 4.55 (t, *J* = 6.90 Hz, 1 H), 3.82 (s, 3 H), 2.57 (d, *J* = 6.31 Hz, 1 H), 2.13-1.97 (m, 1 H), 1.87 - 1.58 - (m, 4 H), 1.48 - 1.08 (m, 5 H); MS (LCMS): m/z 309.05 (M-OH), 227.10 (M-C_7_H_7_)^+^.

(2-benzyl-4-(benzyloxy)phenoxy)(tert-butyl)dimethylsilane **(6a):** The compound **5a** (1.60 g, 3.80 mmol) was hydrogenated over 10% Pd/C (0.20 g) and after usual work-up and purification furnished **6a** (0.87 g, 73%) as white semi-solid. ^1^H NMR (400 MHz, CDCl_3_) δ 7.35 - 7.27 (m, 2 H), 7.26 - 7.18 (m, 3 H), 6.73 (d, *J* = 8.70 Hz, 1 H), 6.59 (dd, *J* = 8.70, 3.10 Hz, 1 H), 6.48 (d, *J* = 3.10 Hz, 1 H), 4.51 (bs, 1 H), 3.96 (s, 2 H), 1.01 (s, 9 H), 0.24 (s, 6 H); ^13^C NMR (100 MHz, CDCl_3_) δ 149.45, 147.34, 140.61, 132.71, 129.06 (2C), 128.35 (2C), 125.94, 119.12, 117.44, 113.48, 36.01, 25.83 (3C), 18.25, −4.04 (2C); MS (LCMS): *m/z* 315.25 (M)^+^.

3-(cyclohexylmethyl)-4-methoxyphenol **(6b):** The title compound was obtained from **5b** following the procedure for compound **6a** in 67% yield after flash-chromatography (1:10 EtOAc/hexane) as a colorless viscous liquid. ^1^H NMR (400 MHz, CDCl_3_) δ 6.78 - 6.71 (m, 1 H), 6.69 - 6.61 (m, 2 H), 5.12 (bs, 1 H), 3.79 (s, 3 H), 2.47 (d, *J* = 7.00 Hz, 2 H), 1.78 - 1.49 (m, 6 H), 1.32 - 1.11 (m, 6 H), 1.06 - 0.89 (m, 2 H); ^13^C NMR (100 MHz, CDCl_3_) δ 152.08, 148.89, 131.46, 118.10, 112.76, 111.90, 56.21, 38.27, 37.90, 33.29 (2C), 26.64, 26.37 (2C); MS (LCMS): *m/z* 220.73 (M)^+^.

Ethyl 4-benzyl-5-((tert-butyldimethylsilyl)oxy)-2-phenylbenzofuran-3-carboxylate **(8a)** (Guo et al., 2009): To a mixture of ethyl benzoylacetate **7** (0.14 mL, 0.83 mmol), phenol derivative **6a** (0.80 g, 2.54 mmol), and FeCl_3_·6H_2_O (0.035 g, 0.13 mmol) 1,2-dichloroethane (3.0 mL) was added under nitrogen at room temperature. Then di-tert-butyl peroxide (0.34 mL, 1.86 mmol) was added dropwise to the reaction mixture and was stirred for 2 hr at 100°C. After completion of the reaction, mixture was cooled to room temperature and was quenched with saturated NaHCO_3_ and extracted with 25 mL of ethyl acetate. The organic phase was washed with 10 mL of saturated NaHCO_3_ and 10 mL of water. The extract was dried over Na_2_SO_4_ and concentrated. The crude mixture was filtered through a small silica gel column and used as such in the next step.

***Ethyl 4-benzyl-5-hydroxy-2-phenylbenzofuran-3-carboxylate* (9a) (TAM8):** To a solution of crude mixture of **8a** (0.80 g) in dry THF (25 mL) at 0°C was added tetra-n-butylammonium fluoride (TBAF) 2.0 mL (1M in THF). After being stirred at 0°C for 1 hr, the reaction mixture was diluted with ethyl acetate, washed with water and brine. The combined organic extract was dried over Na_2_SO_4_, filtered, and concentrated under reduced pressure. Purification of the crude reaction mixture by silica gel column chromatography with ethyl acetate and hexane (1:10) as an eluent provided the desired product **9a** as a colorless viscous liquid (10% overall yield in two steps) with its regioisomer as major product. ^1^H NMR (400 MHz, CDCl_3_) δ 8.05 - 7.93 (m, 2 H), 7.56 - 7.44 (m, 4 H), 7.40 - 7.19 (m, 6 H), 4.98 (bs, 1 H), 4.42 (q, *J* = 7.00 Hz, 2 H), 4.14 (s, 2 H), 1.41 (t, *J* = 7.00 Hz, 3 H); ^13^C NMR (100 MHz, CDCl_3_) δ 164.07, 161.07, 150.89, 149.06, 139.79, 130.08, 129.81, 129.42 (3C), 128.80 (3C), 128.66 (2C), 127.98 (2C), 126.41 (2C), 112.53, 107.76, 60.61, 36.76, 14.26; MS (LCMS): *m/z* 373.10 (M)^+^.

Ethyl 6-benzyl-5-hydroxy-2-phenylbenzofuran-3-carboxylate **(9’a):** 1H NMR (400 MHz, CDCl_3_) δ 7.85 - 7.82 (m, 2 H), 7.62 (s, 1 H), 7.54 - 7.49 (m, 3 H), 7.37 - 7.22 (m, 6 H), 4.90 (bs, 1 H), 4.51 (q, *J* = 7.16 Hz, 2 H), 4.43 (s, 1 H), 4.17 (s, 1 H), 1.29 (t, *J* = 7.16 Hz, 3 H); MS (LCMS): m/z 373.09 (M)+.

Ethyl 4-(cyclohexylmethyl)-5-methoxy-2-phenylbenzofuran-3-carboxylat**e (8b).** The title compound was obtained from **6b** and **7** following procedure for compound **8a** in 17% yield after flash-chromatography (1:10 EtOAc/hexane) as a colorless viscous liquid. ^1^H NMR (400 MHz, CDCl_3_) δ 8.01 (dd, *J* = 7.30, 2.20 Hz, 2 H), 7.55 - 7.43 (m, 4 H), 7.32 - 7.22 (m, 1 H), 4.42 (q, *J* = 7.00 Hz, 2 H), 3.93 (s, 3 H), 2.63 (d, *J* = 6.9 Hz, 2 H), 1.78 −1.60 (m, 6 H), 1.42 (t, *J* = 6.99 Hz, 3 H), 1.35 - 1.14 (m, 3 H), 1.09 - 0.95 (m, 2 H); ^13^C NMR (100 MHz, CDCl_3_) δ 164.18, 160.17, 155.23, 148.55, 130.06, 129.87 (2C), 129.38 (2C), 128.96, 127.95 (2C), 120.77, 112.59, 102.72, 60.43, 55.81, 38.55, 38.37, 33.29 (2C), 26.64, 26.37 (2C), 14.21; MS (LCMS): m/z 393.08 (M)^+^.

Ethyl 6-(cyclohexylmethyl)-5-methoxy-2-phenylbenzofuran-3-carboxylate **(8’b):** 1H NMR (400 MHz, CDCl_3_) δ 7.88 - 7.86 (m, 2 H), 7.69 (s, 1 H), 7.56 - 7.49 (m, 2 H), 7.37 −7.33 (m, 2 H), 4.51 (q, *J* = 7.12 Hz, 2 H), 2.96 (d, *J* = 6.96 Hz, 1 H), 2.67 (d, *J* = 6.88 Hz, 1 H), 1.80 - 1.65 (m, 6 H), 1.35 – 0.93 (m, 8 H); MS (LCMS): m/z 393.08 (M)+.

***Ethyl 4-(cyclohexylmethyl)-5-hydroxy-2-phenylbenzofuran-3-carboxylate* (9b) (TAM11):** To a solution of compound **8b** (0.05 g, 0.13 mmol) in dichloromethane (10 mL) stirred under N_2_ gas, was added boron tribromide (0.04 mL, 0.39 mmol) at −78°C and warm to room temperature and stirred for 3 hr. After completion of the reaction, reaction was quenched by addition of water and extracted with ethyl acetate, and solvent was evaporated under reduced pressure. Crude reaction mixture was purified by silica gel column chromatography with ethyl acetate/hexane (1:10) as an eluent to provide the desired product **9b** 0.029 g, 61% yield as a colorless viscous liquid. ^1^H NMR (400 MHz, CDCl_3_) δ 8.07 - 7.94 (m, 2 H), 7.57 - 7.42 (m, 4 H), 7.29 −7.25 (m, 1 H), 5.32 (bs, 1 H), 4.41 (q, *J* = 7.20 Hz, 2 H), 2.64 (d, *J* = 7.00 Hz, 2 H), 1.82 - 1.59 (m, 6 H), 1.41 (t, *J* = 7.20 Hz, 3 H), 1.29 - 1.16 (m, 3 H), 1.11 - 0.97 (m, 2 H); ^13^C NMR (100 MHz, CDCl_3_) δ 164.18, 160.78, 151.01, 148.92, 130.00, 129.93 (2C), 129.42, 127.96 (2C), 126.45, 125.81, 112.68, 108.67, 107.20, 60.57, 38.55, 33.29 (2C), 29.88, 26.54, 26.31 (2C), 14.26; MS (LCMS): *m/z* 379.09 (M)^+^.

Ethyl 6-(cyclohexylmethyl)-5-hydroxy-2-phenylbenzofuran-3-carboxylate **(9’b):** 1H NMR (400 MHz, CDCl_3_) δ 7.89 - 7.87 (m, 2 H), 7.58 - 7.48 (m, 3 H), 7.36 (s, 1 H), 7.31 (s, 1 H), 5.14 (bs, 1 H), 4.53 (q, *J* = 7.16 Hz, 2 H), 2.95 (d, *J* = 6.96 Hz, 1 H), 2.67 (d, *J* = 7.00 Hz, 1 H), 1.78 - 1.67 (m, 6 H), 1.36 (t, *J* = 7.16 Hz, 3 H), 1.36 - 1.11 (m, 3 H), 0.92 - 0.86 (m, 2 H); MS (LCMS): m/z 379.08 (M)+.

##### **Synthesis of TAM7 and TAM9-16** (Scheme 2)

**Figure:**
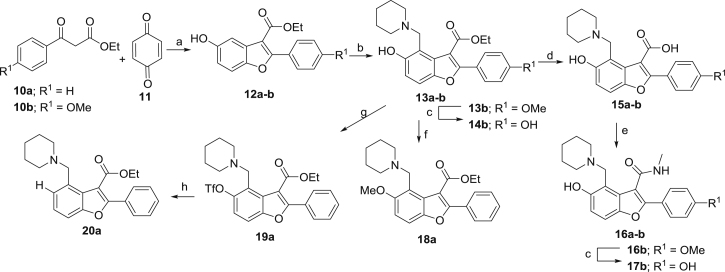

**Scheme 2:** *Reagents and conditions:* (a) Cu(OTf)_2_, toluene, reflux, 12 h; (b) formaldehyde, piperidine, reflux, 8 h; (c) BBr_3_, CH_2_Cl_2_, −78 to 0°C, 3 h; (d) NaOH, EtOH, H_2_O, reflux, 4 h; (e) i) (COCl)_2_, CH_2_Cl_2_, DMF (cat.), 0°C - rt, 3 hr, ii) methylamine, THF; (f) PPh_3_, DEAD, MeOH, overnight; (g) Tf_2_O, pyridine, rt, 1 h; (h) Pd(OAc)_2_, PPh_3_, formic acid, Et_3_N, 90°C, 8 hr.

***Ethyl 5-hydroxy-2-phenylbenzofuran-3-carboxylate* (12a)** (Mothe et al., 2010) **(TAM7)**: To a suspension of ethyl 3-oxo-3-phenylpropanoate **10a** (2.40 mL, 13.88 mmol) and Cu(OTf)_2_ (5 mol %, 0.17 g, 0.46 mmol) in dry toluene (10 mL) under a nitrogen atmosphere was added drop wise a solution of *p*-benzoquinone **11** (1.0 g, 9.25 mmol) dissolved in toluene (8 mL). The reaction mixture was stirred at reflux for 12 hr. After completion of reaction, the reaction mixture was quenched with 10 mL of saturated NH_4_Cl solution and extracted with ethyl acetate (3x10 mL). The combined organic layer was washed with brine, dried over anhydrous Na_2_SO_4_ and concentrated under reduced pressure. Crude reaction mixture was purified by flash silica gel column chromatography (1:10 ethyl acetate/hexane) to afford the compound **13a,** 1.07 g, 41% yield as a pale brown amorphous solid. ^1^H NMR (400 MHz, DMSO-*d6*) δ 9.45 (s, 1 H), 7.94 (dd, *J* = 6.68, 3.01 Hz, 2 H), 7.61 - 7.46 (m, 4 H), 7.39 (d, *J* = 2.49 Hz, 1 H), 6.87 (dd, *J* = 8.80, 2.49 Hz, 1 H), 4.32 (q, *J* = 7.12 Hz, 2 H), 1.32 (t, *J* = 7.12 Hz, 3 H),; ^13^C NMR (100 MHz, DMSO-*d6*) δ 163.55, 160.71, 154.91, 147.94, 130.76, 129.67, 129.64 (2C), 128.60 (2C), 127.83, 114.87, 112.16, 108.83, 107.03, 60.79, 14.44; MS (LCMS): *m/z* 282.93 (M)^+^.

Ethyl 5-hydroxy-2-(4-methoxyphenyl)benzofuran-3-carboxylate **(12b)**: The title compound was obtained from **10b** and *p*-benzoquinone **11** following procedure for compound **12a** in 43% yield after flash-chromatography (1:10 EtOAc/hexane) as a pale gray amorphous solid. ^1^H NMR (400 MHz, DMSO-*d6*) δ 9.39 (s, 1 H), 7.94 (d, *J* = 8.84 Hz, 2 H), 7.45 (d, *J* = 8.80 Hz, 1 H), 7.35 (d, *J* = 2.32 Hz, 1 H), 7.07 (d, *J* = 8.84 Hz, 2 H), 6.85 - 6.80 (m, 1 H), 4.32 (q, *J* = 7.08 Hz, 2 H), 3.85 (s, 3 H), 1.33 (t, *J* = 7.08 Hz, 3 H); ^13^C NMR (100 MHz, DMSO-*d6*) δ 163.75, 161.33, 160.98, 154.81, 147.61, 131.30 (2C), 128.04, 121.94, 114.37, 114.11 (2C), 111.93, 107.58, 107.05, 60.67, 55.82 14.51; MS (LCMS): *m/z* 313.00 (M)^+^.

***Ethyl 5-hydroxy-2-phenyl-4-(piperidin-1-ylmethyl)benzofuran-3-carboxylate* (13a)** (Matsumoto et al., 2005) **(TAM9):** To a solution of **12a** (1.0 g, 3.54 mmol) in EtOH (6 mL) was added formalin (37%, 1.2 eq.) and piperidine (0.35 mL, 3.54 mmol). The reaction mixture was stirred for approximately 8 hr at 80°C. The solution was then cooled to room temperature and diluted with water, and extracted with dichloromethane. The organic layer was dried over Na_2_SO_4_ and concentrated in vacuo. Crude residue was purified by silica gel column chromatography (40% ethyl acetate in n-hexane) and provided the desired product **13a** (0.97 g, 72% yield). ^1^H NMR (400 MHz, DMSO-*d6*) δ 7.77 (dd, *J* = 7.70, 1.69 Hz, 2 H), 7.53 - 7.41 (m, 3 H), 7.33 (d, *J* = 8.80 Hz, 1 H), 6.88 (d, *J* = 8.80 Hz, 1 H), 4.38 (q, 7.20 Hz, 2 H), 3.99 (s, 2 H), 2.63 - 2.45 (m, 4 H), 1.78 - 1.43 (m, 6 H), 1.30 (t, 7.20 Hz, 3 H); ^13^C NMR (100 MHz, DMSO-*d6*) δ 166.35, 156.70, 155.50, 148.16, 130.03, 129.49, 128.33 (2C), 127.87 (2C), 125.49, 115.21, 112.24, 110.67, 110.09, 61.41, 57.98, 53.93 (2C), 25.85 (2C), 23.98, 13.98; MS (LCMS): *m/z* 380.15 (M)^+^.

***5-hydroxy-2-phenyl-4-(piperidin-1-ylmethyl)benzofuran-3-carboxylic acid* (15a) (TAM10):** To a solution of ester derivative **13a** (0.80 g, 2.10 mmol) in EtOH (10 mL) a solution of NaOH (0.34 g, 8.43 mmol) in water (5 mL) was added and then the mixture was refluxed for 4 hr. After completion of the reaction, reaction mixture was allowed to cool to room temperature and poured into ice cold water. Neutralization with dil. HCl solution resulted in precipitation of desired product as white solid. Solid so obtained was filtered, washed with water and dried to obtain the desired product **15a** (0.43 g, 58% yield) as an amorphous white solid. ^1^H NMR (400 MHz, DMSO-*d6*) δ 7.83-7.76 (m, 2 H), 7.37 - 7.16 (m, 4 H), 6.79 (d, *J* = 9.00 Hz, 1 H), 4.01 (s, 2 H), 2.87 - 2.71 (m, 4 H), 1.58 - 1.39 (m, 6 H); ^13^C NMR (100 MHz, DMSO-*d6*) δ 168.31, 153.50, 152.37, 147.64, 130.92, 129.05, 128.70 (2C), 127.97, 127.53 (2C), 119.28, 113.86, 113.27, 108.18, 51.34 (2C), 50.94, 23.81 (2C), 22.21; MS (LCMS): *m/z* 352.10 (M)^+^.

***5-hydroxy-N-methyl-2-phenyl-4-(piperidin-1-ylmethyl)benzofuran-3-carboxamide* (16a) (TAM12):** To a solution of acid **15a** (0.25 g, 0.71 mmol) in dichloromethane (8 mL), oxalyl chloride (0.08 mL, 0.93 mmol) and *N*,*N-*dimethylformamide (2 drops) were added at 0°C and stirred at room temperature for 3 hr. The reaction mixture was evaporated under reduced pressure. The residue was redissolved in THF (10 mL) and a solution of methylamine 0.54 mL (2 M in THF) was added and the mixture was stirred for 2 hr. After completion of the reaction, solvent was evaporated. Water was added and extracted with dichloromethane, dried (Na_2_SO_4_) and concentrated. Crude reaction mixture was purified by silica gel column chromatography (1% methanol in dichloromethane) to provide the desired product **16a** (0.15 g, 57% yield) as a pale brown amorphous solid. ^1^H NMR (400 MHz, DMSO-*d*_*6*_) δ 8.82 (s, 1 H), 7.76 (dd, *J* = 7.85, 1.54 Hz, 2 H), 7.54 - 7.30 (m, 4 H), 6.89 (d, *J* = 8.80 Hz, 1 H), 3.96 (s, 2 H), 2.84 - 2.80 (m, 7 H), 1.78 - 1.59 (m, 4 H), 1.40 - 1.17 (m, 2 H); ^13^C NMR (100 MHz, DMSO-*d*_*6*_) δ 166.60, 159.43, 154.67, 154.07, 147.40, 128.74 (2C), 128.35, 120.20, 116.33, 113.73 (2C), 111.73, 111.67, 55.36 (2C), 53.05, 52.48, 26.83 (2C), 23.14, 21.76; MS (LCMS): *m/z* 364.89 (M)^+^.

Ethyl 5-hydroxy-2-(4-methoxyphenyl)-4-(piperidin-1-ylmethyl)benzofuran-3-carboxylate **(13b):** The title compound was obtained from **12b** following procedure for compound **13a** in 67% yield after flash-chromatography (1:10 EtOAc/hexane) as an amorphous gray solid. ^1^H NMR (400 MHz, DMSO-*d6*) δ 7.68 (d, *J* = 8.80 Hz, 2 H), 7.39 (d, *J* = 8.80 Hz, 1 H), 7.09 (d, *J* = 8.80 Hz, 2 H), 6.82 (d, *J* = 8.80 Hz, 1 H), 4.32 (q, *J* = 7.00 Hz, 2 H), 3.84 (s, 3 H), 3.77 (s, 2 H), 1.55-1.34 (m, 4 H), 1.26 (t, *J* = 7.00 Hz, 3 H); 13C NMR (100 MHz, DMSO-*d6*) δ 165.74, 160.89, 155.68, 154.20, 147.62, 129.33 (2C), 126.01, 121.91, 114.72 (2C), 114.65, 114.14, 110.75, 109.94, 61.60, 55.83 (2C), 55.45, 53.74, 25.87 (2C), 24.26, 14.30; MS (LCMS): *m/z* 409.87 (M)^+^.

***Ethyl 5-hydroxy-2-(4-hydroxyphenyl)-4-(piperidin-1-ylmethyl)benzofuran-3-carboxylate* (14b) (TAM13):** The title compound was obtained from **13b** following procedure for compound **9a** in 62% yield after flash-chromatography (4:10 EtOAc/hexane) as a gray amorphous solid. ^1^H NMR (400 MHz, DMSO-*d6*) δ 7.57 (d, *J* = 8.80 Hz, 2 H), 7.36 (d, *J* = 8.80 Hz, 1 H), 7.33 (d, *J* = 8.80 Hz, 1 H), 6.94 - 6.85 (m, 2 H), 6.80 (d, *J* = 8.80 Hz, 1 H), 4.31 (q, 7.20 Hz, 2 H), 3.78 (s, 2 H), 2.46 - 2.28 (m, 4 H) 1.54 - 1.36 (m, 6 H), 1.23 - 1.26 (t, 7.20 Hz, 3 H); ^13^C NMR (100 MHz, DMSO-*d6*) δ, 165.86, 159.48, 156.31, 154.23, 147.50, 129.46 (2C), 126.13, 120.38, 116.13 (2C), 114.39, 113.83, 110.71, 109.31, 61.52, 55.47, 53.70 (2C), 25.80 (2C), 24.17, 14.26; MS (LCMS): *m/z* 396.15 (M)^+^.

5-hydroxy-2-(4-methoxyphenyl)-4-(piperidin-1-ylmethyl)benzofuran-3-carboxylic acid **(15b):** The title compound was obtained from **13b** as described for **15a,** and used as such in the next step.

5-hydroxy-2-(4-methoxyphenyl)-N-methyl-4-(piperidin-1-ylmethyl)benzofuran-3-carboxamide **(16b):** The title compound was obtained from **15b** as described for **16a** in 60% yield after flash-chromatography (1% methanol in dichloromethane) as a pale brown amorphous solid. ^1^H NMR (400 MHz, DMSO-*d6*) δ 7.68 (d, *J* = 9.00 Hz, 2 H), 7.30 - 7.21 (m, 1 H), 6.97 (d, *J* = 8.8 Hz, 2 H), 6.88 (d, *J* = 8.8 Hz, 1 H), 6.37 (bs, 1 H), 3.95 (s, 2 H), 3.88 (s, 3 H), 2.98 (d, *J* = 4.80 Hz, 2 H), 2.81 - 2.53 (m, 4 H), 1.77 - 1.43 (m, 6 H); ^13^C NMR (100 MHz, DMSO-*d6*) δ 167.38, 160.53, 154.84, 153.75, 147.88, 128.41 (2C), 126.25, 122.05, 114.74, 114.28 (2C), 111.67, 111.07, 55.82, 55.34, 53.48 (2C), 26.96, 25.29 (2C), 23.48; MS (LCMS): *m/z* 395.03 (M)^+^.

***5-hydroxy-2-(4-hydroxyphenyl)-N-methyl-4-(piperidin-1-ylmethyl)benzofuran-3-carboxamide* (17b) (TAM16):** The title compound was obtained from **16b** as described for **9b** in 67% yield after flash-chromatography (2% methanol in dichloromethane) to provide the desired product **17b** as a pale brown amorphous solid. ^1^H NMR (400 MHz, DMSO-*d6*) δ 10.04 (s, 1 H), 8.80 (d, *J* = 4.69 Hz, 1 H), 7.72 - 7.44 (m, 3 H), 7.02 - 6.94 (m, 1 H), 6.93 (d, *J* = 8.66 Hz, 2 H), 4.30 (s, 2 H), 3.51 - 3.33 (m, 4 H), 2.81 (d, *J* = 4.70 Hz, 3 H), 1.79 - 1.73 (m, 4 H), 1.59 - 1.50 (m, 2 H); 13C NMR (100 MHz, DMSO-*d6*) δ 166.60, 159.43, 154.67, 154.07, 147.40, 128.74 (2C), 128.35, 120.20, 116.33, 113.73 (2C), 111.73, 111.67, 55.36 (2C), 53.05, 52.48, 26.83 (2C), 23.14, 21.76; MS (LCMS): *m/z* 380.99 (M)^+^.

***Ethyl 5-methoxy-2-phenyl-4-(piperidin-1-ylmethyl)benzofuran-3-carboxylate* (18a) (TAM14):** The title compound was obtained from **13a** and methanol following the literature procedure(Newlander et al., 1997). To a solution of **13a** (0.10 g, 0.26 mmol) in dry THF (5 mL), methanol (0.013 mL, 0.316 mmol), Ph_3_P (0.106 g, 0.39 mmol), and DEAD (0.07 mL, 0.39 mmol) were added. The reaction mixture was stirred under a nitrogen atmosphere for overnight. After completion of the reaction, mixture was concentrated and extracted with CH_2_Cl_2_ (2 × 15 mL). Organic extract was dried (Na_2_SO_4_), concentrated and purified by flash silica gel column chromatography (30% ethyl acetate–hexane) to afford the compound **18a** (0.05 g, 48% yield) as a pale yellow liquid. ^1^H NMR (400 MHz, CDCl_3_) δ 7.90 - 7.76 (m, 2 H), 7.53 - 7.34 (m, 4 H), 6.99 (d, *J* = 8.95 Hz, 1 H), 4.36 (q, *J* = 7.20 Hz, 2 H), 3.90 (s, 3 H), 3.83 (s, 2 H), 2.29 (m, 4 H), 1.54 - 1.35 (m, 6 H), 1.29 (t, *J* = 7.20 Hz, 3 H). MS (LCMS): *m/z* 395.15 (M)^+^.

Ethyl 2-phenyl-4-(piperidin-1-ylmethyl)-5-(((trifluoromethyl)sulfonyl)oxy)benzofuran-3-carboxylate **(19a):** Compound **13a** (0.10 g, 0.26 mmol) was dissolved in dry dichloromethane (3 mL) under nitrogen at room temperature followed by the addition of dry 2,6-lutidine (0.05 mL, 0.42 mmol). Trifluoromethanesulfonic anhydride (0.05 mL, 0.32 mmol) in dry dichloromethane (1 mL) was then added dropwise. The reaction mixture was stirred at room temperature for 1 hr. Water was added to the reaction mixture and extracted with CH_2_Cl_2_ (3 X 5 mL). The combined organic phase was washed with diluted HCl, water, brine and dried over Na_2_SO_4_, and concentrated. The crude reaction mixture was passed through a small of silica gel column and used as such in next step.

***Ethyl 2-phenyl-4-(piperidin-1-ylmethyl)benzofuran-3-carboxylate* (20a) (TAM15):** The title compound was obtained from **19a** following the literature procedure (Peterson et al., 1987) in 23% yield after flash-chromatography (1:6 EtOAc/hexane). In brief, to a solution of triflate **19a** (0.10 g, 0.19 mmol) in 3 mL of DMF, triphenylphosphine (2.0 mg, 0.008 mmol), palladium acetate (1.0 mg, 0.0044 mmol), triethylamine (0.08 mL, 0.58 mmol), and formic acid (0.015 mL, 0.39 mmol) were added. Then the mixture was stirred at 90 ^O^ C for 8 hr. Reaction mixture was diluted with water, extracted with ethyl acetate, dried over Na_2_SO_4_, and concentrated. The crude residue was chromatographed on silica gel with ethyl acetate-hexanes (1:10) to give 0.016 g (23%) of **20a** as a colorless viscous liquid. ^1^H NMR (400 MHz, DMSO-*d*_*6*_) δ 7.77 (d, *J* = 6.46 Hz, 2 H), 7.48 - 7.67 (m, 4 H), 7.34 (t, *J* = 7.78 Hz, 1 H), 7.21 (d, *J* = 7.34 Hz, 1 H), 4.32 (q, *J* = 7.12 Hz, 2 H), 3.65 (s, 2 H), 2.17 - 2.24 (m, 4 H), 1.28 - 1.39 (m, 6 H), 1.24 (t, *J* = 7.12 Hz, 3 H); ^13^C NMR (100 MHz, DMSO-*d*_*6*_) δ 165.02, 154.91, 154.14, 133.45, 130.39, 129.33, 129.22 (2C), 127.93 (2C), 125.60, 125.47, 125.12, 111.83, 110.95, 61.51, 61.48, 54.09 (2C), 25.86 (2C), 24.59, 14.18; MS (LCMS): *m/z* 363.93 (M)^+^.

##### **Synthesis of TAM19 and TAM20** (Scheme 3)

**Figure:**
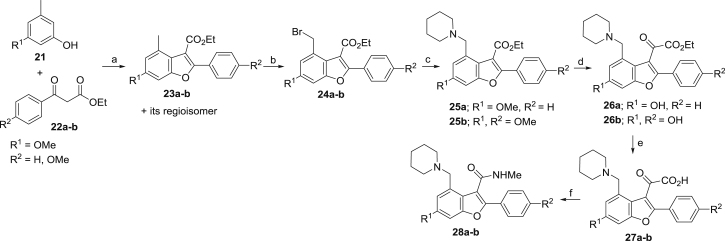

**Scheme 3**: *Reagents and conditions:* (a) FeCl_3_, (*t*-BuO)_2_, 100°C, 4 h; (b) NBS, AIBN, CCl_4_, 50°C, 5 h; (c) piperidine, NaI, K_2_CO_3_, acetone, 60°C, 6 h; (d) BBr_3_, CH_2_Cl_2_, −78 to 0°C, 3 hr, (e) NaOH, EtOH, H_2_O, 90°C, 6 h; (f) i) (COCl)_2_, CH_2_Cl_2_, DMF (cat.), 0°C - rt, 3 hr, ii) NH_2_Me in THF.

Ethyl 6-methoxy-4-methyl-2-phenylbenzofuran-3-carboxylate **(23a)**: The title compound was obtained from 3-methoxy-5-methylphenol **21** and ethyl benzoylacetate **22a** following procedure for compound **8a** in 35% yield after flash-chromatography (1:6 EtOAc/hexane) as colorless viscous liquid. ^1^H NMR (400 MHz, CDCl_3_) δ 7.87 – 7.75 (m, 2 H), 7.52 - 7.38 (m, 3 H), 6.92 (d, *J* = 1.90 Hz, 1 H), 6.75 (d, *J* = 1.30 Hz, 1 H), 4.42 (q, *J* = 7.10 Hz, 2 H), 3.87 (s, 3 H), 2.56 (s, 3 H), 1.36 (t, *J* = 7.10 Hz, 3 H); ^13^C NMR (100 MHz, CDCl_3_) δ 165.98, 158.42, 155.05, 154.74, 132.43, 130.04, 129.15, 128.38 (2C), 127.48 (2C), 119.35, 114.36, 110.61, 93.14, 61.37, 55.61, 19.90, 13.98; MS (LCMS): *m/z* 310.94 (M)^+^.

Ethyl 4-(bromomethyl)-6-methoxy-2-phenylbenzofuran-3-carboxylate **(24a)**: To a solution of ethyl 6-methoxy-4-methyl-2-phenylbenzofuran-3-carboxylate **23a** (1.0 g, 3.22 mmol) in tetrachloromethane (20 mL), *N*-bromosuccinimide (0.69 g, 3.87 mmol), and a catalytic amount of azo(bis)isobutyronitrile (AIBN) (0.053 g, 0.32 mmol) were added and refluxed for 5 hr. The solvent was removed in vacuo, and crude residue was chromatographed on silica gel with ethyl acetate-hexanes (2:10) to give of **24a** as a yellow viscous liquid. But this compound was unstable and was used as such in the next step.

Ethyl 6-methoxy-2-phenyl-4-(piperidin-1-ylmethyl)benzofuran-3-carboxylate **(25a)**: To a solution of ethyl 4-(bromomethyl)-6-methoxy-2-phenylbenzofuran-3-carboxylate **15** (0.60 g, 1.54 mmol) in acetone (10 mL), NaI (0.23 g, 1.54 mmol), piperidine (0.18 g, 1.84 mmol) and K_2_CO_3_ (0.32 g, 2.31 mmol) were added. Then the resulting mixture was refluxed for about 6 hr and then, after cooling, was filtered and the solvent was evaporated. Crude reaction mixture was purified by silica gel column chromatography and provided the desired product **16** (0.39 g, 65% yield). ^1^H NMR (400 MHz, CDCl_3_) δ 7.86 - 7.58 (m, 3 H), 7.54 - 7.38 (m, 3 H), 7.10 (d, *J* = 1.61 Hz, 1 H), 4.66 (s, 2 H), 4.30 (q, *J* = 7.09 Hz, 2 H), 3.97 (s, 3 H), 3.83 - 2.37 (m, 4 H), 2.20 - 1.51 (m, 6 H), 1.18 (t, *J* = 7.12 Hz, 3 H); MS (LCMS): *m/z* 394.03 (M)^+^.

***Ethyl 6-hydroxy-2-phenyl-4-(piperidin-1-ylmethyl)benzofuran-3-carboxylate* (26a) (TAM20):** The title compound was obtained from **25a** and boron tribromide as described for **9b** in 64% yield after flash-chromatography (1% methanol in dichloromethane)) as an amorphous brown solid. ^1^H NMR (400 MHz, CD_3_OD) δ 7.74 - 7.66 (m, 2 H), 7.57 - 7.46 (m, 3 H), 7.13 (d, *J* = 2.10 Hz, 1 H), 7.01 (d, *J* = 2.10 Hz, 1 H), 4.63 (s, 2 H), 4.31 (q, 7.10 Hz, 2 H), 3.52 - 2.25 (m, 4 H), 2.01 - 1.65 (m, 6 H), 1.15 (t, 7.10 Hz, 3 H); ^13^C NMR (100 MHz, CD_3_OD) δ 166.06, 160.47, 156.19, 156.14, 130.13, 129.83, 129.05 (2C), 127.91, 127.81 (2C), 123.07, 118.65, 109.54, 99.28, 61.34, 59.04, 52.82 (2C), 22.73 (2C), 21.48, 12.54; MS (LCMS): *m/z* 380.00 (M)^+^.

Ethyl 6-methoxy-2-(4-methoxyphenyl)-4-methylbenzofuran-3-carboxylate **(23b):** The title compound was obtained from 3-methoxy-5-methylphenol **21** and ethyl 3-(4-methoxyphenyl)-3-oxopropanoate **22b** following procedure for compound **8a** in 44% yield after flash-chromatography (1:6 EtOAc/hexane) as a colorless viscous liquid. ^1^H NMR (400 MHz, CDCl_3_) δ 7.77 (d, *J* = 8.80 Hz, 2 H), 6.99 (d, *J* = 8.95 Hz, 2 H), 6.90 (d, *J* = 1.91 Hz, 1 H), 6.73 (d, *J* = 1.32 Hz, 1 H), 4.41 (q, *J* = 7.19 Hz, 2 H), 3.88 (s, 3 H), 3.86 (s, 3 H), 2.55 (s, 3 H), 1.37 (t, *J* = 7.19 Hz, 3 H); ^13^C NMR (100 MHz, CDCl_3_) δ 166.03, 160.45, 158.14, 155.32, 154.82, 132.22, 129.18 (2C), 122.65, 119.44, 114.20, 113.85 (2C), 109.33, 93.15, 61.24, 55.60, 55.32, 20.07, 14.06; MS (LCMS): *m/z* 340.94 (M)^+^.

Ethyl 4-(bromomethyl)-6-methoxy-2-(4-methoxyphenyl)benzofuran-3-carboxylate **(24b):** The title compound was obtained from **23b** and *N*-bromosuccinimide following procedure for compound **24a** and was used as such in the next step.

Ethyl 6-methoxy-2-(4-methoxyphenyl)-4-(piperidin-1-ylmethyl)benzofuran-3-carboxylate **(25b):** The title compound was obtained from **24b** and piperidine following procedure for compound **25a** in 63% yield after flash-chromatography (1:1 EtOAc/hexane) as a colorless viscous liquid. ^1^H NMR (400 MHz, CDCl_3_) δ 7.78 (d, *J* = 8.88 Hz, 2 H), 7.02 - 6.87 (m, 3 H), 6.83 (d, *J* = 2.08 Hz, 1 H), 4.37 (q, *J* = 7.16 Hz, 2 H), 3.88 (s, 3 H), 3.86 (s, 3 H), 3.68 (s, 2 H), 2.34 - 2.26 (m, 4 H), 1.51 - 1.41 (m, 6 H), 1.34 (t, *J* = 7.16 Hz, 3 H); ^13^C NMR (100 MHz, CDCl_3_) δ 165.82, 160.35, 157.80, 155.08, 133.96, 129.25, 129.18, 122.70, 118.99, 113.90, 113.76 (2C), 113.63, 110.19, 94.07, 61.78, 61.04, 55.63, 55.31 (2C), 54.28, 25.94 (2C), 24.61, 14.97; MS (LCMS): *m/z* 423.97 (M)^+^.

Ethyl 2-(6-hydroxy-2-(4-hydroxyphenyl)-4-(piperidin-1-ylmethyl)benzofuran-3-yl)-2-oxoacetate **(26b):** The title compound was obtained from **25b** with boron tribromide as described for **9b.** Crude reaction mixture was passed through a bed of silica gel and used as such in the next step. ^1^H NMR (400 MHz, DMSO-*d6*) δ 8.90 (bs, 1 H), 7.62 - 7.47 (m, 2 H), 7.20 - 7.16 (m, 1 H), 7.04 −6.98 (m, 1 H), 6.92 (d, *J* = 8.66 Hz, 2 H), 4.65 (d, *J* = 4.11 Hz, 2 H), 4.26 (q, *J* = 7.14 Hz, 2 H), 3.46 - 3.36 (m, 2 H), 3.10 - 2.94 (m, 2 H), 1.96 - 1.59 (m, 5 H), 1.51 - 1.36 (m, 1 H), 1.15 (t, *J* = 7.14 Hz, 3 H); ^13^C NMR (100 MHz, DMSO-*d6*) δ 165.56, 159.88, 159.74, 156.02, 155.36, 131.00 (2C), 123.46, 120.51, 118.91, 115.80, 115.64 (2C), 108.52, 99.75, 61.57, 55.36, 54.07, 52.66, 22.74 (2C), 21.79, 14.05; MS (LCMS): *m/z* 423.97 (M)^+^.

6-methoxy-2-(4-methoxyphenyl)-4-(piperidin-1-ylmethyl)benzofuran-3-carboxylic acid **(27a)**: The title compound was obtained from **26a** with aqueous NaOH as described for **15a.** Crude reaction mixture was passed through a bed of silica gel and used as such in the next step.

2-(6-hydroxy-2-(4-hydroxyphenyl)-4-(piperidin-1-ylmethyl)benzofuran-3-yl)-2-oxoacetic acid **(27b):** The title compound was obtained from **26b** with aqueous NaOH as described for **15a.** Crude reaction mixture was passed through a bed of silica gel and used as such in the next step.

6-methoxy-2-(4-methoxyphenyl)-N-methyl-4-(piperidin-1-ylmethyl)benzofuran-3-carboxamide **(28a**): The title compound was obtained from **27a** following procedure for compound **16a** in 68% yield after flash-chromatography (2% methanol in dichloromethane) as a brown viscous liquid. ^1^H NMR (400 MHz, DMSO-*d6*) δ 8.94 (s, 1 H), 7.71 - 7.50 (m, 2 H), 7.46 −7.43 (m, 3 H), 7.18 - 7.14 (m, 2 H), 4.32 (s, 2 H), 2.88 - 2.83 (m, 4 H), 2.82 (d, *J* = 4.60 Hz, 3 H), 1.79 - 1.69 (m, 5 H), 1.41 - 1.36 (m, 1 H); ^13^C NMR (100 MHz, DMSO-*d6*) δ 166.20, 156.74, 155.07, 152.36, 129.65, 129.53 (2C), 129.43, 126.51 (2C), 123.10, 119.61, 112.71, 115.62, 99.53, 56.06, 52.55 (2C), 26.84 (2C), 22.86, 21.90; MS (LCMS): *m/z* 365.03(M)^+^.

***6-hydroxy-2-(4-hydroxyphenyl)-N-methyl-4-(piperidin-1-ylmethyl)benzofuran-3-carboxamide* (28b) (TAM19):** The title compound was obtained from **27b** as described for **9a** in 60% yield after flash-chromatography (2% methanol in dichloromethane) to provide the desired product **29b** as a pale yellow amorphous solid. ^1^H NMR (400 MHz, DMSO-*d6*) δ 8.36 (d, *J* = 4.21 Hz, 1 H), 7.60 - 7.49 (m, 2 H), 6.91 - 6.81 (m, 3 H), 6.75 (d, *J* = 1.61 Hz, 1 H), 3.48 (s, 2 H), 2.77 (d, *J* = 4.55 Hz, 3 H), 2.30 - 2.21 (m, 4 H), 1.55 - 1.28 (m, 6 H); ^13^C NMR (100 MHz, DMSO-*d6*) δ 166.53, 159.17, 156.21, 154.67, 152.44, 128.22 (2C), 122.49, 120.29, 119.91, 117.31, 116.32 (2C), 111.30, 99.51, 56.09, 52.42 (2C), 49.01, 26.80 (2C), 22.85, 21.94; MS (LCMS): *m/z* 380.99 (M)^+^.

##### **Synthesis of TAM17 and TAM18** (Scheme 4)

**Figure:**
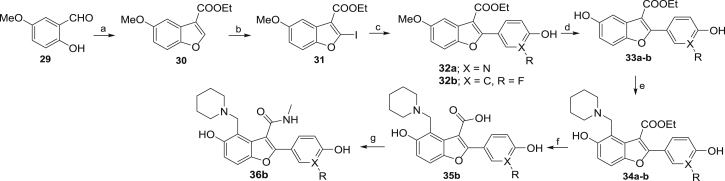

**Scheme 4:** *Reagents and conditions:* (a) N_2_CHCO_2_Et, HBF_4_-OEt_2_, rt, 2 h; (b) LDA, I_2_, −78°C, 1 h; (c) arylboronic acid, Pd(PPh_3_)_4_, Na_2_CO_3_, 100°C, 10 h; (d) BBr_3_, −78°C-rt, 4h; (e) piperidine, formaldehyde, 80°C, 3 h; (f) NaOH, EtOH-H_2_O, reflux, 4 h; (g) i) (COCl)_2_, cat. DMF, CH_2_Cl_2_, 2 hr, ii) NH_2_Me in THF.

Ethyl 5-methoxybenzofuran-3-carboxylate **(30)** (He et al., 2014): HBF_4_·Et_2_O (0.09 mL, 0.66 mmol) was added to a solution of compound **29** (1.0 g, 6.57 mmol) in CH_2_Cl_2_ (4 mL), and then a solution of ethyl diazoacetate (0.90 g, 7.89 mmol) in CH_2_Cl_2_ (4 mL) was added dropwise. Once gas evolution ceased, the reaction mixture was stirred for 2 hr, then concentrated to half, and conc. H_2_SO_4_ (0.7 mL was added to the mixture. After 30 min, the acidic mixture was neutralized with Na_2_CO_3_ (aq.). After completion of the reaction, water was added to crude mixture and extracted with CH_2_Cl_2_, and the solvent was evaporated. Crude reaction mixture was purified by silica gel column chromatography (10% ethyl acetate–hexane) and provided the desired product **30** in 1.05 g, 73% yield as a pale yellow viscous liquid. ^1^H NMR (400 MHz, DMSO-*d6*) δ 8.23 (s, 1 H), 7.55 (d, *J* = 2.60 Hz, 1 H), 7.42 (d, *J* = 8.90 Hz, 1 H), 6.97 (dd, *J* = 9.00, 2.60 Hz, 1 H), 4.43 (q, *J* = 7.10 Hz, 2 H), 3.90 (s, 3 H), 1.44 (t, *J* = 7.10 Hz, 3 H); MS (LCMS): *m/z* 220.83 (M)^+^.

Ethyl 2-iodo-5-methoxybenzofuran-3-carboxylate **(31):** To a mixture of compound **30** (1 g, 4.54 mmol) and iodine (1.44 g, 11.35 mmol) in 12 mL dry THF under N2 gas was dropwisely added LDA solution (15.90 mL, 1 M in THF) at −78°C and stirred at this temperature for 1 hr. After completion of the reaction (by LCMS) the reaction was quenched with saturated NH_4_Cl solution and concentrated. The residue was diluted with water and extracted by EtOAc. The organic extracts was washed with brine, and dried over NaSO_4_. The solvent was removed and the residue was purified by silica gel column chromatography (10% EtOAc in hexane) and afforded compound **31** (1.25 g, 80% yield) as a yellow solid. ^1^H NMR (400 MHz, DMSO-d6) δ 7.50 (d, *J* = 2.64 Hz, 1 H), 7.39 (d, *J* = 9.04 Hz, 1 H), 6.90 (dd, *J* = 9.04, 2.65 Hz, 1 H), 4.47 (q, *J* = 7.16 Hz, 2 H), 3.88 (s, 3 H), 1.37 (t, *J* = 7.16 Hz, 3 H); ^13^C NMR (100 MHz, DMSO-d6) δ 162.68, 156.93, 153.61, 126.50, 119.21, 114.05, 111.49, 108.17, 103.80, 60.61, 55.85, 14.27; MS (LCMS): *m/z* 346.31 (M)^+^.

Ethyl 2-(6-hydroxypyridin-3-yl)-5-methoxybenzofuran-3-carboxylate **(32a):** A mixture of compound **31** (0.20 g, 0.58 mmol), (6-hydroxypyridin-3-yl)boronic acid (0.096 g, 0.69 mmol) and Pd(PPh_3_)_4_ (0.033 g, 0.03 mmol) in 1 mL aq. Na_2_CO_3_ (0.12 g, 1.15 mmol) and dioxane (7 mL) under nitrogen was stirred at 100°C in a two necked round bottom flask for 10 hr. After cooling to room temperature, the reaction mixture was concentrated. The residue was dissolved in CH_2_Cl_2_ and washed with water and brine. The combined organic extracts were dried over anhydrous Na_2_SO_4_. The solvent was removed and the residue was purified by silica gel column chromatography (1:3 EtOAc/hexane**)** to give compound **32a** (0.13 g, 74%) as a colorless solid. ^1^H NMR (400 MHz, DMSO-*d6*) δ 8.37 (d, *J* = 2.50 Hz, 1 H), 7.99 (dd, *J* = 9.70, 2.50 Hz, 1 H), 7.56 (d, *J* = 8.90 Hz, 1 H), 7.44 (d, *J* = 2.50 Hz, 1 H), 6.98 (dd, *J* = 8.90, 2.60 Hz, 1 H), 6.47 (d, *J* = 9.70 Hz, 1 H), 4.35 (q, *J* = 7.20 Hz, 2 H), 3.83 (s, 3 H), 1.37 (t, *J* = 7.20 Hz, 3 H); 13C NMR (100 MHz, DMSO-*d6*) δ 163.68, 162.05, 158.67, 156.97, 148.05, 140.54, 139.33, 127.74, 119.73, 113.87, 112.22, 107.89, 107.29, 105.05, 60.96, 56.02, 14.46; MS (LCMS): *m/z* 313.80 (M)^+^.

Ethyl 5-hydroxy-2-(6-hydroxypyridin-3-yl)benzofuran-3-carboxylate **(33a):** The title compound was obtained from **32a** and boron tribromide as described for **14a** in 65% yield after flash-chromatography (1:1 EtOAc/hexane) as an amorphous white solid. ^1^H NMR (400 MHz, DMSO-*d6*) δ 8.37 (d, *J* = 2.30 Hz, 1 H), 7.96 (dd, *J* = 9.70, 2.50 Hz, 1 H), 7.42 (d, *J* = 8.80 Hz, 1 H), 7.34 (d, *J* = 2.30 Hz, 1 H), 6.81 (dd, *J* = 8.80, 2.50 Hz, 1 H), 6.43 (d, *J* = 9.50 Hz, 1 H), 4.33 (q, *J* = 7.10 Hz, 2 H), 1.36 (t, *J* = 7.10 Hz, 3 H); ^13^C NMR (100 MHz, DMSO-*d6*) δ 163.85, 162.89, 158.70, 154.92, 147.27, 140.35, 140.02, 127.83, 119.19, 114.19, 111.79, 108.02, 107.09, 106.88, 60.78, 14.53; MS (LCMS): *m/z* 299.76 (M)^+^.

***Ethyl 5-hydroxy-2-(6-hydroxypyridin-3-yl)-4-(piperidin-1-ylmethyl)benzofuran-3-carboxylate* (34a) (TAM17):** The title compound was obtained from **33a** and piperidine as described for **13a** in 74% yield after flash-chromatography (2% methanol in dichloromethane) as a colorless viscous liquid. ^1^H NMR (400 MHz, DMSO-*d6*) δ 8.38 - 7.32 (m, 1 H), 8.19 (bs, 1 H), 8.01 - 7.90 (m, 1 H), 7.74 (dd, *J* = 9.6, 2.6 Hz, 1 H), 7.45 - 7.34 (m, 1 H), 6.83 (d, *J* = 8.8 Hz, 1 H), 6.47 (d, *J* = 9.5 Hz, 1 H), 4.36 - 4.25 (m, 2 H), 3.86 (s, 2 H), 2.48 - 2.33 (m, 4 H), 1.64 - 1.35 (m, 6 H), 1.28 (t, *J* = 7.20 Hz, 3 H). MS (LCMS): *m/z* 396.96 (M)^+^.

Ethyl 2-(3-fluoro-4-hydroxyphenyl)-5-methoxybenzofuran-3-carboxylate **(32b):** The title compound was obtained from **31** and (3-fluoro-4-hydroxyphenyl)boronic acid as described for **32a** and after usual work up, crude reaction mixture was passed through a silica gel column and used as such in the next step.

Ethyl 2-(3-fluoro-4-hydroxyphenyl)-5-hydroxybenzofuran-3-carboxylate **(33b):** The title compound was obtained from **32b** and borontribromide as described for **14a** in 76% yield after flash-chromatography (1:1 EtOAc/hexane) as an amorphous white solid. ^1^H NMR (400 MHz, DMSO-*d6*) δ 7.87 (dd, *J* = 12.76, 1.91 Hz, 1 H), 7.67 (dd, *J* = 8.58, 1.25 Hz, 1 H), 7.44 (d, *J* = 8.80 Hz, 1 H), 7.36 (d, *J* = 2.35 Hz, 1 H), 7.08 (t, *J* = 8.80 Hz, 1 H), 6.83 (dd, *J* = 8.80, 2.49 Hz, 1 H), 4.33 (q, *J* = 7.04 Hz, 2 H), 1.34 (t, *J* = 7.12 Hz, 3 H); ^13^C NMR (100 MHz, DMSO-*d6*) δ 163.69, 159.83, 154.83, 151.88, 149.49, 147.54, 127.96, 126.47, 120.77, 117.83, 117.51, 114.55, 111.94, 107.80, 107.11, 60.78, 14.45; MS (LCMS): *m/z* 316.64 (M)^+^.

Ethyl 2-(3-fluoro-4-hydroxyphenyl)-5-hydroxy-4-(piperidin-1-ylmethyl)benzofuran-3-carboxylate **(34b):** The title compound was obtained from **33a** and piperidine as described for **13a** in 74% yield after flash-chromatography (1% methanol in dichloromethane) as an amorphous pale yellow solid. ^1^H NMR (400 MHz, DMSO-*d6*) δ 7.52 (dd, *J* = 12.32, 2.10 Hz, 1 H), 7.44 - 7.32 (m, 2 H), 7.14 - 7.05 (m, 1 H), 6.82 (d, *J* = 8.80 Hz, 1 H), 4.32 (q, *J* = 7.04 Hz, 2 H), 3.76 (s, 2 H), 2.46 - 2.25 (m, 4 H), 1.33 - 1.28 (m, 6 H), 1.26 (t, *J* = 7.04 Hz, 3 H); ^13^C NMR (100 MHz, DMSO-*d6*) δ 165.56, 154.78, 154.27, 152.34, 149.95, 147.48, 125.92, 124.70, 120.70, 118.47, 115.70, 114.82, 114.08, 110.77, 110.15, 61.64, 55.49, 53.73 (2C), 25.81 (2C), 24.20, 14.22; MS (LCMS): *m/z* 413.93 (M)^+^.

2-(3-fluoro-4-hydroxyphenyl)-5-hydroxy-4-(piperidin-1-ylmethyl)benzofuran-3-carboxylic acid **(35b):** The title compound was obtained from **34b** with aqueous NaOH as described for **15a.** Crude reaction mixture was passed through a bed of silica gel and used as such in the next step.

***2-(3-fluoro-4-hydroxyphenyl)-5-hydroxy-N-methyl-4-(piperidin-1-ylmethyl)benzofuran-3-carboxamide* (37b) (TAM18):** The title compound was obtained from **35b** as described for **14a** in 74% yield after flash-chromatography (2% methanol in dichloromethane) as an amorphous pale brown solid. ^1^H NMR (400 MHz, DMSO-*d6*) δ 8.64 (d, *J* = 4.7 Hz, 1 H), 7.51 - 7.32 (m, 3 H), 7.09 (t, *J* = 8.8 Hz, 1 H), 6.75 (d, *J* = 8.8 Hz, 1 H), 3.76 (s, 2 H), 2.81 (d, *J* = 4.5 Hz, 3 H), 3.50 - 2.28 (m, 4 H), 1.66 - 1.34 (m, 6 H); ^13^C NMR (100 MHz, DMSO-*d6*) δ 166.06, 154.59, 152.55, 150.79, 150.15, 147.21, 146.58, 146.46, 126.99, 123.08, 121.48, 118.76, 114.38, 113.52, 110.71, 55.91, 53.76 (2C), 26.68 (2C), 25.78, 23.95; MS (LCMS): *m/z* 398.82 (M)^+^.

##### Synthesis of TAM21-TAM24

***Ethyl 5-hydroxy-4-((3-(hydroxymethyl)piperidin-1-yl)methyl)-2-phenylbenzofuran-3-carboxylate* (TAM21):** To the solution of **12a** (0.54 g, 3.0 mmol) in EtOH (10 mL) was added formalin (37%, 1.2 eq.) and 3-piperidinemethanol (0.42 g, 3.75 mmol). The reaction mixture was stirred for approximately 8h at 80°C. The solution was then cooled to room temperature and diluted with water, and extracted with dichloromethane. The organic layer was dried over Na_2_SO_4_ and concentrated in vacuo. Crude residue was purified by silica gel column chromatography (40% ethyl acetate in n-hexane) and provided the desired product (1.11 g, 90% yield) as a white amorphous powder. ^1^H NMR (400 MHz, DMSO) δ 7.71 (dd, *J* = 7.9, 1.3 Hz, 2H), 7.58 – 7.46 (m, 3H), 7.42 (d, *J* = 8.8 Hz, 1H), 6.85 (d, *J* = 8.8 Hz, 1H), 4.32 (q, *J* = 7.1 Hz, 2H), 3.90 – 3.70 (m, 2H), 3.27 (dd, *J* = 10.6, 5.2 Hz, 1H), 3.21 – 3.08 (m, 1H), 2.88 (d, *J* = 9.8 Hz, 1H), 2.75 (d, *J* = 10.8 Hz, 1H), 1.94 (t, *J* = 10.6 Hz, 1H), 1.70 (t, *J* = 10.6 Hz, 1H), 1.61 (m, 3H), 1.36 (dd, *J* = 25.8, 13.6 Hz, 1H), 1.23 (t, *J* = 7.1 Hz, 3H), 0.91 (dd, *J* = 20.7, 11.2 Hz, 1H).^13^C NMR (101 MHz, DMSO) δ 165.04, 154.80, 153.73, 147.40, 129.73, 129.04, 128.70, 127.17, 125.31, 114.65, 113.93, 110.80, 110.42, 64.10, 61.22, 56.39, 54.74, 53.26, 38.71, 26.77, 24.48, 13.68; MS (LCMS): *m/z* 409.94 (M^+^).

***Ethyl 5-hydroxy-4-((2-(hydroxymethyl)piperidin-1-yl)methyl)-2-phenylbenzofuran-3-carboxylate* (TAM22):** To the solution of **12a** (0.28 g, 1.0 mmol) in EtOH (3 mL) was added formalin (37%, 1.2 eq.) and 2-piperidinemethanol (0.14 g, 1.25 mmol). The reaction mixture was stirred for approximately 8h at 80°C. The solution was then cooled to room temperature and diluted with water, and extracted with dichloromethane. The organic layer was dried over Na_2_SO_4_ and concentrated in vacuo. Crude residue was purified by silica gel column chromatography (40% ethyl acetate in n-hexane) and provided the desired product (0.35 g, 86% yield) as a yellowish amorphous powder. ^1^H NMR (400 MHz, DMSO) δ 7.70 (d, *J* = 7.1 Hz, 2H), 7.58 – 7.45 (m, 3H), 7.42 (d, *J* = 8.8 Hz, 1H), 6.82 (d, *J* = 8.8 Hz, 1H), 4.46 (d, *J* = 14.6 Hz, 1H), 4.34 (q, *J* = 7.0 Hz, 2H), 3.77 (d, *J* = 14.6 Hz, 1H), 3.60 (d, *J* = 4.6 Hz, 2H), 2.78 (d,*J* = 11.7 Hz, 1H), 2.45 (d, *J* = 4.3 Hz, 1H), 2.30 – 2.12 (m, 1H), 1.59 – 1.44 (m, 2H), 1.46 (dd, *J* = 21.9, 13.6 Hz, 2H), 1.44 – 1.29 (m, 2H), 1.24 (t, *J* = 7.1 Hz, 3H).^13^C NMR (101 MHz, DMSO) δ 165.51, 163.23, 154.77, 154.55, 147.20, 129.80, 128.97, 128.78, 128.43, 127.02, 125.07, 114.98, 113.11, 110.54, 110.19, 61.95, 61.52, 61.19, 51.42, 50.49, 27.25, 24.11, 22.20, 13.66; *m/z* 409. 87 (M^+^).

***Ethyl 5-hydroxy-4-((3-(hydroxymethyl)piperidin-1-yl)methyl)-2-(4-hydroxyphenyl)benzofuran-3-carboxylate*** (**TAM23**): To the solution of **12b** (0.31 g, 1.0 mmol) in EtOH (3 mL) was added formalin (37%, 1.2 eq.) and 3-piperidinemethanol (0.14 g, 1.25 mmol). The reaction mixture was stirred for approximately 8h at 80°C. The solution was then cooled to room temperature and diluted with water, and extracted with dichloromethane. The organic layer was dried over Na_2_SO_4_ and concentrated in vacuo. Crude residue was purified by silica gel column chromatography (40% ethyl acetate in n-hexane) and provided the desired product (0.28 g, 66% yield). ^1^H NMR (400 MHz, DMSO) δ 7.54 (d, *J* = 8.6 Hz, 2H), 7.35 (d, *J* = 8.8 Hz, 1H), 6.88 (d, *J* = 8.5 Hz, 2H), 6.78 (d, *J* = 8.8 Hz, 1H), 4.30 (d, *J* = 7.1 Hz, 1H), 3.94 – 3.63 (m, 2H), 3.25 (dd, *J* = 10.4, 4.9 Hz, 1H), 3.22 – 3.06 (m, 1H), 2.87 (d, *J* = 11.5 Hz, 1H), 2.74 (d, *J* = 11.1 Hz, 1H), 1.93 (t, *J* = 11.0 Hz, 1H), 1.83 – 1.48 (m, 4H), 1.36 (dd, *J* = 24.3, 9.8 Hz, 1H), 1.24 (t, *J* = 7.1 Hz, 3H), 0.90 (d, *J* = 9.7 Hz, 1H).^13^C NMR (101 MHz, DMSO) δ 165.85, 164.56, 159.56, 156.19, 154.20, 147.49, 130.05, 129.42, 126.12, 120.30, 116.05, 114.39, 114.08, 110.65, 109.32, 64.62, 61.56, 56.90, 55.45, 53.77, 39.24, 27.29, 25.00, 14.29; MS (LCMS): *m/z* 425.97 (M^+^).

***5-Hydroxy-4-((3-(hydroxymethyl)piperidin-1-yl)methyl)-N-methyl-2-phenylbenzofuran-3-carboxamide* (TAM24):** To the solution of ester derivative **TAM21** (0.82 g, 2.0 mmol) in EtOH (10 mL), a solution of NaOH (0.34 g, 8.43 mmol) in water (5 mL) was added and the resulting mixture was refluxed for 4 hr. After the completion of the reaction, reaction mixture was allowed to cool to room temperature and poured into ice cold water. Neutralization with dil. HCl solution resulted in precipitation of white acid. Acid so obtained was filtered, washed with water and dried and directly used for next step.To the solution of acid (0.38 g, 1.0 mmol) in dichloromethane (10 mL) was added oxalyl chloride (0.11 mL, 1.41 mmol) and *N,N*-dimethylformaide (2 drops) was added at 0°C and stirred at room temperature for 3h. The reaction mixture was evaporated under reduced pressure. The residue was re-dissolved in THF (10 mL) and a solution of methylamine (0.75 mL, 2.0 M in THF) was added and the mixture was stirred for 2h. After the completion of reaction, solvent was evaporated. Water was added and extracted with dichloromethane. The organic layer was dried over Na_2_SO_4_ and concentrated in vacuo. Crude residue was purified by silica gel column chromatography (1%–3% methanol in dichloromethane) to provide the desired product (0.28 g, 71% yield) as a pale yellow amorphous solid. ^1^H NMR (400 MHz, DMSO) δ 8.65 (d, *J* = 4.6 Hz, 1H), 7.77 – 7.72 (m, 2H), 7.50 (t, *J* = 7.5 Hz, 2H), 7.41 (dd, *J* = 11.8, 5.8 Hz, 2H), 6.78 (d, *J* = 8.8 Hz, 1H), 3.90 – 3.75 (m, 2H), 3.30 (dd, *J* = 10.6, 5.2 Hz, 1H), 3.19 (dd, *J* = 10.6, 7.1 Hz, 1H), 3.00 (d, *J* = 10.1 Hz, 1H), 2.94 – 2.83 (m, 1H), 2.79 s, 3H), 2.13 – 2.00 (m, 1H), 1.84 (t, *J* = 10.7 Hz, 1H), 1.67 (dd, *J* = 16.9, 6.8 Hz, 3H), 1.50 (dd, *J* = 16.1, 8.1 Hz, 1H), 0.98 (dd, *J* = 21.8, 11.7 Hz, 1H).^13^C NMR (101 MHz, DMSO) δ 166.06, 163.88, 154.67, 151.51, 147.51, 129.94, 129.57, 129.42, 126.93, 126.26, 114.83, 114.71, 112.69, 111.02, 64.42, 56.78, 55.82, 53.74, 39.11, 26.88, 26.71, 24.77; MS (LCMS): *m/z* 494.97 (M^+^).

Purity of TAM1 analogs as determined by HPLC using Water/Acetonitrile (0.1% Formic and 0.1% Ammonium Formate Acid).

**Table:** 

Compound	R_t_ (min)	Purity (%)
9a/TAM8	4.83	97.5
9b/TAM11	5.47	97.3
13a/TAM9	4.18	97.7
14b/TAM13	3.87	96.4
15a/TAM10	2.93	97.5
16a/TAM12	3.48	97.6
17b/TAM16	2.98	98.6
18a/TAM14	4.64	96.8
20a/TAM15	4.71	97.8
26a/TAM20	3.83	99.1
34a/TAM17	2.76	96.2
37b/TAM18	2.84	98.3
TAM21	3.26	97.5
TAM22	3.37	96.8
TAM23	2.76	97.8
TAM24	2.68	98.9

### Quantification and Statistical Analysis

All potency determinations and dose response curves were produced using Prism 5.0 (GraphPad Software). Animal efficacy data were analyzed using Prism 4.0 (GraphPad) and SigmaPlot 11.0 (Jandel Corporation) software. Number of samples and statistical tests used are provided in the STAR Methods.

### Data and Software Availability

Atomic coordinates and structure factors for the reported crystal structures have been deposited with the Protein Data Bank under accession codes PDB: 5V3W (Apo Pks13-TE), PDB: 5V3X (Pks13-TE:TAM1), PDB: 5V3Y (Pks13-TE:TAM16), PDB: 5V3Z (Pks13-TE(D1607N)), PDB: 5V40 (Pks13-TE:TAM6), PDB: 5V41 (Pks13-TE:TAM5), and PDB: 5V42 (Pks13-TE:TAM3).

## Author Contributions

A.A. designed and performed biochemical, whole-cell, and X-ray crystallography experiments; analyzed and interpreted data; and participated in writing the manuscript. M.K.P. designed and optimized synthetic strategies, selected commercial analogs, and contributed to manuscript writing; M.K.P. and J.C.S. designed inhibitors; N.S. performed physicochemical and PK studies; D.W. and S.G. developed animal use protocol and assisted in PK studies; E.C.B., P.K., F.A.S., and P.D.v.H. determined the MIC on drug-resistant strains; N.K.D., L.W., C.H., G.T.R., P.C.K., and A.J.L. conducted mouse efficacy studies and interpreted data; C.W., D.M., and D.F. contributed to SAR data analysis, analog design, and synthetic strategies; A.A., S.W., and E.J.R. isolated resistance mutants; O.E., S.N., J.R., and P.S. performed DMPK studies; T.S. performed THP-1 infection experiments; R.v.d.M. performed whole-genome sequencing data analysis of clinical strains from South Africa; R.S. assisted with biochemical assays and MIC determination; R.C.D. synthesized analogs; T.R.I. performed whole-genome sequencing data analysis and contributed to the writing; J.C.S. designed and supervised the study, analyzed and interpreted data, and co-wrote the manuscript. All authors discussed the results and commented on the manuscript.

## Supplemental Information

Document S1. Tables S1–S4, S6, and S7

Table S5. List of clinical *M. tuberculosis* strains tested, Related to Table 2 and Table S4 This excel file contains all of the drug-resistance conferring mutations found from whole genome sequencing in each of the clinical strains tested in this study.
