# Supplementary material for: Development of a Novel Lead that Targets M. tuberculosis Polyketide Synthase 13
Source: Cell. 2017 Jul 13;170(2):249–259.e25. doi: 10.1016/j.cell.2017.06.025 (PMC5509550; doi:10.1016/j.cell.2017.06.025)
Supplement: Document S1. Tables S1–S4, S6, and S7 [file mmc1.pdf]

Cell, Volume *170*

Supplemental Information

Development of a Novel Lead that Targets

M. tuberculosis Polyketide Synthase 13

Anup Aggarwal, Maloy K. Parai, Nishant Shetty, Deeann Wallis, Lisa Woolhiser, Courtney Hastings, Noton K. Dutta, Stacy Galaviz, Ramesh C. Dhakal, Rupesh Shrestha, Shoko Wakabayashi, Chris Walpole, David Matthews, David Floyd, Paul Scullion, Jennifer Riley, Ola Epemolu, Suzanne Norval, Thomas Snavely, Gregory T. Robertson, Eric J. Rubin, Thomas R. Ioerger, Frik A. Sirgel, Ruben van der Merwe, Paul D. van Helden, Peter Keller, Erik C. Böttger, Petros C. Karakousis, Anne J. Lenaerts, and James C. Sacchettini

Supplemental Tables

Table S1. Enzyme kinetic parameters of wild-type Pks13-TE and D1607N, D1644G mutants. Related to Figure 1.

Protein	K_m (μM)[a]	k_{cat} (min^{-1})	k_{cat}/K_m (M^{-1} min^{-1})	Relative activity	TAM1 IC_{50} (μM)
Wt.	19.5 ± 3.4	140 ± 9 $x10^{-4}$	7.2 ± 1.3 $x10^{2}$	1	0.26 ± 0.03
D1607N	8.5 ± 0.9	105 ± 3 $x10^{-4}$	12.3 ± 1.4 $x10^{2}$	1.7	0.76 ± 0.04
D1644G	6.9 ± 1.1	166 ± 6 $x10^{-4}$	24 ± 3.8 $x10^{2}$	3.3	17.4 ± 1.3

[a]Kinetics values were obtained by fitting the raw data to the Michaelis-Menten equation in Prism 5.0. IC_{50} values were obtained as described in the Methods section. Results are presented as mean ± SD.

Table S2. X-ray data collection and refinement statistics for Pks13-TE structures (Protein Data Bank ID for each structure is given in bold). Related to Figure 1.

	Apo Pks13-TE	Pks13-TE:TAM1	Pks13-TE (D1607N)
PDB ID	**5V3W**	**5V3X**	**5V3Z**
Data collection			
Space group	$P2_12_12$	$P2_12_12$	$P2_12_12$
Cell dimensions			
a, *b*, *c* (Å)	88.7, 106.9, 57.8	89.2, 109.5, 57.0	88.7, 108.9, 58.1
α, β, γ (°)	90, 90, 90	90, 90, 90	90, 90, 90
Resolution (Å)	39.25 - 1.72 (1.75 - 1.72)[a]	34.59 - 1.94 (1.98 - 1.94)	44.36 - 1.88 (1.91 - 1.88)
No. reflections measured	459445 (17486)	263793 (14486)	210572 (8342)
R_{merge} (%)	10.9 (55.4)	17.9 (140.8)	14.2 (152.0)
$I / \sigma I$	24.4 (3.9)	18.0 (1.5)	8.6 (0.5)
Completeness (%)	99.8 (99.5)	95.0 (92.9)	93.2 (60.3)
Redundancy	7.8 (6.3)	6.6 (5.3)	4.9 (2.7)
$CC_{1/2}$ (%)	99.7 (83.5)	99.4 (15.7)	99.5 (21.8)
CC*	0.999 (0.954)	0.998 (0.521)	0.999 (0.598)
Refinement			
Resolution (Å)	1.72	1.94	1.88
No. reflections	58684 (2769)	40267 (2757)	43273 (1865)
R_{work} / R_{free} (%)	16.9/20.1	19.8/24.7	18.8/23.0
No. atoms			
Protein	4227	4099	4212
Ligand/ion	23	58	36
Water	471	259	335
B-factors ($Å^2$)			
Protein	21.8	39.8	35.9
Ligand/ion	29.1	36.8	48.5
Water	30.7	42.4	41.3
R.m.s. deviations			
Bond lengths (Å)	0.010	0.008	0.011
Bond angles (°)	1.25	1.13	1.25
Ramachandran Plot			
Favored (%)	98.5	98.5	98.4
Outliers (%)	0.0	0.2	0.0

[a]Values for the highest resolution shell are shown in parentheses.
Data for each structure was collected from a single crystal.

Table S3. X-ray data collection and refinement statistics for Pks13-TE-inhibitor complex structures (Protein Data Bank ID for each structure is given in bold). Related to Figure 1.

PDB ID	Pks13-TE:TAM3 **5V42**	Pks13-TE:TAM5 **5V41**	Pks13-TE:TAM6 **5V40**	Pks13-TE:TAM16 **5V3Y**
Data collection				
Space group	$P2_12_12$	$P2_12_12$	$P2_12_12$	$P2_12_12$
Cell dimensions				
a, *b*, *c* (Å)	89.4, 109.4, 56.9	88.9, 109.7, 57.4	88.0, 109.4, 57.0	89.6, 108.8, 56.7
α, β, γ (°)	90, 90, 90	90, 90, 90	90, 90, 90	90, 90, 90
Resolution (Å)	33.76 - 1.99 (2.05 - 1.99)[a]	48.20 - 2.05 (2.11 – 2.05)	46.45 - 1.99 (2.04 - 1.99)	34.58 - 1.98 (2.03 - 1.98)
No. reflections measured	179773 (11963)	186190 (12180)	162703 (10119)	254082 (21058)
R_{merge} (%)	11.6 (122.7)	15.4 (112.3)	12.4 (97.2)	16.2 (153.9)
$I / \sigma I$	14.1 (1.4)	11.5 (1.6)	9.9 (1.2)	15.0 (2.1)
Completeness (%)	87.9 (83.3)	94.1 (91.4)	99.0 (92.8)	93.9 (97.9)
Redundancy	5.2 (4.5)	5.5 (4.5)	4.3 (4.1)	6.9 (7.2)
$CC_{1/2}$ (%)	99.7 (52.9)	99.3 (54.6)	99.6 (60.8)	99.5 (59.7)
CC*	0.999 (0.832)	0.998 (0.841)	0.999 (0.870)	0.999 (0.865)
Refinement				
Resolution (Å)	1.99	2.05	1.99	1.98
No. reflections	34456 (2660)	33814 (2701)	38093 (2497)	36892 (2924)
R_{work} / R_{free} (%)	19.4/23.3	20.2/24.2	18.5/21.7	19.4/23.9
No. atoms				
Protein	4115	4136	4188	4155
Ligand/ion	54	28	50	61
Water	247	301	398	309
B-factors ($Å^2$)				
Protein	44.3	38.9	31.6	36.3
Ligand/ion	37.4	40.5	28.3	31.0
Water	45.6	43.5	37.9	40.3
R.m.s. deviations				
Bond lengths (Å)	0.008	0.005	0.008	0.008
Bond angles (°)	1.12	0.88	1.07	1.11
Ramachandran Plot				
Favored (%)	98.9	98.9	99.0	99.0
Outliers (%)	0.0	0.0	0.0	0.0

[a]Values for the highest resolution shell are shown in parentheses.
Data for each structure was collected from a single crystal.

Table S4. TAM16 MICs for *M. tuberculosis* strains with different drug-susceptibility profiles. Related to Table 2.

Strain	Strain type	Resistance status	MGIT 960 MIC_{90}[a] (μM)
H37Rv	Lab	Susceptible	0.125-0.25
H37RvMa	Lab	Susceptible	0.125
R296	Clinical	MDR (INH,RMP,EMB,ETH)	0.06
2011 186003	Clinical	MDR (INH,RMP,SM)	0.1
2010 186016	Clinical	MDR (INH,RMP,EMB)	0.21
2011 186019	Clinical	MDR (INH,RMP,PZA,ETH,EMB,SM,CAP)	0.1
2011 186013	Clinical	MDR (INH,RMP,EMB,SM)	0.05
2012 186113	Clinical	MDR (INH,RMP)	0.21
2014 186019	Clinical	MDR (INH,RMP,PZA,SM)	0.21
2015 500170	Clinical	Poly-resistant (INH,ETH,SM)	0.42
2011 186014	Clinical	Pre-XDR (INH,RMP,EMB,SM,CAP)	0.05
2008 186033	Clinical	Pre-XDR (INH,RMP,PZA,EMB,ETH,KAN,SM)	0.42
2007 186071	Clinical	Pre-XDR (INH,RMP,PZA,EMB,ETH,KAN,SM,CAP,MOX)	0.42
2008 186034	Clinical	Pre-XDR (INH,RMP,PZA,EMB,ETH,SM,MOX)	0.42
MD52/54	Clinical	Pre-XDR (INH,RMP,PZA,EMB,OFX)	0.125
MD55/58	Clinical	XDR (INH,RMP,PZA,EMB,SM)	0.125
MD8	Clinical	XDR (INH,RMP,PZA,EMB,ETH,KAN,AM,SM,CAP,OFX)	0.125
MD96	Clinical	XDR (INH,RMP,PZA,EMB,ETH,KAN,AM,SM,CAP,OFX)	0.125
MD17	Clinical	XDR (INH,RMP,PZA,EMB,ETH,KAN,AM,SM,CAP,OFX)	0.25
MD24	Clinical	XDR (INH,RMP,PZA,EMB,ETH,KAN,AM,SM,CAP,OFX)	0.25
R88	Clinical	INH-mono	0.125
2012 500139	Clinical	INH-mono	0.1
2007 186133	Clinical	INH-mono	0.1
2007 186136	Clinical	INH-mono	0.1
2015 500051	Clinical	INH-mono	0.1
2015 180693	Clinical	INH-mono	0.05
R3027	Clinical	RMP-mono	0.125
2013 181808	Clinical	SM-mono	0.42
2013 181445	Clinical	Susceptible	0.1
2013 181605	Clinical	Susceptible	0.21
2013 181682	Clinical	Susceptible	0.1
2013 181710	Clinical	Susceptible	0.1
SAWC1125	Clinical	Susceptible	0.25
SAWC2371	Clinical	Susceptible	0.06
SAWC3200	Clinical	Susceptible	0.125
SAWC3385	Clinical	Susceptible	0.06
SAWC3388	Clinical	Susceptible	0.125
SAWC3933	Clinical	Susceptible	0.125
SAWC4046	Clinical	Susceptible	0.06
SAWC4083	Clinical	Susceptible	0.125

[a]The lowest concentration of drug that inhibited growth of more than 90% of the bacterial population was considered to be the MIC_{90}. INH, isoniazid; RMP, rifampicin; EMB, ethambutol; SM, streptomycin; PZA, pyrazinamide; OFX, ofloxacin; CAP, capreomycin; AM, amikacin; ETH, ethionamide, KAN, kanamycin; MOX, moxifloxacin.

MDR, *Mtb* strains resistant to both INH and RMP, with or without resistance to other anti-TB drugs; XDR, MDR strains that are also resistant to any fluoroquinolone, and to any of the three second-line injectables (AM, CAP, and KAN); Pre-XDR, MDR strains with additional resistance either a fluoroquinolone or an injectable, but not both.

Table S6. CYP inhibition fluorescence measurement details. Related to Table 3.

CYP Isoform	Substrate	Metabolite	Excitation (nm)	Emission (nm)	IC_{50} (μM)	
					TAM16	Miconazole[a]
CYP1A2	Ethoxyresorufin	Resorufin	540	590	>10	1.1
CYP2C9	7-methoxy-4-(trifluoromethyl)-coumarin	7-hydroxy-4-(trifluoromethyl)-coumarin	430	540	>10	>10
CYP2C19	3-Cyano-7-Ethoxycoumarin	3-Cyano-7-methoxycoumarin	405	450	>10	0.01
CYP2D6	7-methoxy-4-(aminomethyl)-coumarin	7-hydroxy-4-(aminomethyl)-coumarin (HAMC)	405	450	4.2	0.2
CYP3A4	Diethoxyfluorescein	Fluorescein	430	540	>10	0.03
CYP3A4	7-Benzyloxyquinoline	7-Hydroxyquinoloine	485	520	9.8	0.05

[a]Positive control.

Table S7. Assessment of TAM16 efficacy in chronic Balb/C model. Related to Figure 3B.

Five mice were sacrificed at day 1 post-infection to determine bacterial uptake. At Day 28 post-infection 6 mice were sacrificed to determine bacterial load in the lungs at the start of therapy. Treatment was started 4 weeks post-infection and continued for 4 and 8 weeks. In combination studies, TAM16 and Isoniazid (INH) were administered 1-hour following prior administration of rifampicin (RIF).

Group	Mouse Log$_{10}$ lung burdens							mean log$_{10}$ CFU	SEM	n[a]
	A	B	C	D	E	F	G			
Day 1 Lung	1.52	1.61	1.99	1.56	1.79			1.69	0.09	5/5
D28 pretreatment control	6.18	5.77	5.76	5.62	5.61	6.10		5.84	0.10	6/6
4 week Rx										
TAM16	4.63	4.77	4.50	4.46	4.63	4.39		4.56	0.06	6/6
RIF	4.47	3.65	4.33	3.97	4.45	3.88		4.13	0.14	6/6
TAM16+RIF	3.20	2.38	2.82	3.36	3.11	3.08		2.99	0.14	6/6
INH+RIF	3.49	3.33	3.28	3.50	3.23	2.73		3.26	0.12	6/6
Vehicle	5.41	5.17	5.74	5.66	6.02	5.17		5.53	0.14	6/6
8 week Rx										
TAM16	3.69	3.49	3.79	4.06	3.43	4.08	4.12	3.81	0.11	7/7
RIF	3.04	3.24	3.81	4.12	3.13	3.07	2.62	3.29	0.19	7/7
TAM16+RIF	1.95	2.08	1.48	1.78	2.38	1.48	1.48	1.80	0.14	7/7
INH+RIF	1.57	< 1.57[b]	< 1.57	< 1.57	< 1.57	< 1.57	1.57	1.57	0.00	2/7
Vehicle	5.62	5.66	5.92	5.57	5.84	5.57		5.70	0.06	6/6

[a] No. of animals with CFU / no. of mice at sacrifice.

[b]<1.57; below limit of detection
